# Fluorous-Phase- and Chiral-Axis-Enhanced Fluorescent Sensitivity and Chemoselectivity for Cysteine Recognition

**DOI:** 10.1021/acs.orglett.4c04175

**Published:** 2025-01-06

**Authors:** Jiaqiao Yang, Cheng Qian, Hanyu Su, Ji Zhang, Shanshan Yu, Xiaoqi Yu, Lin Pu

**Affiliations:** † Key Laboratory of Green Chemistry and Technology, Ministry of Education, College of Chemistry, Sichuan University, Chengdu 610064, China; ‡ Asymmetric Synthesis and Chiral Technology Key Laboratory of Sichuan Province, Department of Chemistry, Xihua University, Chengdu 610039, China; § Department of Chemistry, University of Virginia, Charlottesville, Virginia 22904, United States

## Abstract

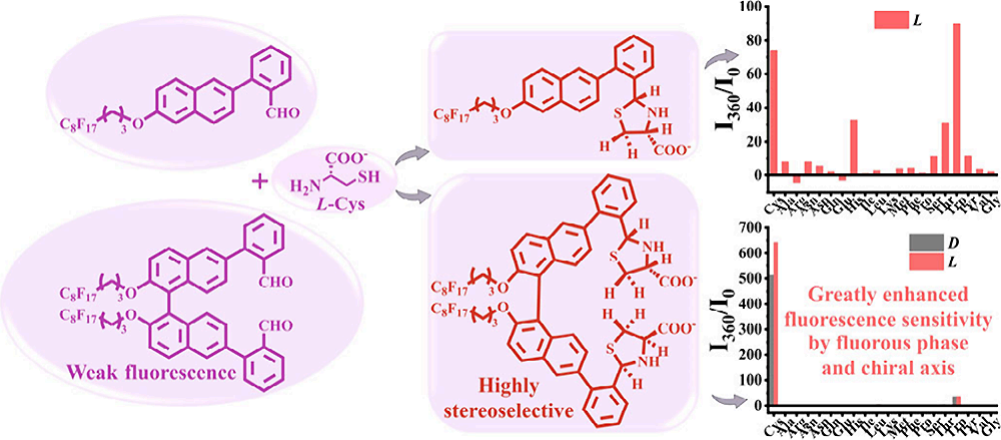

Highly fluorinated naphthyl aldehyde **1** and binaphthyl aldehyde (*R*)-**2** were designed and synthesized for fluorous-phase-based sensing. Greatly enhanced sensitivity and chemoselectivity in going from **1** to (*R*)-**2** in the fluorescent detection of cysteine has been discovered. This is attributed to the increased structural rigidity of the axially chiral binaphthyl unit in (*R*)-**2** upon reaction with cysteine to form the corresponding thiazolidine product. The fluorous-phase-based detection of cysteine not only can allow the analysis to be conducted in a phase away from the interference of other organic and inorganic species but also results in significantly increased fluorescence response.

Highly fluorinated materials can form a unique fluorous phase that is immiscible with common organic and aqueous phases. The affinity of the fluorous phase for highly fluorinated molecules allows them to be easily separated from other organic and inorganic species. Applications of the fluorous-phase-based separation techniques have been widely explored by researchers to develop more efficient processes in synthesis, catalysis, and others in the past few decades.^1,2^ Although the use of highly fluorinated molecular probes to conduct fluorous-phase-based molecular sensing can potentially minimize the interference of other organic and inorganic components and improve the accuracy and efficiency of the desired analysis,^3,4^ this research area is still underdeveloped.

In biological studies and chemical analysis, great progress has been made in the development of fluorescent probes for amino acids.^5^ Cysteine is a thiol-containing amino acid with important biological functions.^6^ It is a proteinogenic amino acid and can catalyze many important metabolic reactions. It can also serve as a biomarker for various diseases such as cardiovascular diseases, neurological disorders, diabetes, and cancers. A number of analytical methods have been utilized for the detection of cysteine, including the application of fluorescent probes,^7−9^ but no fluorous-phase-based selective detection of cysteine has been reported.

Among the fluorescent probes investigated for cysteine analysis, many of them contain an aldehyde receptor because the reaction of an aldehyde with cysteine can generate a cyclic thiazolidine product, leading to changes in the optical properties of the probe for signaling.^8,9^ Therefore, we designed compound **1**
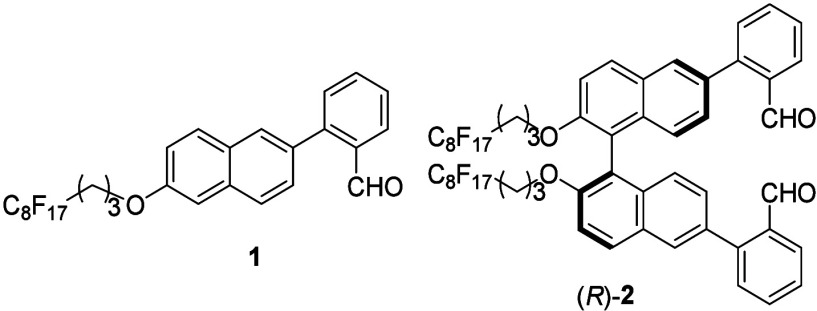
that contains a highly fluorinated alkyl group and an aldehyde unit for the fluorescent detection of cysteine in the fluorous phase. According to the density functional theory calculation, the HOMO of **1** is located on the electron-rich naphthyl unit and its LUMO is located on the electron-deficient benzaldehyde unit (see Figure S37). Upon excitation, there should be a photoinduced electron-transfer (PET) process between the naphthyl and benzaldehyde units to quench its fluorescence. It is proposed that when **1** reacts with cysteine to form the corresponding thiazolidine, the PET process will be inhibited because both the HOMO and LUMO of the thiazolidine product are located on the naphthyl fluorophore (see Figure S39). This should lead to fluorescence enhancement in the presence of cysteine. We have further designed axially chiral compound (*R*)-**2** whose two naphthyl benzaldehyde units might cooperate with each other to improve the fluorescent recognition of cysteine in the fluorous phase. We have discovered that although both **1** and (*R*)-**2** show fluorescence enhancement upon interaction with cysteine, greatly increased fluorescence sensitivity and chemoselectivity is observed from **1** to the axially chiral (*R*)-**2** in the fluorous phase. The use of the fluorous-phase-based probe (*R*)-**2** not only allows the fluorescence measurement to be conducted in a phase away from other organic and inorganic species to minimize their interference but also generates enhanced fluorescence sensitivity. The fluorescent recognition mechanism of (*R*)-**2** has been investigated. Herein, these results are reported.

Compounds **1** and (*R*)-**2** were synthesized and characterized as described in SI. Both compounds are soluble in fluorous solvents such as 2-(perfluorohexyl)ethanol (PEOH). Figure 1 compares the UV and fluorescence spectra of **1** and (*R*)-**2** in PEOH. The UV spectra give absorptions at λ (ϵ) = 233 (6.9 × 10^4^) and 313 (6.8 × 10^3^) nm for **1** and λ (ϵ) = 299 (9.4 × 10^3^) and 326 (7.8 × 10^3^) nm for (*R*)-**2**. The fluorescence spectra show very weak emissions at λ_em_ = 339 and 503 nm for **1** and λ_em_ = 326 and 508 nm for (*R*)-**2**.

**Figure 1:**
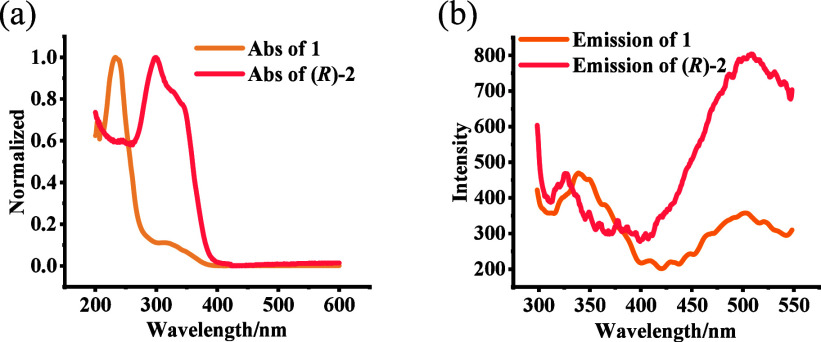
(a) UV spectra of **1** (2.5 × 10^–5^ M) and (*R*)-**2** (5.0 × 10^–5^ M). (b) Fluorescence spectra of **1** (5.0 × 10^–5^ M) and (*R*)-**2** (5.0 × 10^–5^ M). (Solvent: 99:1 v/v PEOH/CH_2_Cl_2_; λ_exc_ = 284 nm; slits, 20/20 nm.)

We studied the fluorescence response of **1** and (*R*)-**2** toward the tetrabutylammonium (TBA) salt of l-cysteine (l-Cys-TBA). A solution of **1** or (*R*)-**2** (5.0 × 10^–5^ M) in PEOH/CH_2_Cl_2_ was mixed with a MeOH solution of l-Cys-TBA (16.0 equiv) [98:1:1 v/v/v PEOH/MeOH/CH_2_Cl_2_] under vortexing and then allowed to stand in an incubator for 120 min before measurement. As shown in Figure 2a,b, both **1** and (*R*)-**2** show significant fluorescence enhancement in the presence of l-Cys-TBA. Figure 2c further demonstrates that (*R*)-**2** exhibits much greater fluorescence sensitivity than **1** when used to interact with cysteine.

**Figure 2:**
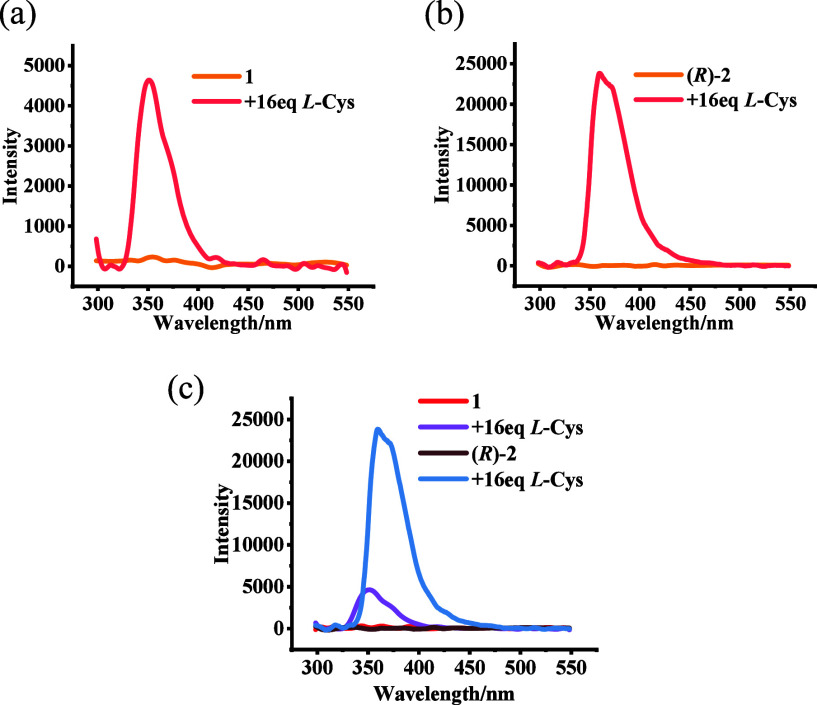
Fluorescence spectra of (a) **1** (5.0 × 10^–5^ M) and **1** + l-Cys-TBA (16.0 equiv), (b) (*R*)-**2** (5.0 × 10^–5^ M) and (*R*)-**2** + l-Cys-TBA (16.0 equiv), and (c) **1** and (*R*)-**2** (5.0 × 10^–5^ M) with 16.0 equiv of l-Cys-TBA in PEOH/MeOH/CH_2_Cl_2_ [98:1:1 v/v/v]. (λ_exc_ = 284 nm; slits, 5/5 nm; reaction time, 120 min at 25 °C.)

We studied the interaction of **1** with 20 common amino acid TBA salts in PEOH. As shown in Figure 3a, the fluorescence of **1** can be enhanced in the presence of several amino acids such as cysteine, histidine, threonine, and tryptophan. However, when (*R*)-**2** was treated with various common amino acid salts and their enantiomers, only cysteine could greatly enhance its fluorescence (Figure 3b). Thus, in going from **1** to (*R*)-**2** there is greatly increased sensitivity and chemoselectivity in the fluorescent recognition of cysteine. We also investigated the effects of the presence of other amino acids (Figure S20) and found that 14 of the 19 common amino acids (1 equiv) have only a small effect on the fluorescence response.

**Figure 3:**
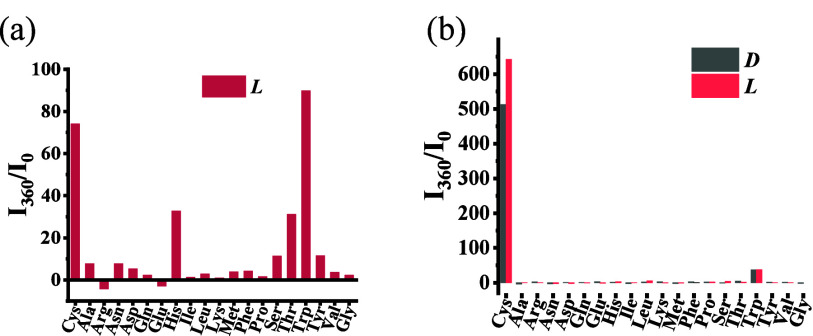
Fluorescence intensity at λ = 360 nm, *I*_360_/*I*_0_, from the interaction of 20 common amino acids (16.0 equiv) with (a) **1** (5.0 × 10^–5^ M) and (b) (*R*)-**2** (5.0 × 10^–5^ M) (Solvent: 98:1:1 v/v/v PEOH/MeOH/CH_2_Cl_2_; λ_exc_ = 284 nm; slits, 5/5 nm; reaction time, 120 min at 25 °C; *I*_0_: fluorescence intensity of **1** or (*R*)-**2** at 360 nm without amino acids).

The effect of the concentration of cysteine on the fluorescence response of (*R*)-**2** was studied. As shown in Figure 4, the fluorescence enhancement of (*R*)-**2** reached a maximum in the presence of 18 equiv of l-Cys-TBA. We chose 120 min as the reaction time, after which the fluorescence enhancement significantly decreased (see Figure S6). The limit of detection (LOD) and limit of quantification (LOQ) for l-Cys-TBA using (*R*)-**2** were determined to be 3.6 × 10^–9^ and 1.0 × 10^–5^ M respectively (see Figures S7–S9). The determination of the concentration of cysteine samples and cysteine in ternary mixtures of Cys, Hcy, and Met also shows the accuracy and significance of this assay (see Figures S10–S13 and Tables S1 and S2).

**Figure 4:**
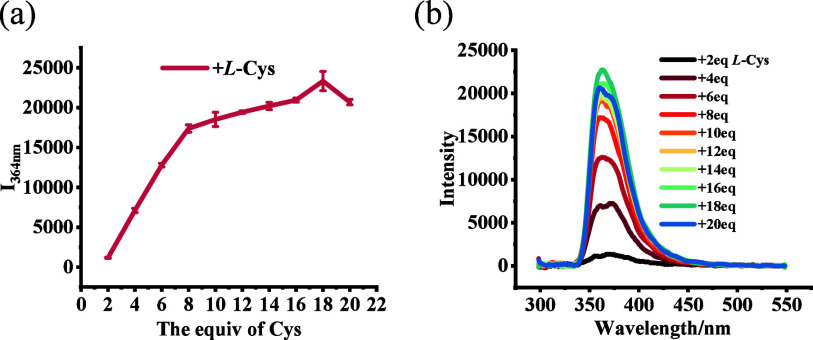
(a) Fluorescence intensity of (*R*)-**2** (5.0 × 10^–5^ M) at λ = 364 nm vs the number of equivalents of l-Cys-TBA. (b) Fluorescence spectra of (*R*)-**2** (5.0 × 10^–5^ M) with 2.0–20.0 equiv l-Cys-TBA. (Error bars from three independent experiments; solvent: 90:9:1 v/v/v PEOH/MeOH/CH_2_Cl_2_; λ_exc_ = 284 nm; slits, 5/5 nm; reaction time, 120 min at 25 °C.)

We conducted an ^1^H NMR titration of (*R*)-**2** with l-Cys-TBA in CDCl_3_/CD_3_OD (1:2) (see Figures S27–S29). When (*R*)-**2** was treated with over 4 equiv l-Cys-TBA, it was completely converted to a new compound with a new singlet appearing at δ 5.51 and three new doublet of doublets signals appearing at δ 3.57, 3.38, and 3.13, respectively, and the newly formed singlet at δ 5.51 showed a cross-peak with a peak at δ 68.2 in the ^1^H–^13^C 2D HSQC spectrum (see Figure S30a). This indicates the formation of thiazolidine product **4**. 
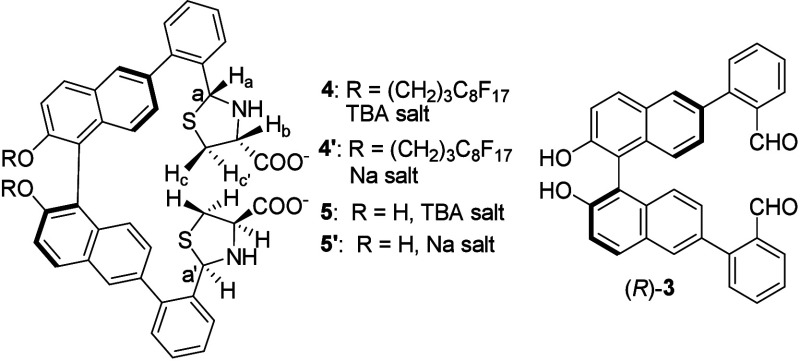
Formation of this product is also supported by the high-resolution ESI-MS analysis (see Figure S35). We also obtained compound **5** or **5′** from the reaction of l-Cys-TBA or l-Lys-Na with (*R*)-**3** that contains no fluorinated alkyl substituent (see the Supporting Information). The NMR spectra of **4** and **5** show that although two new sterogenic centers at carbons a and a′ were generated in the reaction with l-Cys, only one stereoisomer was produced. That is, the reaction of (*R*)-**2** or (*R*)-**3** with cysteine is highly stereoselective. Compounds **4′** and **5**/**5′** were characterized with 2D NMR spectroscopy, including HSQC, HMBC, COSY, and NOESY (see Figures S31–S33). The chiral configurations at carbons a and a′ were determined to be *R*, as NOE effects were observed between H_a_ and H_b_ and between H_a_ and H_c_ for **5′** (see Figure S32d).

We found that the fluorescence response of (*R*)-**2** toward l-Cys-Na, the sodium salt of cysteine, is almost the same as that observed for l-Cys-TBA (see Figure S14). That is, the thiazolidine products **4** and **4′** exhibit very similar fluorescence. We also studied the fluorescence response of the *in situ* -prepared Na salt product **4′** toward other metal cations (Figure 5). It was found that the addition of Mg^2+^ (2.0 equiv) tripled the fluorescence intensity of **4′** but other metal cations such as Li^+^, K^+^, and Ca^2+^ caused little effect on the fluorescence of **4′**.

**Figure 5:**
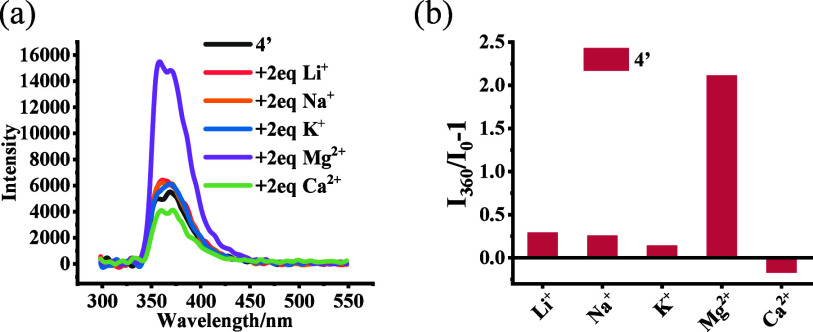
(a) Fluorescence spectra of **4′** (5.0 × 10^–5^ M) with a few metal ions (2.0 equiv). (b) Fluorescence intensity at λ = 360 nm, *I*_360_/*I*_0_ – 1, for the interaction of **4′** (5.0 × 10^–5^ M) with a few metal ions (2.0 equiv). (Solvent: 97:3 v/v PEOH/MeOH; λ_exc_ = 284 nm; slits, 5/5 nm; reaction time, 120 min at 25 °C; *I*_0_: fluorescence intensity of **4′** at 360 nm without metal ions.)

Very little fluorescence enhancement was observed when (*R*)-**2** was treated with other l-Cys derivatives in which the amine group or sulfhydryl group was protected (see Figure S15). When (*R*)-**2** was treated with 2-aminoethanethiol in PEOH, the fluorescence enhancement was much smaller (see Figure S15).

On the basis of the above results, we propose the following explanation for the greatly increased fluorescence sensitivity in going from **1** to (*R*)-**2**. When (*R*)-**2** is treated with cysteine, formation of the thiazolidine product **4** not only can inhibit the PET process the same as that proposed for **1** but also might increase the structural rigidity of the binaphthyl structure by limiting the relative rotation of the two naphthyl rings due to the increased steric bulkiness of the two thiazolidine rings. The observed significantly lower fluorescence enhancement for the reaction of (*R*)-**2** with 2-aminoethanethiol versus that with l-Cys-TBA (see Figure S15) also suggests that the larger size of the two thiazolidine units in **4** and their highly polar and ionic carboxylate units might contribute to the much greater fluorescence enhancement of **4** due to its more restricted relative rotation of the two naphthyl fluorophores. The observed further fluorescence enhancement of **4′** in the presence of Mg^2+^ (see Figure 5) might be attributed to a possible coordination of the two carboxylate groups in **4′** to a Mg^2+^ as shown in **6**, 
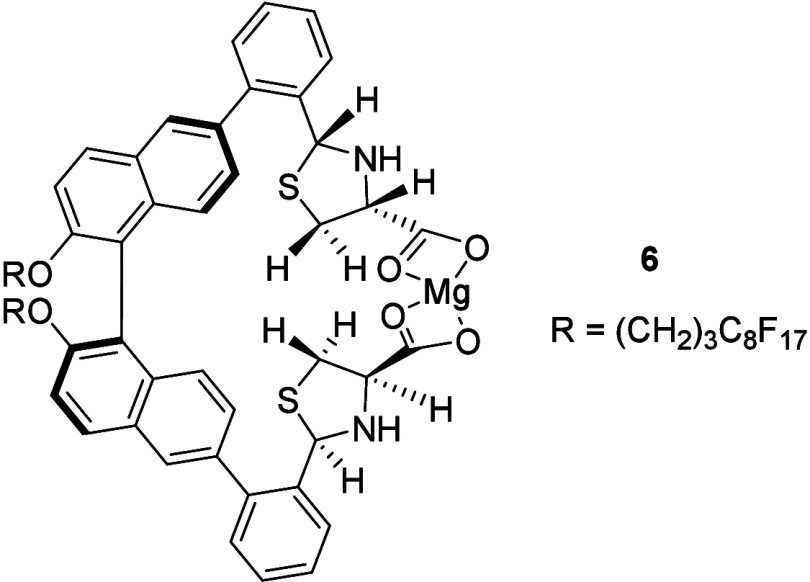
which can further restrict the relative rotation of the two naphthyl units and increase the structural rigidity of the fluorophores. Formation of such a complex is also supported by high-resolution ESI-MS analysis (see Figure S36).

We synthesized compounds (*R*)-**7** and (*R*)-**8**
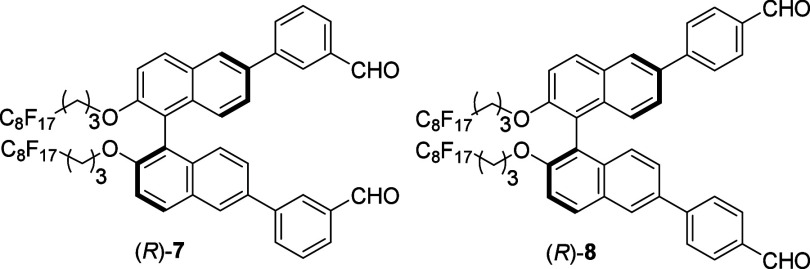
that contain *m*- and *p*-benzaldehyde units respectively, and compared their fluorescence response toward cysteine with that of (*R*)-**2** in the fluorous phase. As shown in Figure 6, when these compounds were treated with l-Cys-TBA, the observed fluorescence enhancements are (*R*)-**2** > (*R*)-**7** > (*R*)-**8**. That is, the probe with *o*-benzaldehyde units displays much greater fluorescence enhancement than those with *m*- and *p*-benzaldehydes. It is proposed that upon reaction with cysteine, the *o*-benzaldehyde units of (*R*)-**2** can generate the two thiazolidine units in **4** which are close to the binaphthyl units and each other. However, the two thiazolidine units in the products formed from the reaction of (*R*)-**7** and (*R*)-**8** with cysteine should be farther away from the binaphthyl units and each other, generating more extended and less packed structures. This should lead to smaller steric interaction, easier rotation, and less structural rigidity, giving the observed weaker fluorescence enhancement.

**Figure 6:**
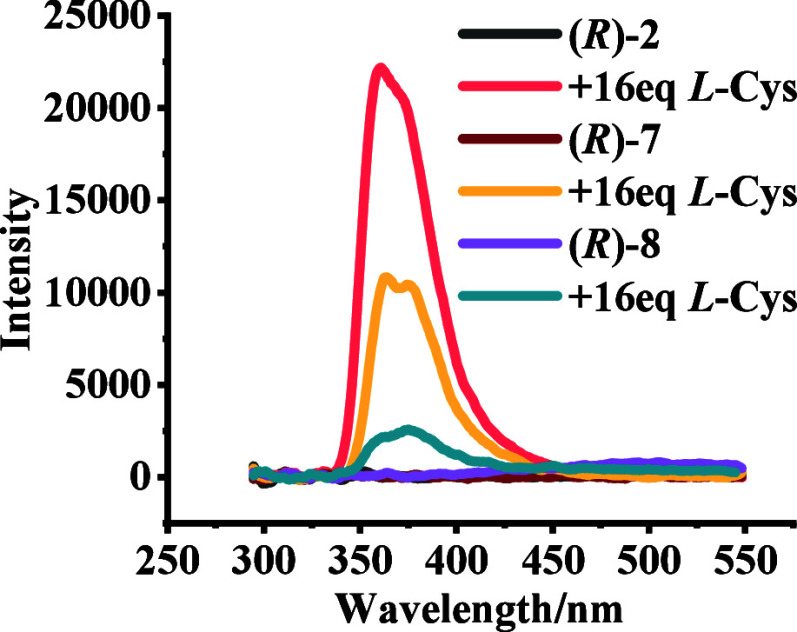
Fluorescence spectra of (*R*)-**2**, (*R*)-**7**, and (*R*)-**8** (5.0 × 10^–5^ M) with l-Cys-TBA (16.0 equiv). (Solvent: 98:1:1 v/v/v PEOH/MeOH/CH_2_Cl_2_; λ_exc_ = 284 nm; slits, 5/5 nm; reaction time, 120 min at 25 °C.)

We also studied the fluorescence response of the non-fluorinated compound (*S*)-**3** (5.0 × 10^–5^ M) toward l-Cys-TBA in a non-fluorous phase [99:1 v/v MeOH/CH_2_Cl_2_] and compared it with that of the fluorinated compound (*S*)-**2** in the fluorous phase. As shown in Figure 7, the fluorescence enhancement of (*S*)-**3** with l-Cys-TBA in MeOH at λ_em_ = 360 nm was much weaker than that when (*S*)-**2** was treated with l-Cys-TBA in PEOH. Thus, the fluorous phase is essential for the high sensitivity of the highly fluorinated probe (*S*)-/(*R*)-**2** in the fluorescent recognition of cysteine.

**Figure 7:**
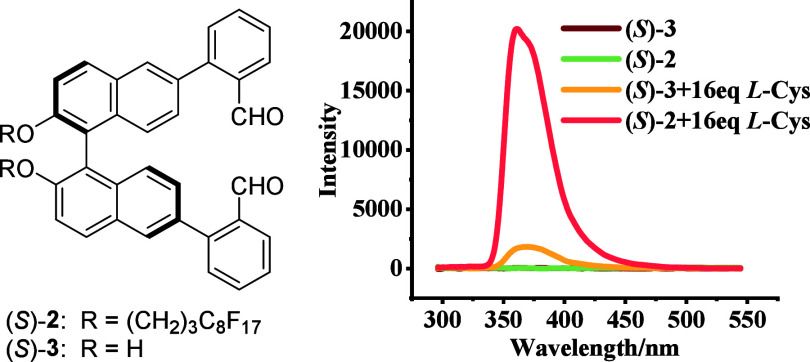
Fluorescence spectra of (*S*)-**3** (5.0 × 10^–5^ M) in MeOH/CH_2_Cl_2_ [99:1 v/v] and (*S*)-**2** (5.0 × 10^–5^ M) in PEOH/MeOH/CH_2_Cl_2_ [98:1:1 v/v/v] with and without l-Cys-TBA (16.0 equiv). (λ_exc_ = 284 nm; slits, 5/5 nm; reaction time, 120 min at 25 °C.)

We compared the fluorescence responses of (*R*)-**2** toward l-Cys-TBA in PEOH with those in other non-fluorous solvents. As shown in Figure 8, the fluorescence enhancement in the fluorous phase is much greater than that in other solvents. This could be attributed to the strong lipophobic properties of the highly fluorinated solvent. The highly fluorinated alkyl groups of the thiazolidine product **4** formed from the reaction of (*R*)-**2** with l-Cys-TBA can make this compound soluble in the fluorous phase, but the non-fluorinated binaphthyl part should not interact favorably with the highly fluorinated solvent, increasing the structural rigidity of this compound. This should contribute to the observed greater fluorescence enhancement. The interaction of (*R*)-**2** with other biothiols shows that homocysteine also greatly enhanced its fluorescence, which could be ascribed to the formation of a six-membered-ring thiazinane product similar to cysteine (see Figure S16). Other biothiols have little effect on the dialdehyde and the thiazolidine formation, except for GSH. Addition of GSH into a mixture of (*R*)-**2** and Cys leads to a decrease in fluorescence (see Figures S17–S18). The effect of the GSH concentration on the fluorescence response of (*R*)-**2** + Cys shows that the fluorescence remains stable over 10 equiv of GSH (see Figure S19).

**Figure 8:**
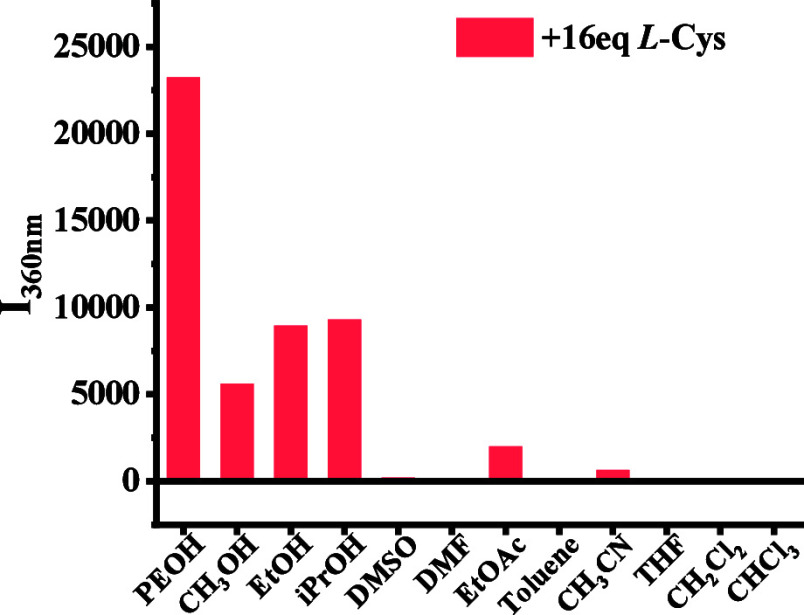
Fluorescence intensity at λ = 364 nm (*I*_364_) for the interaction of (*R*)-**2** (5.0 × 10^–5^ M) with l-Cys-TBA (16.0 equiv) in various organic solvents (0.5 mL). (λ_exc_ = 284 nm; slits, 5/5 nm; reaction time, 120 min at 25 °C.)

In conclusion, we have designed and synthesized two highly fluorinated probes, **1** and (*R*)-**2**, for fluorescent recognition of cysteine in the fluorous phase. We have discovered that the binaphthyl probe (*R*)-**2** exhibits high fluorescence sensitivity and chemoselectivity in the fluorous-phase-based detection of cysteine which are much greater than those of the mononaphthyl probe **1**. It is found that the observed large fluorescence enhancement of (*R*)-**2** upon interaction with cysteine can be attributed to the reaction of the aldehyde groups of the probe to form the corresponding thiazolidine product. The flexible binaphthyl structure of (*R*)-**2** can achieve higher structural rigidity after the formation of the thiazolidine product since the increased steric hindrance can limit the relative rotation of the axially chiral binaphthyl unit. This binaphthyl structure should contribute to the greatly enhanced fluorescence in addition to inhibiting the PET process between the electron-rich naphthyl fluorophore and the electron-deficient benzaldehyde group. It is also found that the fluorous solvent not only can allow the detection to be conducted in an environment separated from common organic and aqueous phases but also can greatly enhance the fluorescence response. This study further demonstrates the great potential of fluorous-phase-based fluorescent probes in molecular recognition.

## Data Availability

The data underlying this study are available in the published article and its Supporting Information.

## Supporting Information Available

Experimental procedures, NMR spectra for all compounds, additional spectroscopic data, and molecular modeling study (PDF)
