# Supplementary material for: Fluorous-Phase- and Chiral-Axis-Enhanced Fluorescent Sensitivity and Chemoselectivity for Cysteine Recognition
Source: Org Lett. 2025 Jan 6;27(2):571–6. doi: 10.1021/acs.orglett.4c04175 (PMC11744791; doi:10.1021/acs.orglett.4c04175)
Supplement: Supplementary file 1 — ol4c04175_si_001.pdf [file ol4c04175_si_001.pdf]

Supporting Information

Fluorous Phase and Chiral Axis-Enhanced Fluorescent Sensitivity and Chemoselectivity for Cysteine Recognition

Jiaqiao Yang,[a] Cheng Qian,[a] Hanyu Su,[a]
Ji Zhang,[a] Shanshan Yu,*[a] Xiaoqi Yu[a,b] and Lin Pu*[c]

[a] Key Laboratory of Green Chemistry and Technology, Ministry of Education, College of Chemistry, Sichuan University, Chengdu, China 610064.

[b] Asymmetric Synthesis and Chiral Technology Key Laboratory of Sichuan Province, Department of Chemistry, Xihua University, Chengdu 610039, P. R. China.

[c] Department of Chemistry, University of Virginia, McCormick Rd, Charlottesville VA 22904.

E-mail: lp6n@virginia.edu; yushanshan@scu.edu.cn

Contents

1. General information of the materials, instruments, syntheses and characterization----------------------S2

2. Optical measurements---S29

3. NMR spectroscopic study---S54

4. Mass spectra--S64

5. Fluorescence quantum yield measurements--S66

6. Molecular modeling study---S67

7. References---S72

1. General information of the materials, instruments, syntheses and characterization.

1.1. Materials. All chemicals and reagents were analytical-grade and with no further purification from suppliers. In the optical measurements, all solvents were either HPLC or spectroscopic grade. Analytical grade solvents and all reagents were purchased from Energy Chemical Co. Ltd. (Shanghai, China) and InnoChem Science & Technology Co. Ltd. (Beijing, China). Fluorinated reagents and solvents were purchased from Qingba Chemical Co. Ltd. (Shanghai, China).

1.2. Instruments. ^{1}H NMR, ^{13}C NMR, 2D HSQC NMR, 2D HMBC, H-H COSY and NOESY spectra were measured on a Bruker AM400 NMR spectrometer. Structural assignments were made with additional information from gCOSY, gHSQC, and gHMBC experiments. High-resolution mass spectra (HRMS) were obtained by using a SHIMADZU LCMS-IT-TOF (IT-TOF analyzer) mass spectrometer by ESI techniques. Fluorescence spectra were obtained by using a Horiba Duetta fluorescence and absorbance spectrometer. UV-Vis absorption spectra were recorded on a Hitachi U1900 spectrometer. Fluorescence quantum yield (Φ) measurement was conducted on Horiba JoblnYvon-Edision Fluorolog-3.

1.3. Synthesis and characterization.

Compound (*R*)-/(*S*)-**9** was synthesized according to the literature procedures. [S1-S4] Compound **13** was synthesized according to a previously reported procedure. [S5]

1.3.1. Synthesis and characterization of compound **1**.

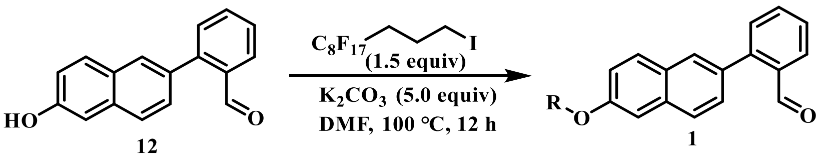

R=$CH_2CH_2CH_2C_8F_{17}$

Compound **12** (1.0 mmol, 0.2483 g, 1.0 equiv) and K_2CO_3 (5.0 mmol, 0.6611 g, 5.0 equiv) were stirred in DMF (6 mL) at 100 °C (oil bath) for 30 min. Next 3-(perfluorooctyl)propyl iodide (1.50 mmol, 0.8821 g, 1.5 equiv) in DMF (2 mL) was added slowly. The reaction mixture was then stirred at 100 °C (oil bath) for 12 h, which was filtered and concentrated under reduced pressure after the reaction was complete. The residue was purified by flash column chromatography on silica gel (eluted with petroleum ether/ethyl acetate, 25/1, v/v) to afford compound **1** (0.6407 g) as a light-yellow solid in 90% yield. ^{1}H NMR (400 MHz, Chloroform-*d*) δ 10.03 (s, 1H), 8.07 (d, *J* = 7.84 Hz, 1H), 7.81 (t, *J* = 8.92 Hz, 2H), 7.76 (s, 1H), 7.68 (t, *J* = 7.24 Hz, 1H), 7.52 (m, 3H), 7.24 (d, *J* = 8.80 Hz, 1H), 7.19 (s, 1H), 4.21 (t, *J* = 5.84 Hz, 2H), 2.39 (m, 2H), 2.21 (m, 2H). ^{13}C{^{1}H} NMR (100 MHz, Chloroform-*d*) δ 192.8, 157.3, 146.1, 134.1, 134.0, 133.7, 133.2, 131.2, 130.0, 129.4, 128.8, 128.5, 127.8, 127.1, 119.9, 118.8-107.9 (m), 106.6, 66.6, 28.2 (t, *J* = 22.00 Hz), 20.7. ^{19}F NMR (376 MHz, Chloroform-*d*) δ -80.71 (t, *J* =9.55 Hz, 3F), -114.30 (m, 2F), -121.82 (m, 6F), -122.69 (m, 2F), -123.35 (m, 2F), -126.08 (m, 2F). HRMS (ESI-IT-TOF) m/z: $[M+H]^+$ Calcd for $C_{28}H_{17}F_{17}NaO_2^+$ 731.0849; Found: 731.0846. mp: 96.6-97.1 °C.

^{1}H NMR (400 MHz, Chloroform-*d*) spectrum of **compound 1**

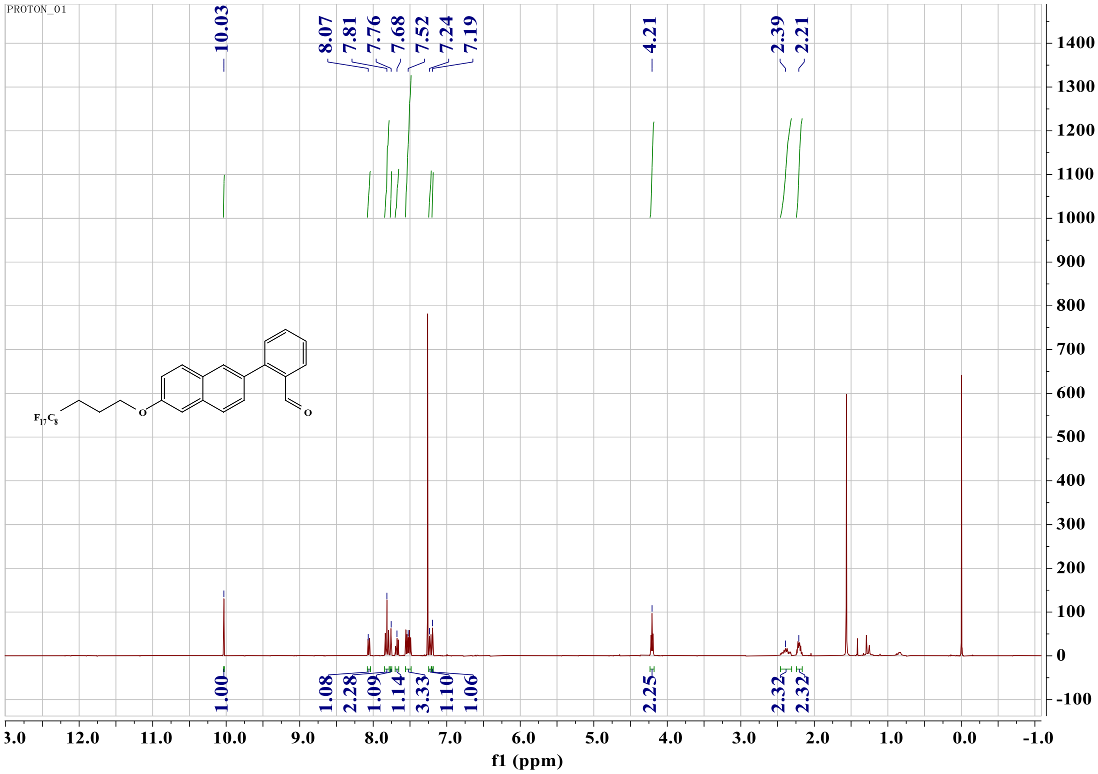

^{13}C{^{1}H} NMR (100 MHz, Chloroform-*d*) spectrum of **compound 1**

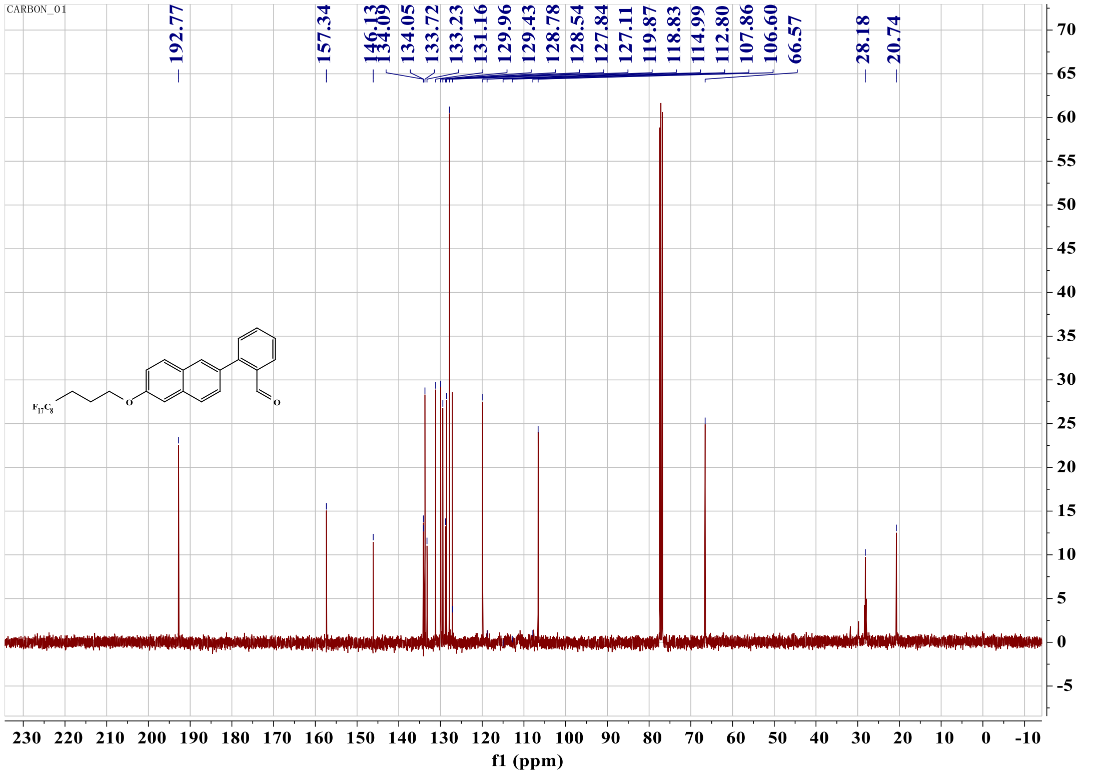

^{19}F NMR (376 MHz, Chloroform-*d*) spectrum of compound **1**

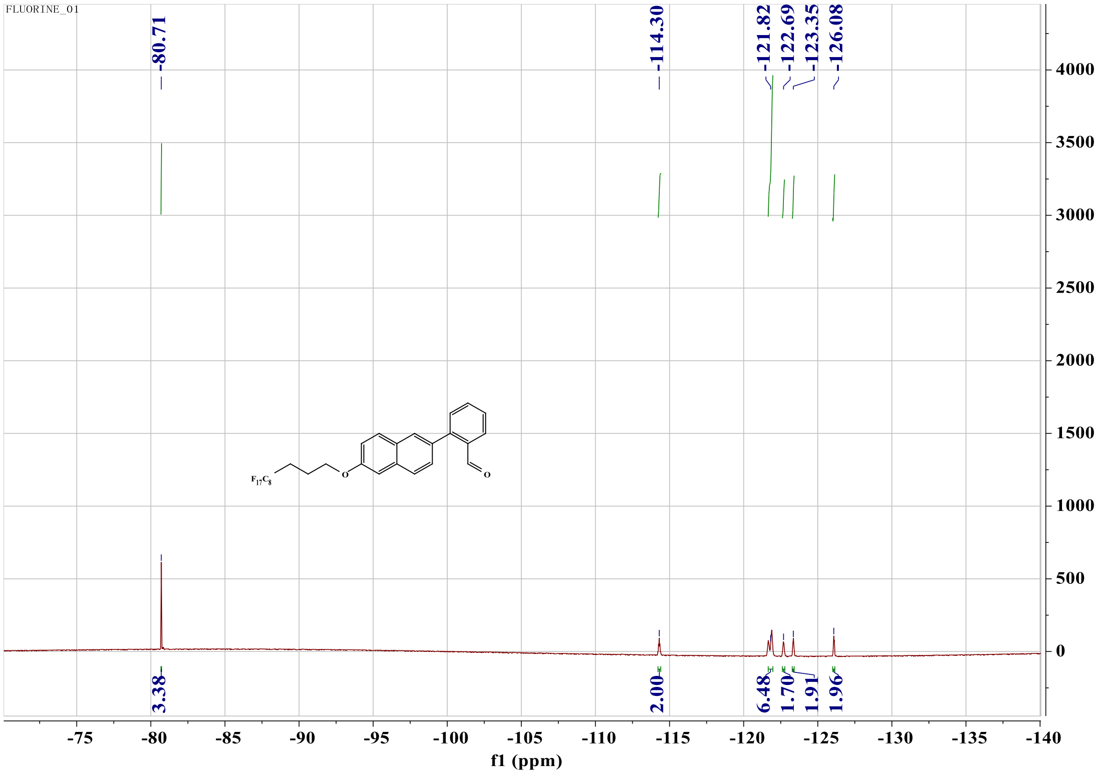

1.3.2. Synthesis and characterization of (*R*)-**2**.

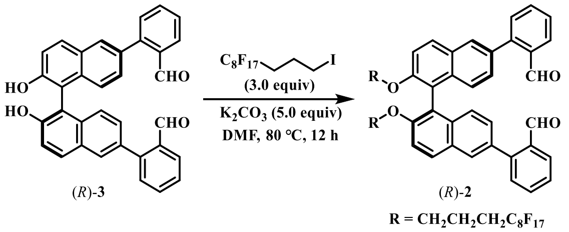

(*R*)-**3** (0.57 mmol, 0.2819 g, 1.0 equiv) and K_2CO_3 (2.85 mmol, 0.3768 g, 5.0 equiv) were stirred in DMF (6 mL) at 80 °C (oil bath) for 30 min. Next 3-(perfluorooctyl)propyl iodide (1.70 mmol, 1.0000 g, 3.0 equiv) in DMF (2 mL) was added slowly. The reaction mixture was then stirred at 80 °C (oil bath) for 12 h, which was filtered and concentrated under reduced pressure after the reaction was complete. The residue was purified by flash column chromatography on silica gel (eluted with petroleum ether/ethyl acetate, 10/1, v/v) to afford (*R*)-**2** (0.8064 g) as a yellow solid in 84% yield. ^{1}H NMR (400 MHz, Chloroform-*d*) δ 10.05 (s, 2H), 8.04 (m, 4H), 7.88 (s, 2H), 7.65 (t, *J* = 7.72 Hz, 2H), 7.52 (m, 6H), 7.35 (d, *J* = 8.80 Hz, 2H), 7.30 (d, *J* = 8.60 Hz, 2H), 4.14 (m, 2H), 4.04 (m, 2H), 1.71 (m, 8H). $^{13}C\{^{1}H\}$ NMR (100 MHz, Chloroform-*d*) δ 192.5, 154.4, 145.8, 133.8, 133.5, 133.3, 133.3, 130.9, 130.1, 129.7, 129.1, 128.6, 127.7, 127.6, 125.4, 120.1, 116.1, 120.8-108.6 (m), 68.1, 27.2 (t, *J* = 22.01 Hz), 20.6. ^{19}F NMR (376 MHz, Chloroform-*d*) δ -80.78 (t, *J* =9.55 Hz, 6F), -114.52 (m, 4F), -122.02 (m, 12F), -122.80 (m, 4F), -123.46 (m, 4F), -126.18 (m, 4F). HRMS (ESI-IT-TOF) m/z: $[M+Na]^+$ Calcd for $C_{56}H_{32}F_{34}NaO_4^+$ 1437.1650; Found: 1437.1639. $[\alpha]^{26.0}_{589}$ = +9.2 (c= 0.1, in $CHCl_3$). mp: 68.6-69.4 °C.

^{1}H NMR (400 MHz, Chloroform-*d*) spectrum of (*R*)-**2**

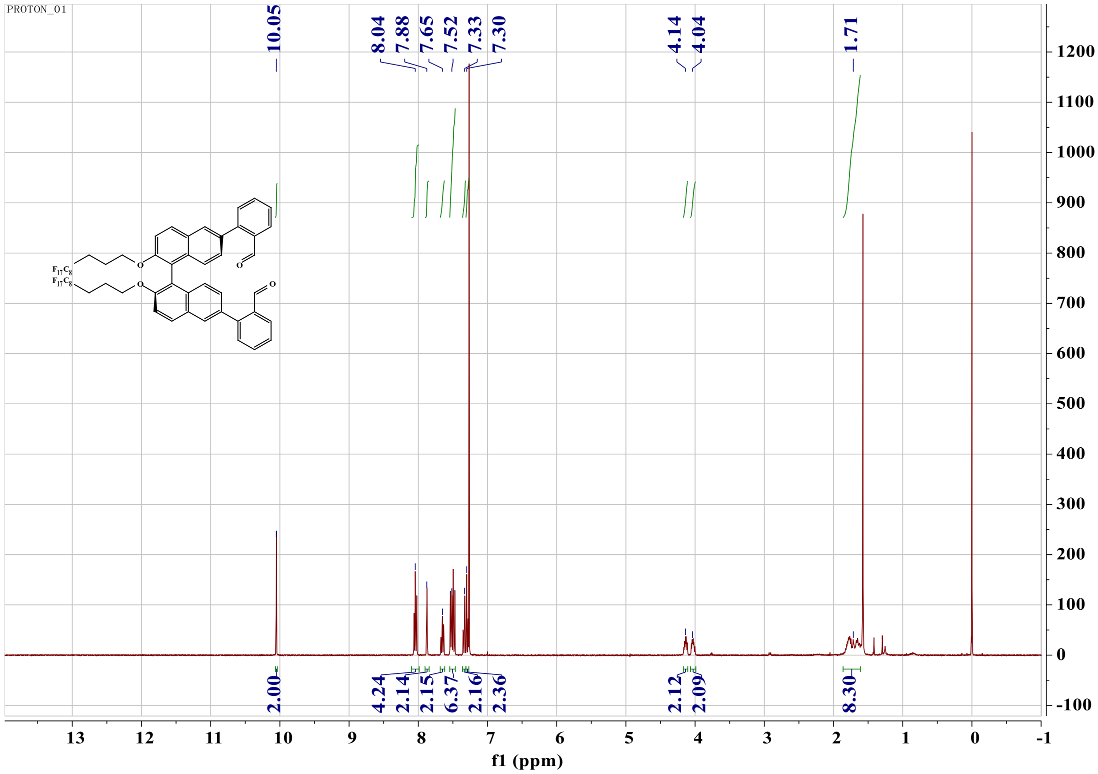

^{13}C{^{1}H} NMR (100 MHz, Chloroform-*d*) spectrum of (*R*)-**2**

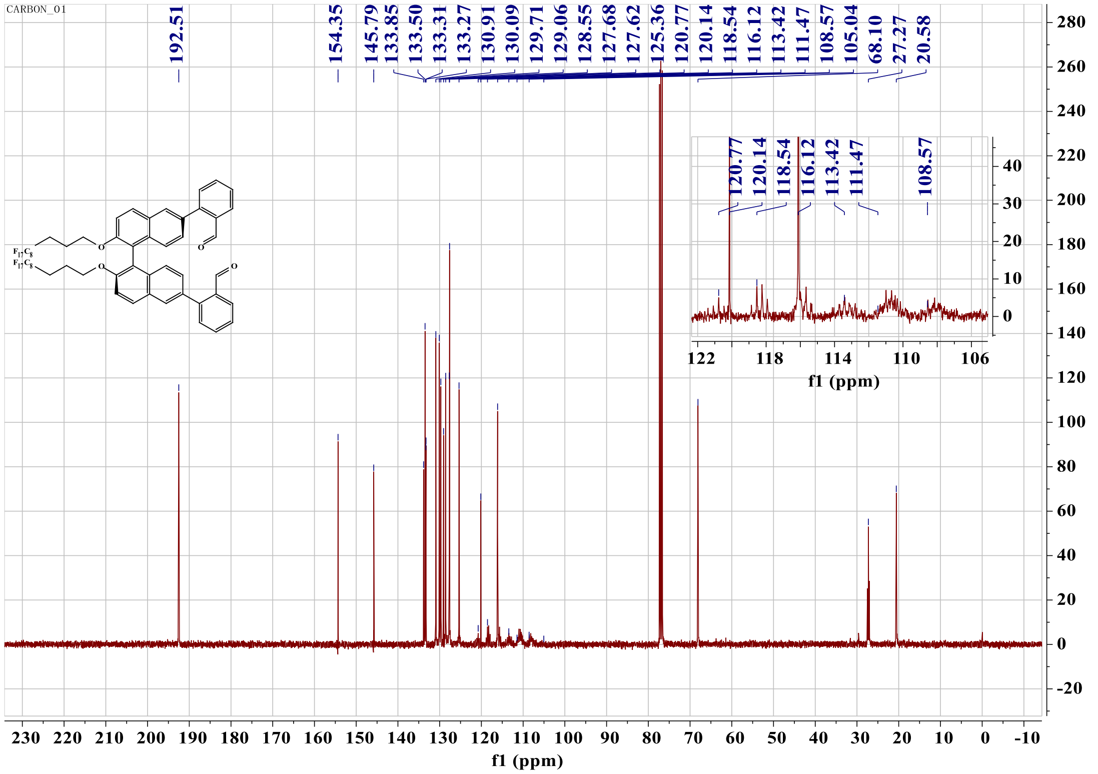

^{19}F NMR (376 MHz, Chloroform-*d*) spectrum of (*R*)-**2**

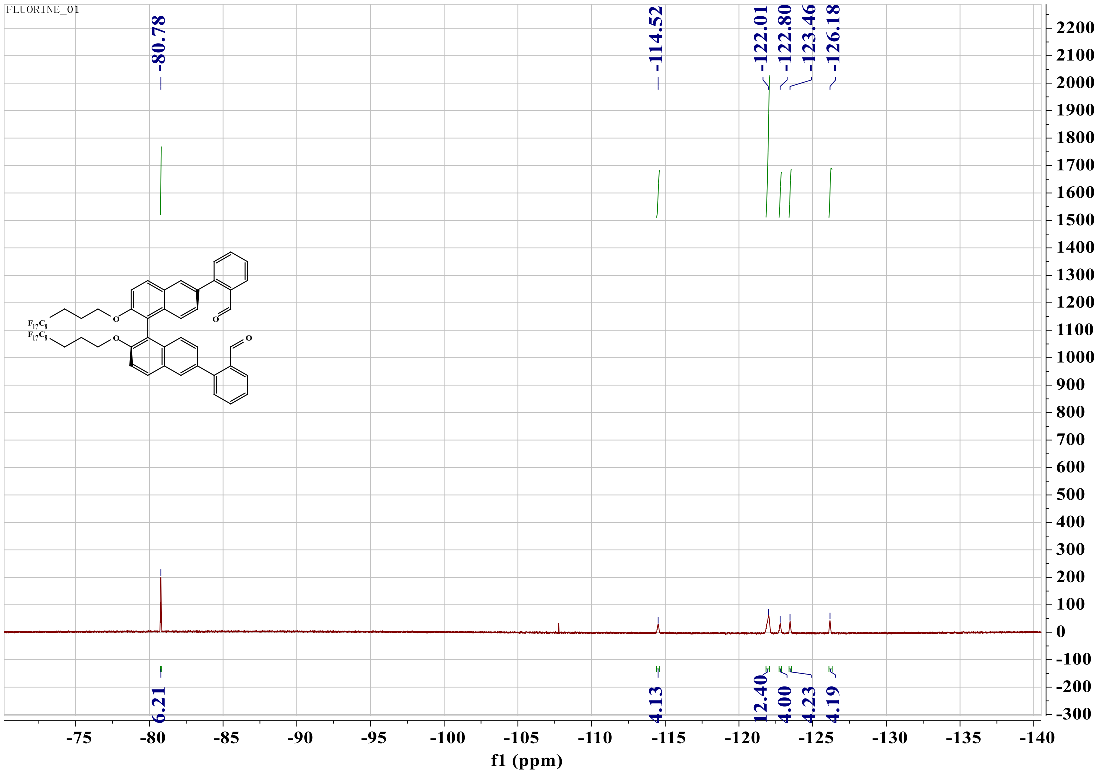

1.3.3. Synthesis and characterization of (*S*)-**2**.

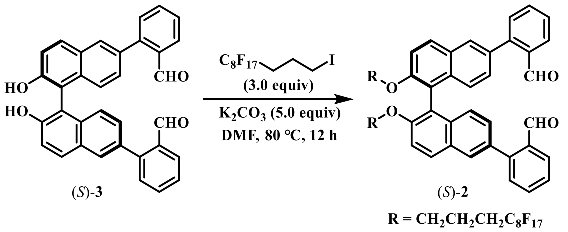

(*S*)-**2** (0.7658 g) was purified by flash column chromatography on silica gel (eluted with petroleum ether/ethyl acetate, 10/1, v/v) to afford a yellow amorphous solid in 97% yield in the same way as (*R*)-**2** by starting with (*S*)-**3** (0.56 mmol, 0.2770 g, 1.0 equiv). ^{1}H NMR (400 MHz, Chloroform-*d*) δ 10.05 (s, 2H), 8.04 (m, 4H), 7.87 (s, 2H), 7.65 (t, *J* = 7.72 Hz, 2H), 7.49 (m, 6H), 7.35 (d, *J* = 8.60 Hz, 2H), 7.30 (d, *J* = 8.64 Hz, 2H), 4.12(m, 2H), 4.03(m, 2H), 1.71(m, 8H). ^{13}C{^{1}H} NMR (100 MHz, Chloroform-*d*) δ 192.6, 154.3, 145.8, 133.8, 133.5, 133.3, 133.2, 130.9, 130.1, 129.8, 129.0, 128.6, 127.7, 127.6, 125.4, 120.1, 116.1, 118.9-108.4 (m), 68.1, 27.2 (t, *J* = 22.38 Hz), 20.6. ^{19}F NMR (376 MHz, Methanol-*d*4) δ -80.78 (t, *J* = 9.55 Hz, 6F), -114.51 (m, 4F), -121.99 (m, 12F), -122.79 (m, 4F), -123.47 (m, 4F), -126.18 (m, 4F). HRMS (ESI-IT-TOF) m/z: $[M+Na]^+$ Calcd for $C_{56}H_{32}F_{34}NaO_4^+$ 1437.1650; Found: 1437.1687. $[\alpha]^{26.0}_{589}$ = -12.0 (c= 0.1, in $CHCl_3$). mp: 68.1-68.9 °C.

^{1}H NMR (400 MHz, Chloroform-*d*) spectrum of (*S*)-**2**

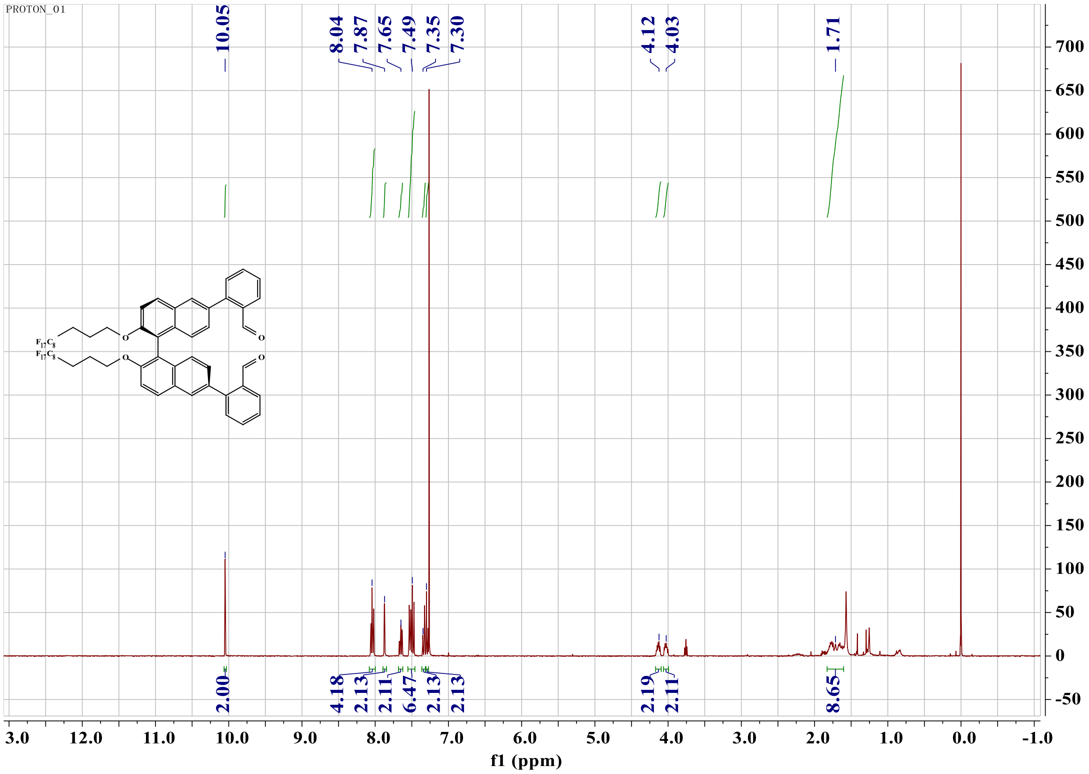

$^{13}C\{^{1}H\}$ NMR (100 MHz, Chloroform-*d*) spectrum of (*S*)-**2**

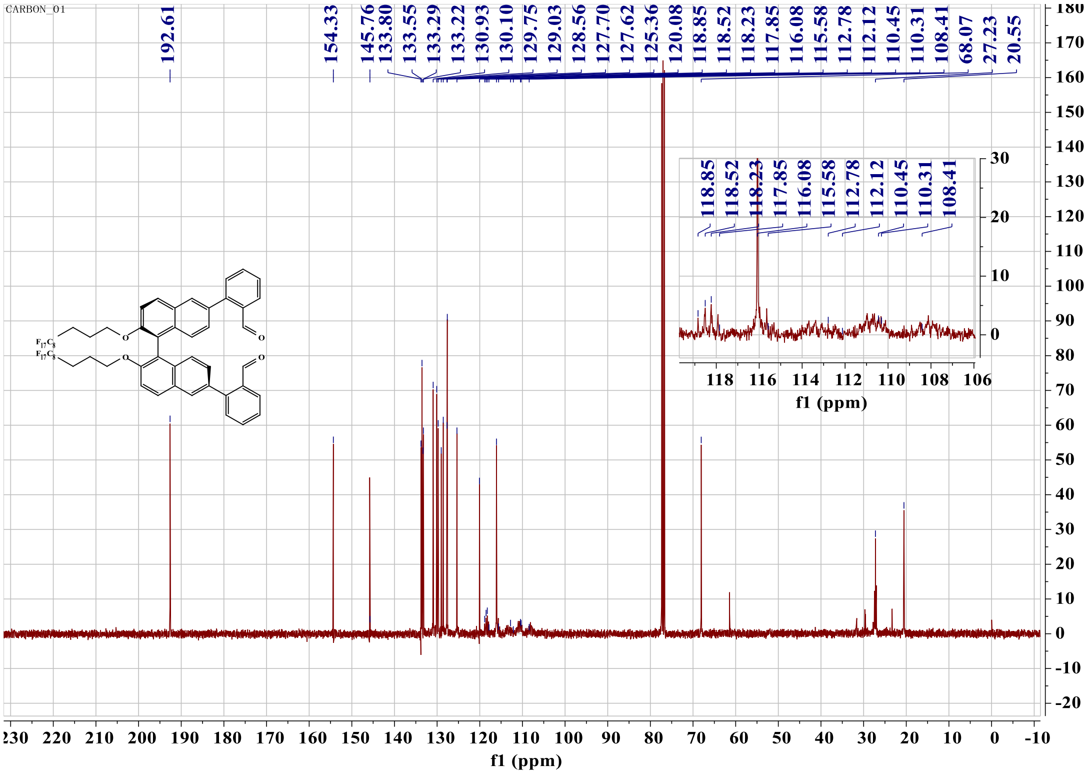

^{19}F NMR (376 MHz, Chloroform-*d*) spectrum of (*S*)-**2**

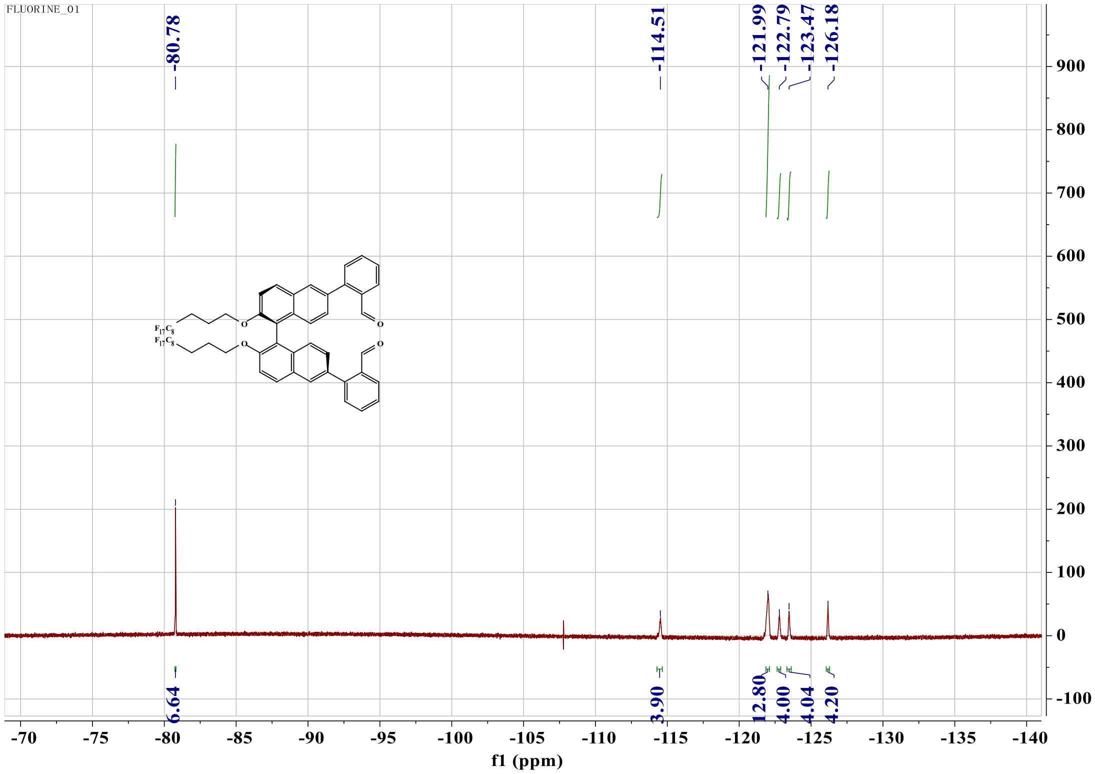

1.3.4. Synthesis and characterization of (*R*)-**3**.

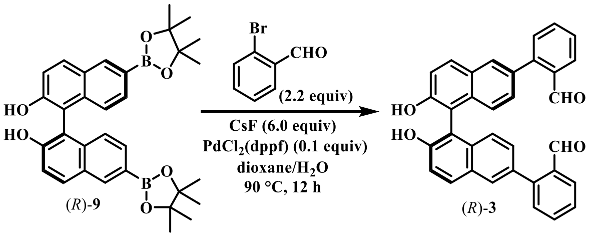

Under argon atmosphere, compound (*R*)-**9** (5.0 mmol, 2.6913 g, 1.0 equiv), 2-bromobenzaldehyde (11.0 mmol, 2.0352 g, 2.2 equiv), Pd(dppf)Cl_2 (0.5 mmol, 0.3659 g, 0.1 equiv) and CsF (30.0 mmol, 4.5571 g, 6.0 equiv) were dissolved in deoxidized dioxane/water (150 mL, 2/1, v/v). Stirred at 90 °C (oil bath) for 12 h, the reaction mixture was cooled to room temperature and extracted with ethyl acetate (150 mL). The organic layer was washed with saturated brine, dried with anhydrous Na_2SO_4, filtered and concentrated under reduced pressure. The residue was purified by flash column chromatography on silica gel (eluted with petroleum ether/ethyl acetate, 3/1, v/v) to afford (*R*)-**3** (1.1445 g) as an orange solid in 46% yield. ^{1}H NMR (400 MHz, Chloroform-*d*) δ 10.03 (s, 2H), 8.04 (m, 4H), 7.89 (s, 2H), 7.67 (t, *J* = 7.52 Hz, 2H), 7.52 (m, 4H), 7.49 (d, *J* = 9.00 Hz, 2H), 7.39 (d, *J* = 8.60 Hz, 2H), 7.31 (d, *J* = 8.60 Hz, 2H), 5.35 (s, 2H). $^{13}C\{^{1}H\}$ NMR (100 MHz, Chloroform-*d*) δ 192.6, 153.5, 145.6, 133.8, 133.7, 133.3, 133.0, 131.6, 131.0, 130.0, 129.3, 129.0, 127.9, 127.8, 124.6, 118.9, 111.2. HRMS (ESI-IT-TOF) m/z: $[M+Na]^+$ Calcd for $C_{34}H_{22}NaO_4^+$ 517.1410; Found: 517.1411. $[\alpha]^{26.0}_{589}$ = -168.8 (c= 0.1, in $CHCl_3$). mp: 176.3-177.0 °C.

^{1}H NMR (400 MHz, Chloroform-*d*) spectrum of (*R*)-**3**

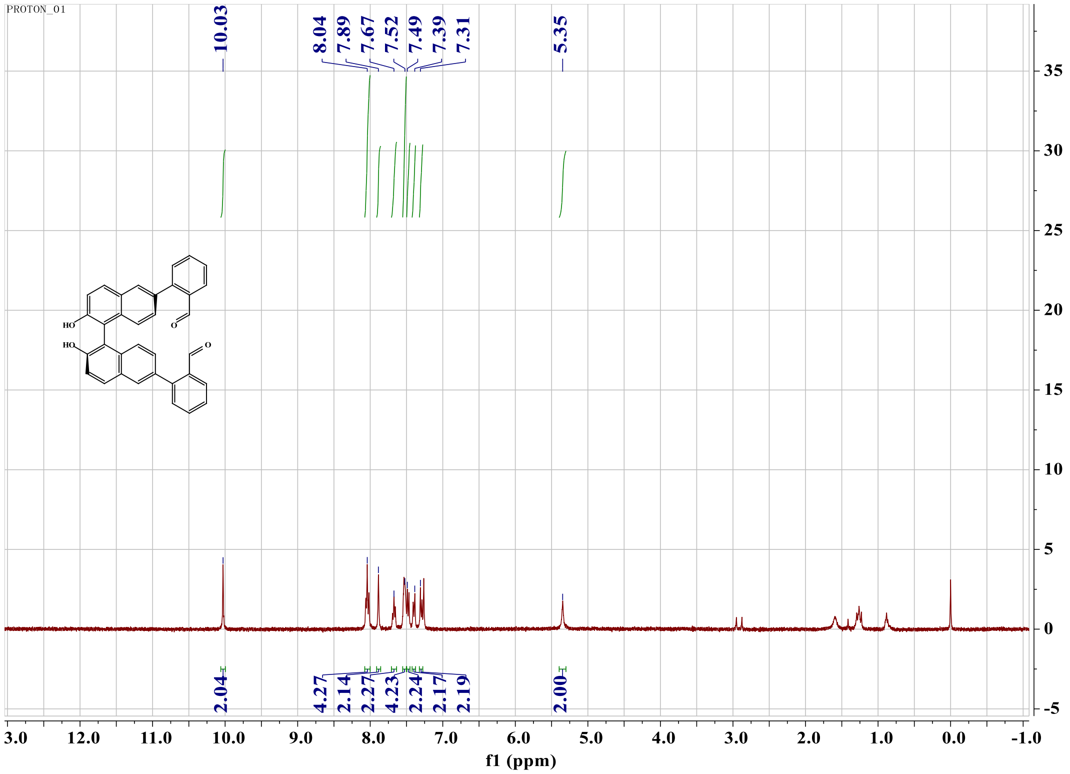

^{13}C{^{1}H} NMR (100 MHz, Chloroform-*d*) spectrum of (*R*)-**3**

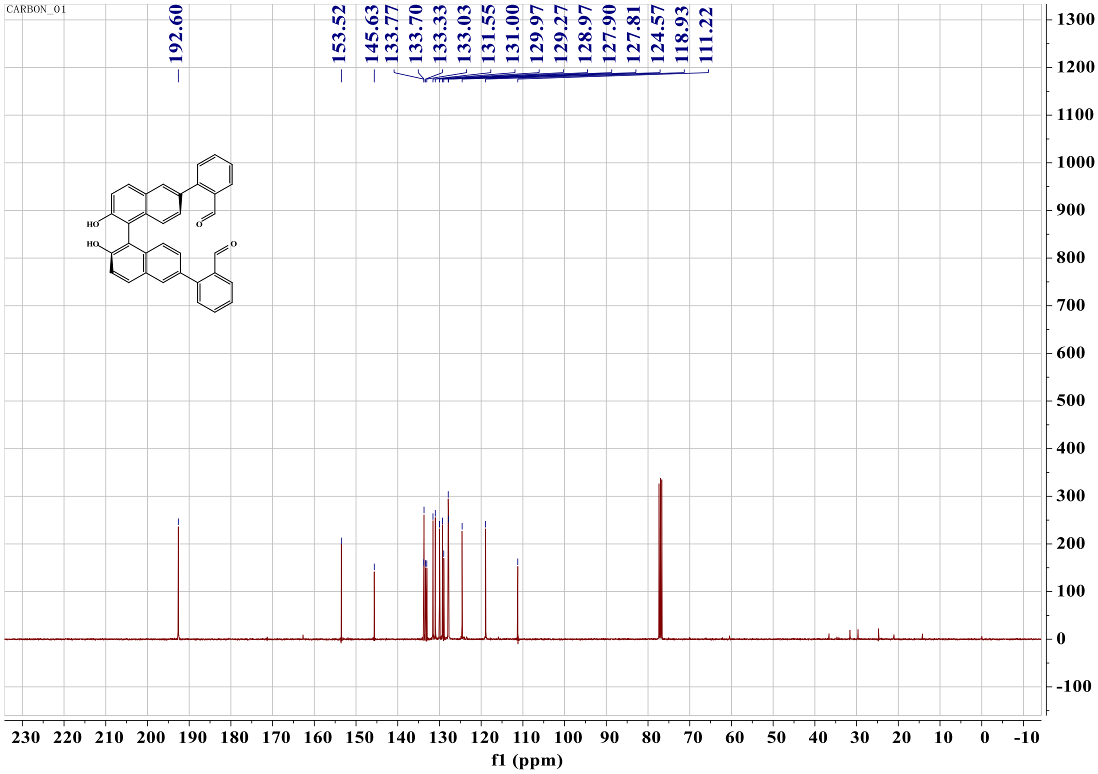

1.3.5. Synthesis and characterization of (*S*)-**3**.

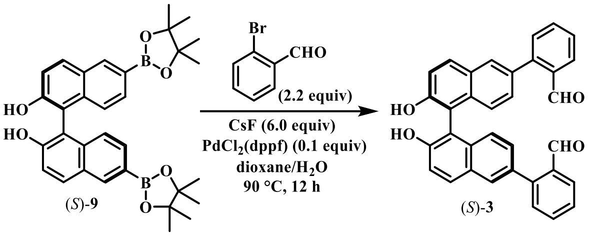

(*S*)-**3** (1.1191 g) was purified by flash column chromatography on silica gel (eluted with petroleum ether/ethyl acetate, 3/1, v/v) to afford an orange amorphous solid in 42% yield in the same way as (*R*)-**3** by starting with (*S*)-**9** (5.4 mmol, 2.9086 g, 1.0 equiv) and 2-bromobenzaldehyde (11.9 mmol, 2.1908 g, 2.2 equiv). ^{1}H NMR (400 MHz, Chloroform-*d*) δ 10.03 (s, 2H), 8.05 (m, 4H), 7.89 (s, 2H), 7.68 (t, *J* = 7.44 Hz, 2H), 7.53 (m, 4H), 7.49 (d, *J* = 9.00 Hz, 2H), 7.41 (d, *J* = 8.64 Hz, 2H), 7.31 (d, *J* = 8.60 Hz, 2H), 5.31 (s, 2H). ^{13}C{^{1}H} NMR (100 MHz, Chloroform-*d*) δ 192.4, 153.5, 145.5, 133.8, 133.6, 133.5, 132.9, 131.8, 131.0, 130.0, 129.4, 129.1, 127.8, 124.5, 118.8, 110.8. HRMS (ESI-IT-TOF) m/z: $[M+Na]^+$ Calcd for $C_{34}H_{22}NaO_4^+$ 517.1410; Found: 517.1411. $[\alpha]^{26.0}_{589}$ = +163.8 (c= 0.1, in $CHCl_3$). mp: 175.9-176.6 °C.

^{1}H NMR (400 MHz, Chloroform-*d*) spectrum of (*S*)-**3**

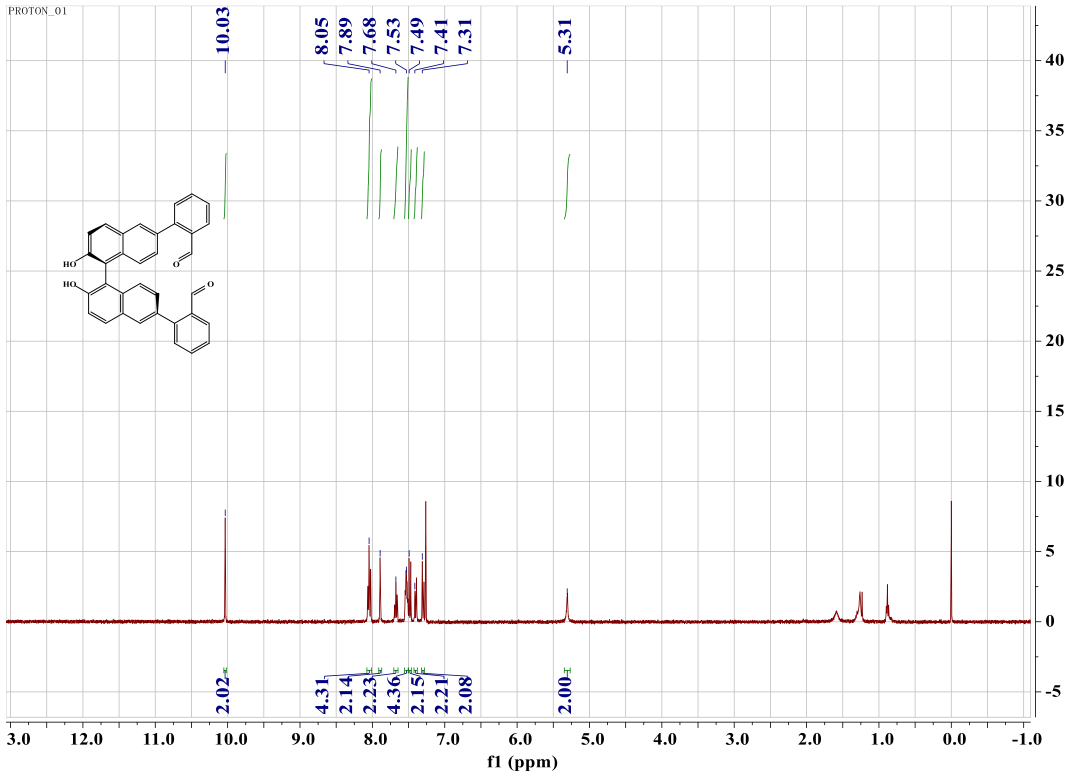

$^{13}C\{^1H\}$ NMR (100 MHz, Chloroform-*d*) spectrum of (*S*)-**3**

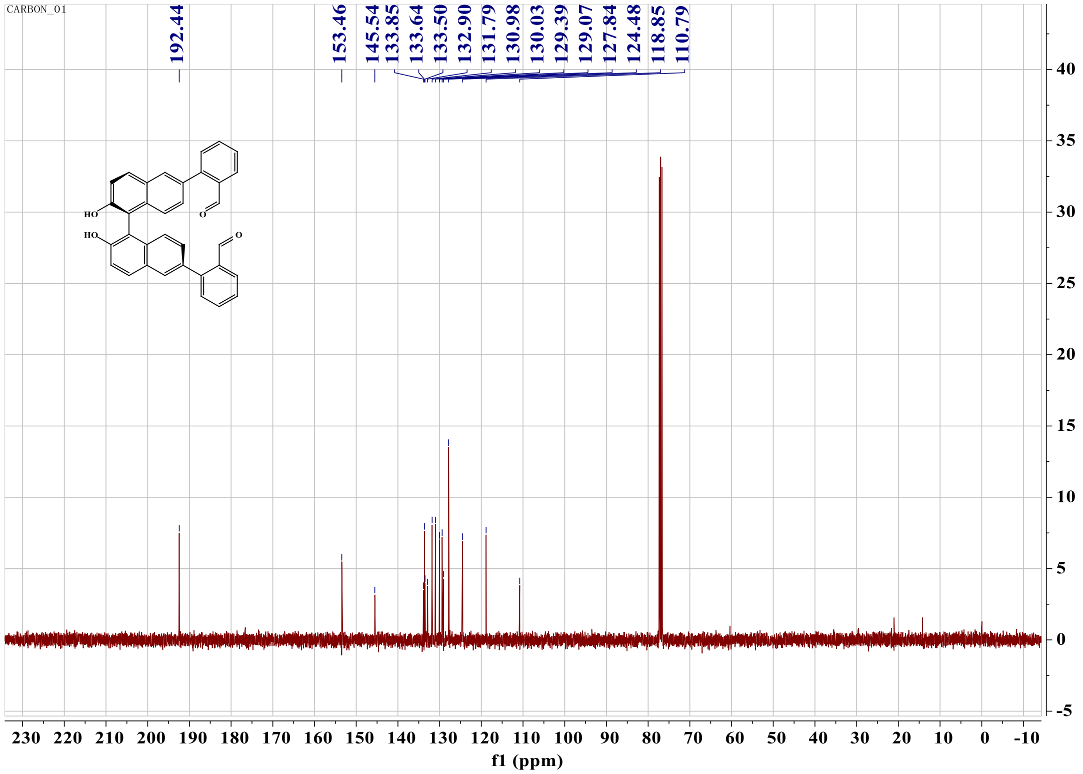

1.3.6. Synthesis and characterization of compound **4**.

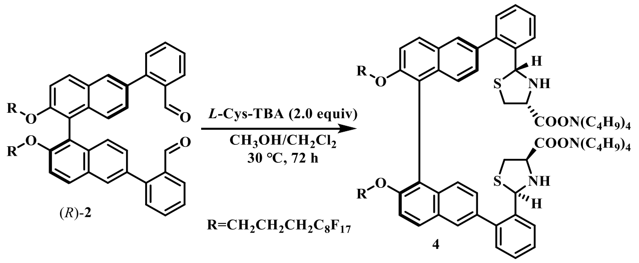

Compound **4** (0.1052 g) as a yellow solid was prepared in quantitative yield in the same way as compound **4'** by starting with tetrabutylammonium hydroxide (TBAOH, 0.1 mL, 1 M in CH_3OH, 2.0 equiv). ^1H NMR (400 MHz, Methanol-d_4) δ 8.08 (d, *J* = 9.00 Hz, 2H), 7.96 (s, 2H), 7.91 (d, *J* = 7.72 Hz, 2H), 7.60 (d, *J* = 9.28 Hz, 2H), 7.45 (m, 2H), 7.34 (m, 6H), 7.20 (d, *J* = 8.72 Hz, 2H), 5.52 (s, 2H), 4,19 (m, 2H), 4.07 (m, 2H), 3.55 (t, *J* = 7.96, 2H), 3.37 (dd, *J* = 9.88, 7.24 Hz, 2H), 3.22 (m, 16H), 3,13 (m, 2H), 1.80 (m, 8H), 1.64 (m, 16H), 1.41 (m, 16H), 1.01 (t, *J* = 7.24 Hz, 24H). $^{13}C\{^1H\}$ NMR (100 MHz, Methanol-d_4) δ 177.9, 155.6, 143.1, 137.7, 137.1, 134.4, 131.4, 131.1, 130.7, 129.5, 129.4, 129.2, 129.1, 128.4, 126.2, 121.5, 117.1, 134.8-120.0 (m), 69.8, 69.7, 69.1, 59.5, 41.5, 28.4 (t, *J* = 22.38 Hz), 24.8, 21.7, 20.7, 13.9. ^{19}F NMR (376 MHz, Methanol-d_4) δ -82.39 (t, *J* =10.19 Hz, 6F), -115.37 (m, 4F), -122.94 (m, 12F), -123.79 (m, 4F), -124.27 (m, 4F), -127.34 (m, 4F). HRMS (ESI-IT-TOF) m/z: [M-2TBA+3H]$^+$ Calcd for $C_{62}H_{43}F_{34}N_2O_6S_2^+$ 1621.2014; Found: 1621.2012. $[\alpha]^{26.0}_{589}$ = -45.6 (c= 0.1, in CH_3OH). mp: 67.3-68.4 °C.

^{1}H NMR (400 MHz, Methanol-d_4) spectrum of compound **4**

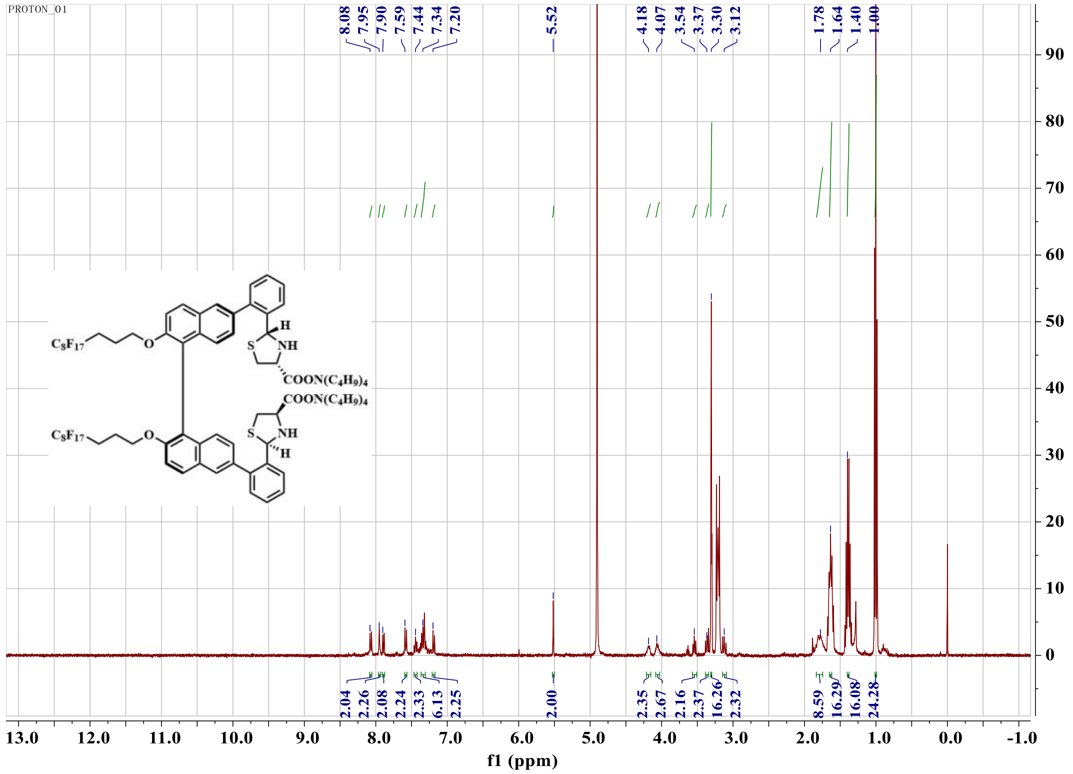

$^{13}C\{^{1}H\}$ NMR (100 MHz, Methanol-d_4) spectrum of compound **4**

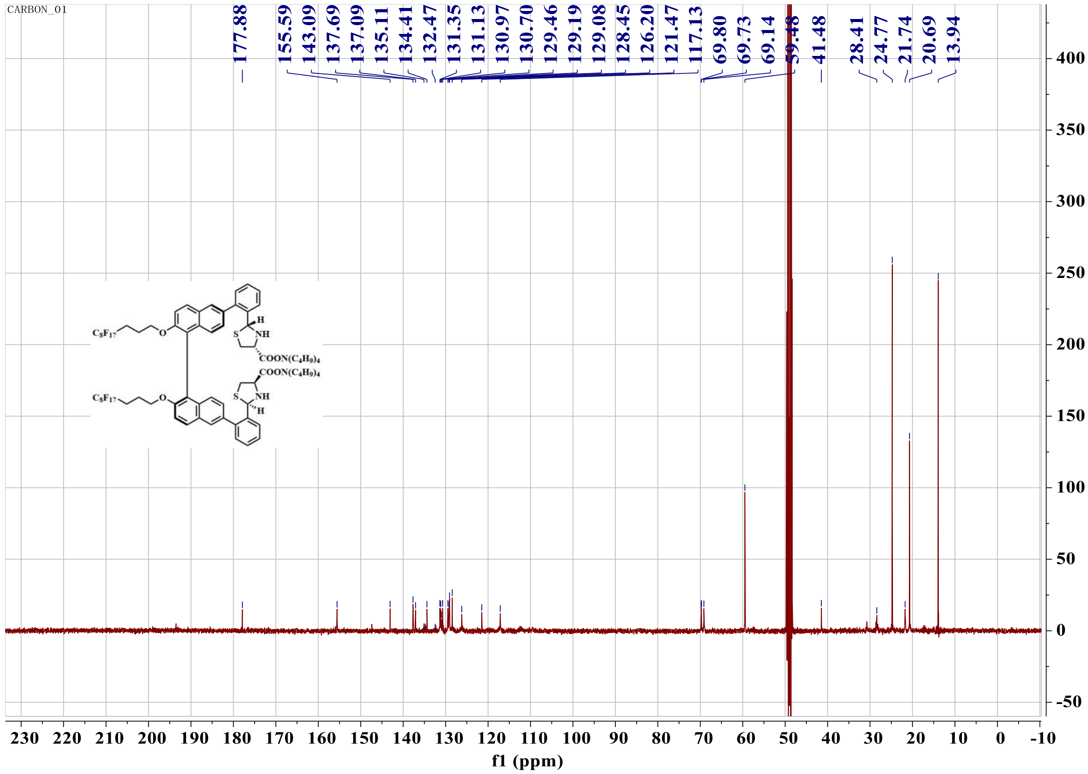

^{19}F NMR (376 MHz, Methanol-d_4) spectrum of compound **4**

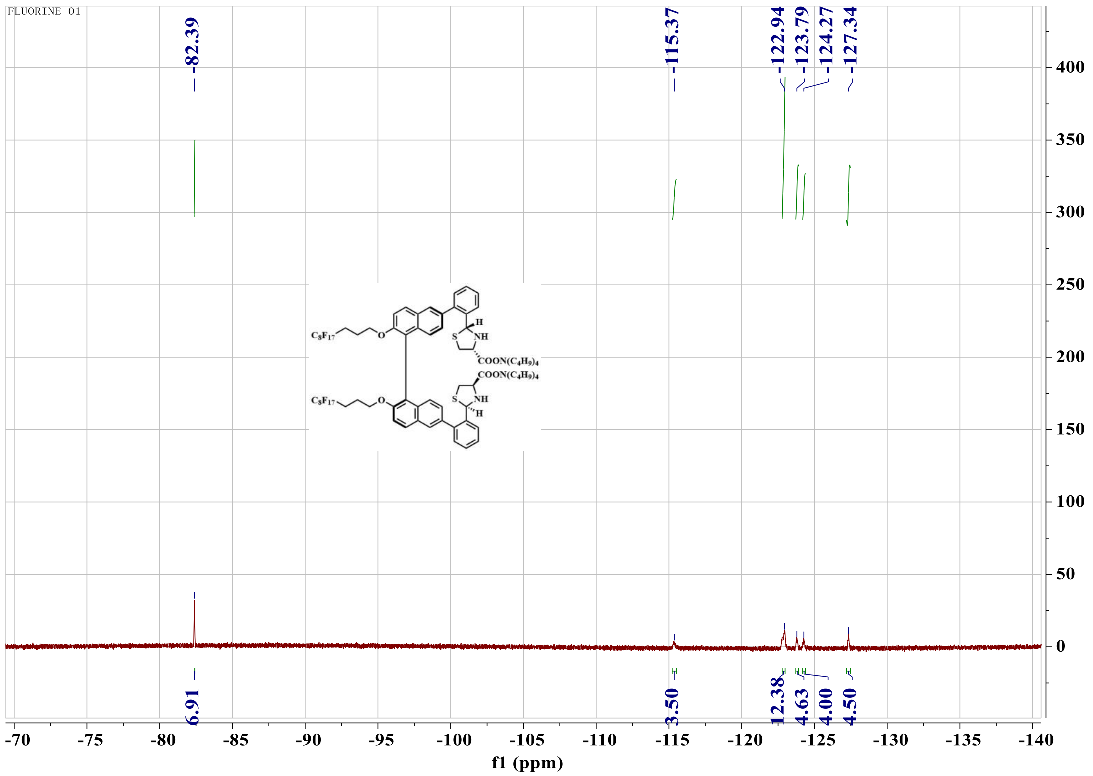

1.3.7. Synthesis and characterization of compound **4'**.

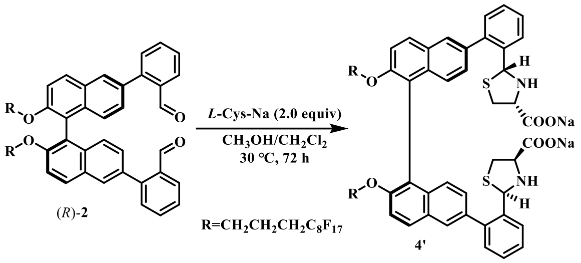

Under argon atmosphere, *L*-Cys (0.10 mmol, 0.0121 g, 2.0 equiv) and NaOH (0.1 mL, 1 M in CH_3OH, 2.0 equiv) were mixed in CH_3OH (9.5 mL) under ultrasound for 20 min to give clear and transparent methanol solution of *L*-Cys-Na. Then (*R*)-**2** (0.05 mmol, 0.0707 g, 1.0 equiv) in CH_2Cl_2 (0.5 mL) and *L*-Cys-Na in CH_3OH (9.5 mL) were stirred at 30 °C (oil bath) for 72 h. After filtration of reaction mixture, the filtrate was concentrated under reduced pressure to afford compound **4'** (0.0833 g) as a light-yellow solid in quantitative yield. ^{1}H NMR (400 MHz, Methanol-d_4) δ 8.08 (d, J = 9.20 Hz, 2H), 7.94 (s, 2H), 7.90 (d, J = 8.20 Hz, 2H), 7.59 (d, J = 9.12 Hz, 2H), 7.44 (m, 2H), 7.34 (m, 6H), 7.19 (d, J = 8.12 Hz, 2H), 5.51 (s, 2H), 4.18 (m, 2H), 4.07 (m, 2H), 3.55 (t, J = 7.36 Hz, 2H), 3.37 (m, 2H), 3.12 (m, 2H), 1.78 (m, 8H). ^{13}C{^{1}H} NMR (100 MHz, Methanol-d_4) δ 178.0, 155.6, 143.2, 137.6, 137.1, 134.4, 131.3, 131.1, 130.7, 129.4, 129.2, 129.1, 128.4, 126.2, 121.5, 117.1, 134.8-120.0 (m), 69.7, 69.6, 69.2, 41.4, 28.4 (t, J = 22.00 Hz), 21.7. ^{19}F NMR (376 MHz, Methanol-d_4) δ -82.38 (t, J = 8.84 Hz, 6F), -115.37 (m, 4F), -122.93 (m, 12F), -123.79 (m, 4F), -124.28 (m, 4F), -127.34 (m, 4F). HRMS (ESI-IT-TOF) m/z: [M-2Na+3H]$^+$ Calcd for $C_{62}H_{43}F_{34}N_2O_6S_2^+$ 1621.2014; Found: 1621.2042. [M-2Na+H]$^-$ Calcd for $C_{62}H_{41}F_{34}N_2O_6S_2^-$ 1619.1869; Found: 1619.1703. $[\alpha]^{26.0}_{589}$ = -59.4 (c= 0.1, in CH_3OH). mp: decomposed over 300 °C.

^{1}H NMR (400 MHz, Methanol-d_4) spectrum of compound **4’**

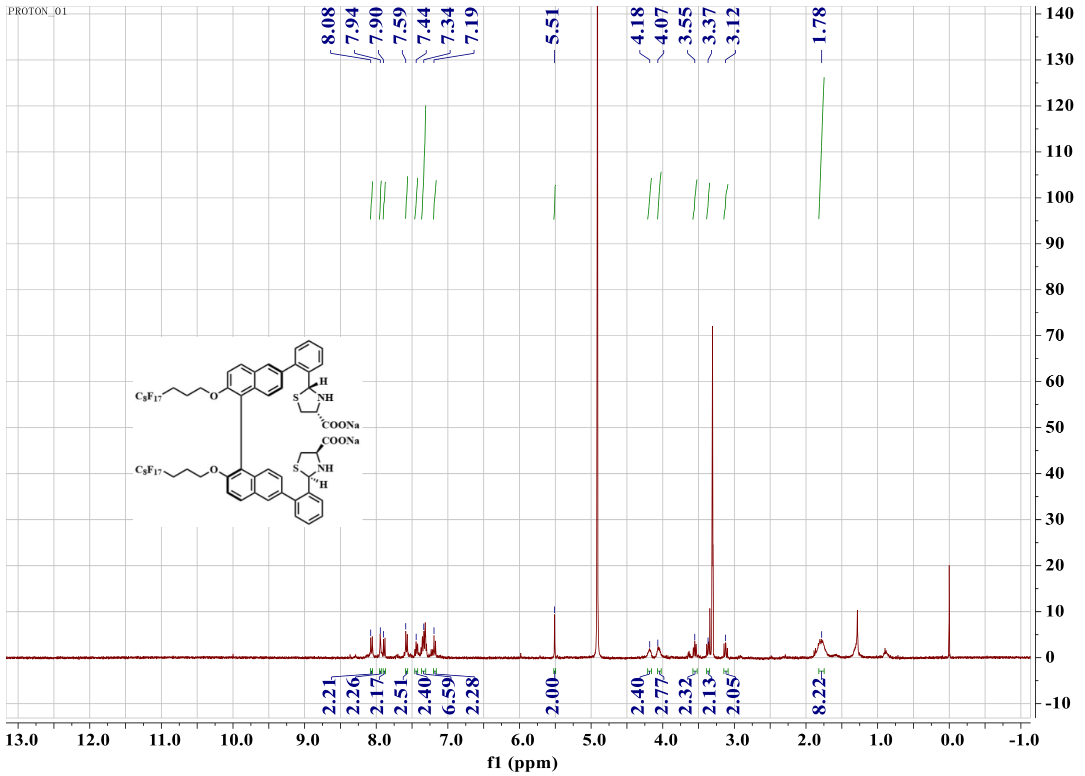

$^{13}C\{^{1}H\}$ NMR (100 MHz, Methanol-d_4) spectrum of compound **4’**

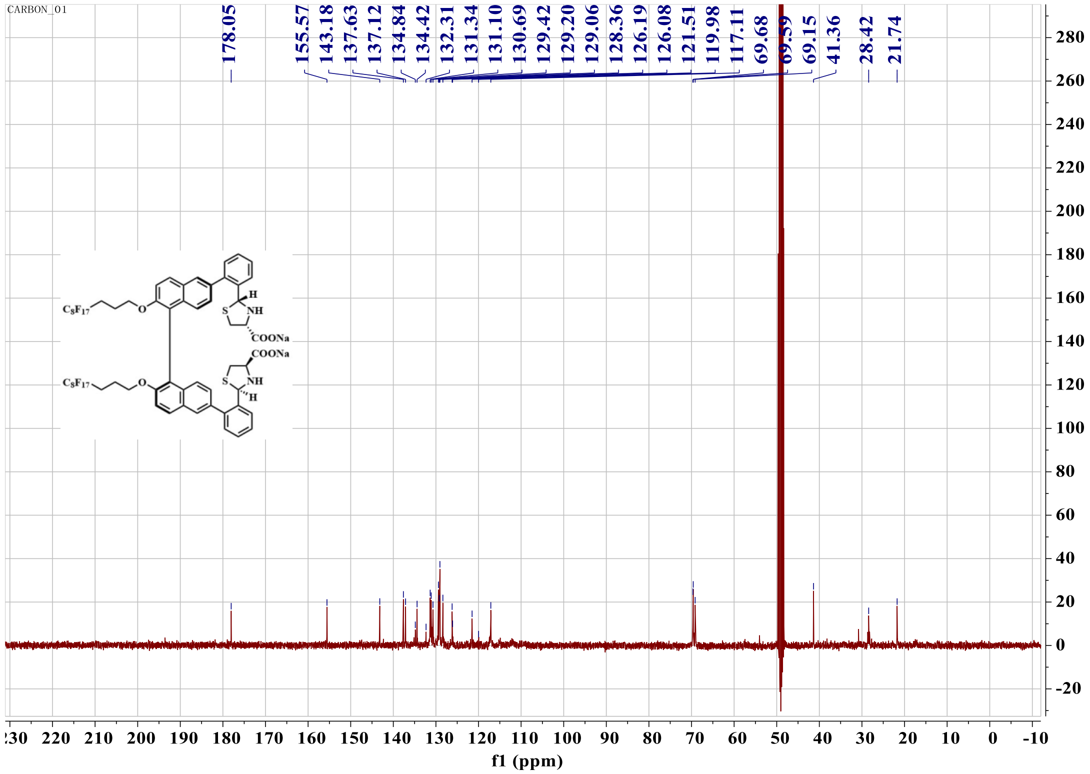

^{19}F NMR (376 MHz, Methanol-d_4) spectrum of compound **4’**

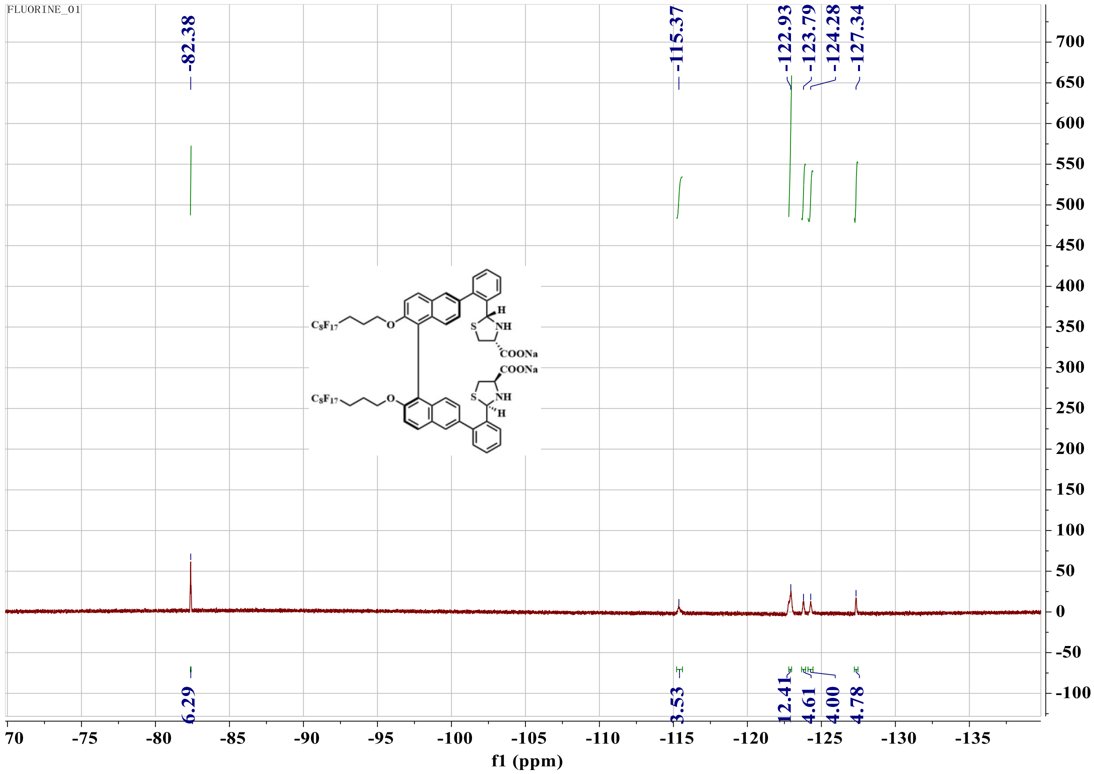

1.3.8. Synthesis and characterization of compound **5**.

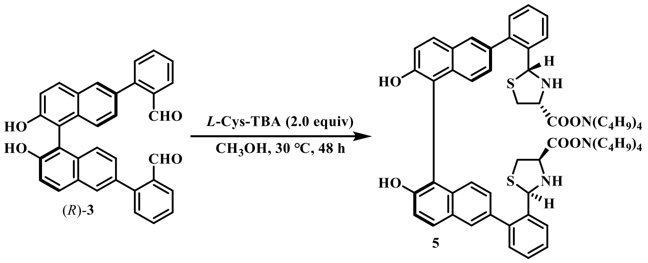

Compound **5** (0.1184 g) as a yellow solid was prepared in quantitative yield in the same way as compound **5’** by starting with tetrabutylammonium hydroxide (TBAOH, 0.2 mL, 1 M in CH_3OH, 2.0 equiv). ^{1}H NMR (400 MHz, Methanol-d_4) δ 7.94 (d, J = 9.20 Hz, 2H), 7.89 (m, 4H), 7.44 (dd, J = 8.80, 7.24 Hz, 2H), 7.35 (m, 6H), 7.26 (d, J = 8.68 Hz, 2H), 7.17 (d, J = 8.80 Hz, 2H), 5.51 (s, 1H), 3.53 (dd, J =8.52, 7.12 Hz, 2H), 3.37 (dd, J =10.08, 7.12 Hz, 2H), 3.20 (m, 16H), 3.12 (dd, J =10.08, 8.52 Hz, 2H), 1.63 (m, 16H), 1.40 (m, 16H), 1.01 (t, J = 7.32 Hz, 24H). ^{13}C{^{1}H} NMR (100 MHz, Methanol-d_4) δ 177.8, 154.9, 143.3, 137.7, 135.9, 135.0, 131.4, 130.9, 130.0, 129.5, 129.1, 129.0, 128.8, 128.5, 125.9, 120.1, 116.2, 69.9, 69.8, 59.4, 41.5, 24.7, 20.7, 14.0. HRMS (ESI-IT-TOF) m/z: [M-TBA]$^-$ Calcd for $C_{56}H_{66}N_3O_6S_2^-$ 940.4399; Found: 940.4334. [M-H]$^-$ Calcd for $C_{72}H_{101}N_4O_6S_2^-$ 1181.7168; Found: 1181.7144. $[\alpha]^{26.0}_{589}$ = -138.2 (c= 0.1, in CH_3OH). mp: 152.8-153.5 °C.

^{1}H NMR (400 MHz, Methanol-*d*$_4$) spectrum of compound **5**

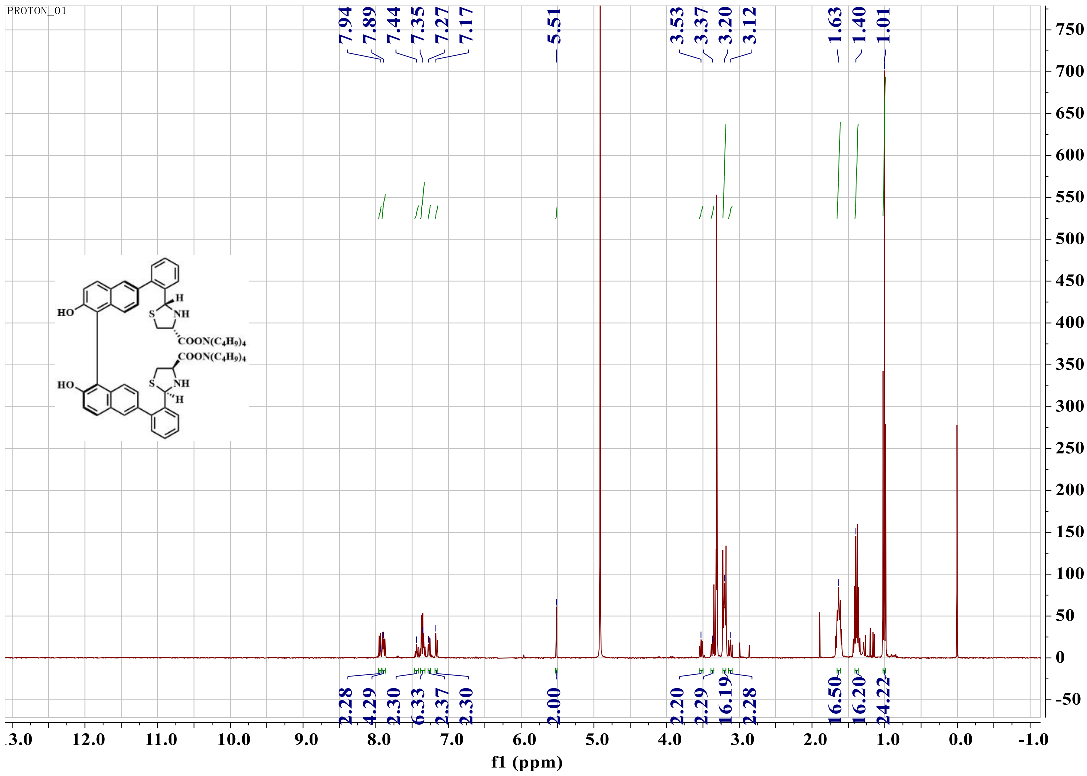

^{13}C{^{1}H} NMR (100 MHz, Methanol-*d*$_4$) spectrum of compound **5**

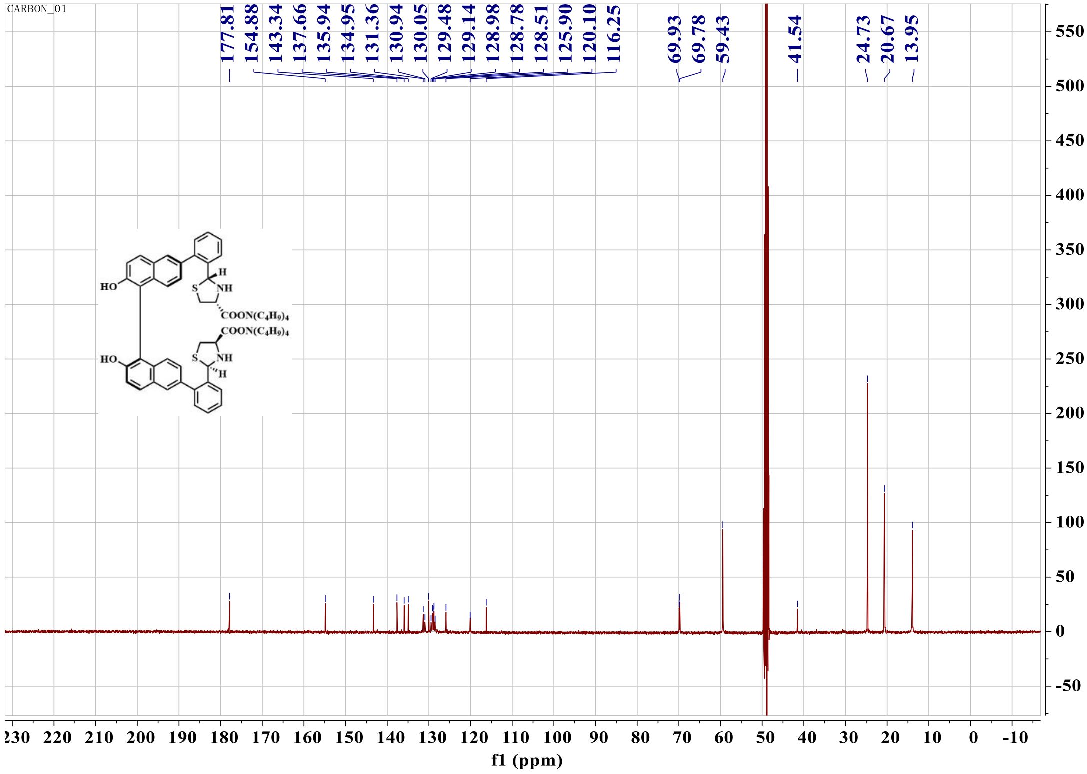

1.3.9. Synthesis and characterization of compound **5'**.

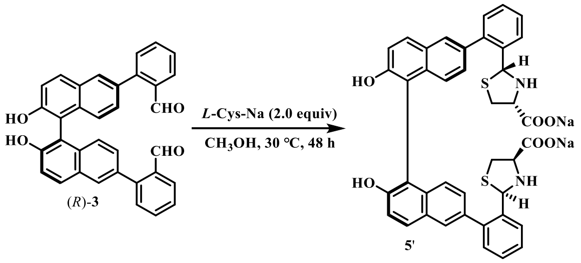

Under argon atmosphere, *L*-Cys (0.20 mmol, 0.0242 g, 2.0 equiv) and NaOH (0.2 mL, 1 M in CH_3OH, 2.0 equiv) were mixed in CH_3OH (5 mL) under ultrasound for 20 min to give clear and transparent methanol solution of *L*-Cys-Na. Then (*R*)-**3** (0.10 mmol, 0.0500 g, 1.0 equiv) in CH_3OH (5 mL) and *L*-Cys-Na in CH_3OH (5 mL) were stirred at 30 °C (oil bath) for 48 h. After filtration of reaction mixture, the filtrate was concentrated under reduced pressure to afford compound **5'** (0.0745 g) as a light-yellow solid in quantitative yield. ^{1}H NMR (400 MHz, Methanol-*d4*) δ 7.95 (d, *J* = 9.12 Hz, 2H), 7.89 (m, 4H), 7.44 (dd, *J* = 9.12, 7.24 Hz, 2H), 7.35 (m, 6H), 7.26 (d, *J* = 8.72 Hz, 2H), 7.17 (d, *J* = 8.72 Hz, 2H), 5.51 (s, 1H), 3.53 (dd, *J* =8.52, 7.24 Hz, 2H), 3.37 (dd, *J* =10.16, 7.32 Hz, 2H), 3.12 (dd, *J* =10.20, 8.52 Hz, 2H). ^{13}C{^{1}H} NMR (100 MHz, Methanol-*d4*) δ 178.0, 154.7, 143.5, 137.6, 136.0, 135.0, 131.4, 130.9, 129.4, 129.1, 128.9, 128.8, 128.3, 125.8, 120.0, 116.1, 69.7, 69.6, 41.4. HRMS (ESI-IT-TOF) m/z: $[M+H]^+$ Calcd for $C_{40}H_{31}N_2Na_2O_6S_2^+$ 745.1413; Found: 745.1415. $[M-Na+2H]^+$ Calcd for $C_{40}H_{32}N_2NaO_6S_2^+$ 723.1594; Found: 723.1595. $[M-2Na+3H]^+$ Calcd for $C_{40}H_{33}N_2O_6S_2^+$ 701.1775; Found: 701.1767. $[\alpha]^{26.0}_{589}$ = -203.2 (c= 0.1, in CH_3OH). mp: decomposed over 300 °C.

^{1}H NMR (400 MHz, Methanol-*d4*) spectrum of compound **5'**

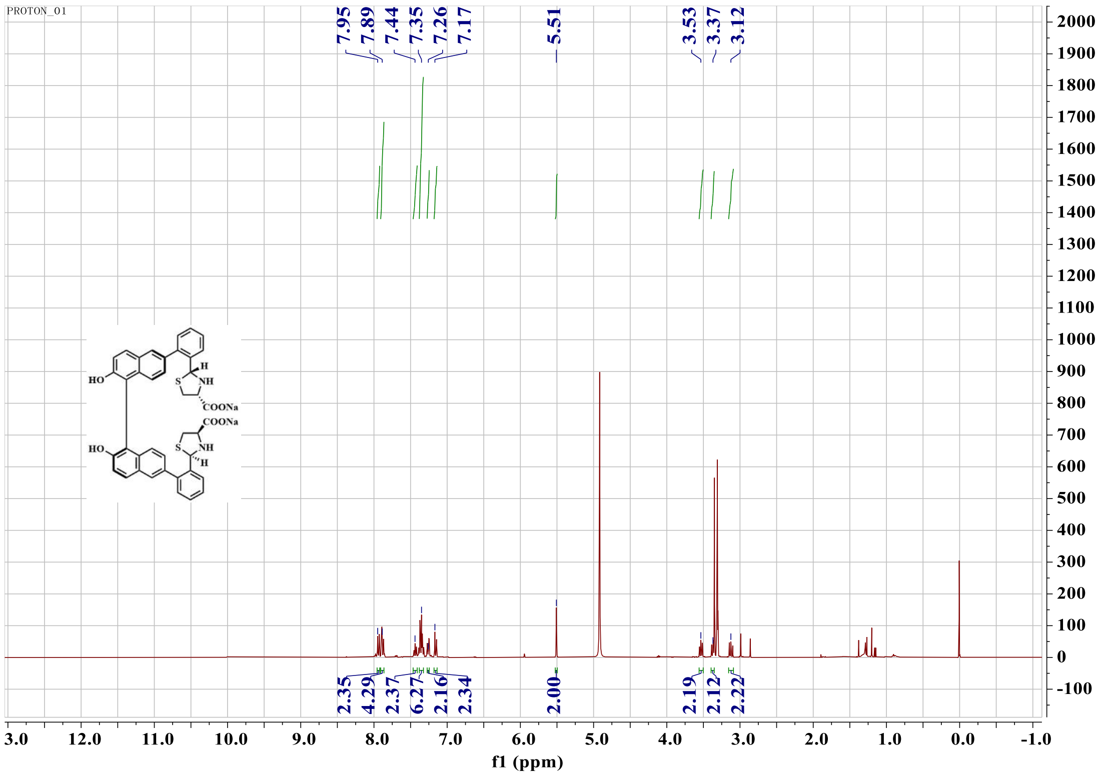

$^{13}C\{^1H\}$ NMR (100 MHz, Methanol-d_4) spectrum of **compound 5'**

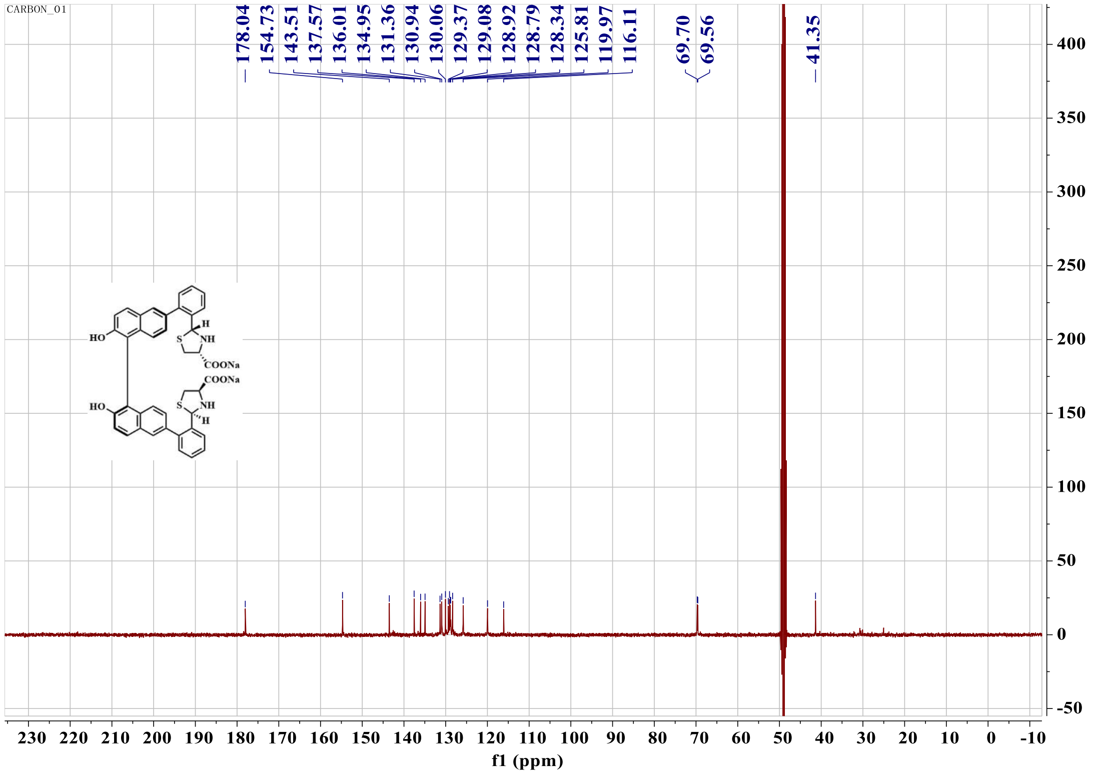

1.3.10. Synthesis and characterization of (*R*)-**7**.

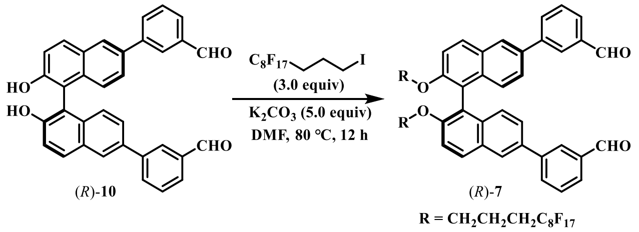

(*R*)-**7** (0.5030 g) was purified by flash column chromatography on silica gel (eluted with petroleum ether/ethyl acetate, 10/1, v/v) to afford a yellow amorphous solid in 61% yield in the same way as (*R*)-**2** by starting with (*R*)-**10** (0.58 mmol, 0.2860 g, 1.0 equiv). ^1H NMR (400 MHz, Chloroform-*d*) δ 10.10 (s, 2H), 8.19 (s, 2H), 8.15 (s, 2H), 8.05 (d, *J* = 9.00 Hz, 2H), 7.94 (d, *J* = 7.72 Hz, 2H), 7.86 (d, *J* = 7.64 Hz, 2H), 7.62 (t, *J* = 7.72 Hz, 2H), 7.56 (d, *J* = 8.80 Hz, 2H), 7.47 (d, *J* = 9.08 Hz, 2H), 7.31 (d, *J* = 8.80 Hz, 2H), 4.13 (m, 2H), 3.99 (m, 2H), 1.72 (m, 4H), 1.61 (m, 4H). $^{13}C\{^1H\}$ NMR (100 MHz, Chloroform-*d*) δ 192.2, 154.2, 141.9, 137.0, 135.2, 133.4, 133.0, 130.1, 129.7, 129.5, 128.5, 128.1, 126.3, 126.0, 125.8, 120.3, 116.1, 118.52-107.16 (m), 68.2, 27.2 (t, *J* = 22.39 Hz), 20.6. ^{19}F NMR (376 MHz, Chloroform-*d*) δ -80.77 (t, *J* =9.55 Hz, 6F), -114.58 (m, 4F), -121.94 (m, 12F), -122.73 (m, 4F), -123.46 (m, 4F), -126.10 (m, 4F). HRMS (ESI-IT-TOF) m/z: $[M+Na]^+$ Calcd for $C_{56}H_{32}F_{34}NaO_4^+$ 1437.1650; Found: 1437.1715. $[\alpha]^{26.0}_{589}$ = -22.8 (c= 0.1, in $CHCl_3$). mp: 65.1-65.6 °C.

^{1}H NMR (400 MHz, Chloroform-*d*) spectrum of (*R*)-**7**

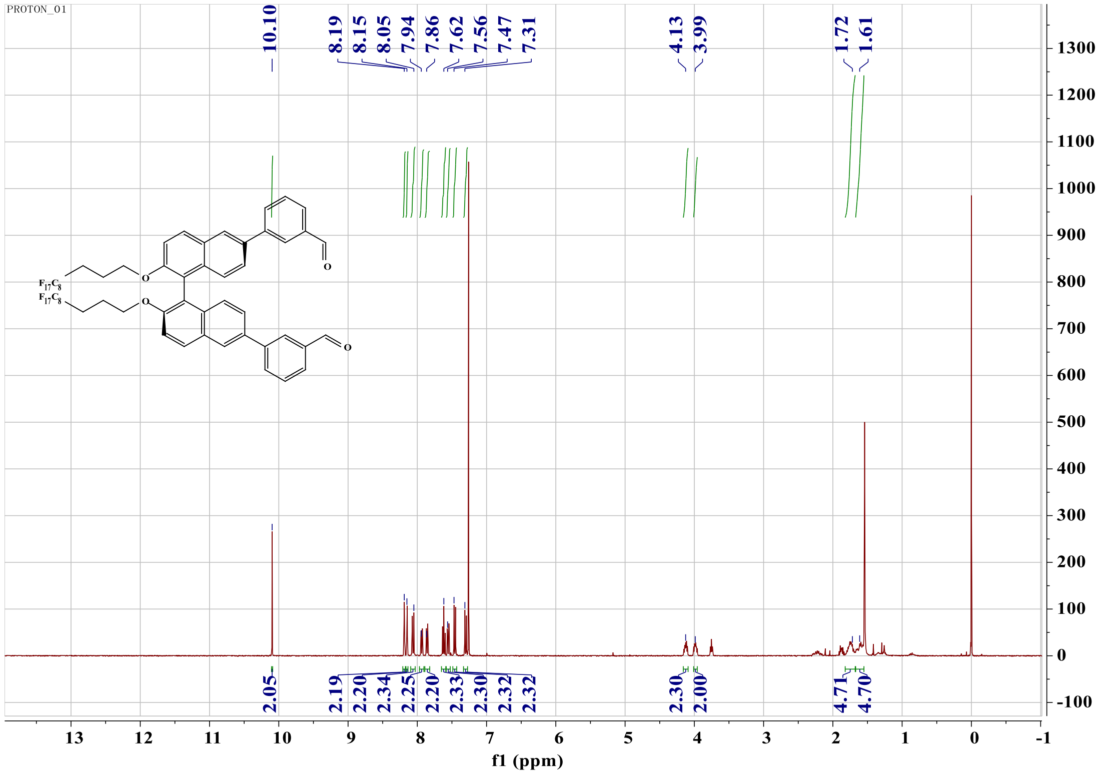

^{13}C{^{1}H} NMR (100 MHz, Chloroform-*d*) spectrum of (*R*)-**7**

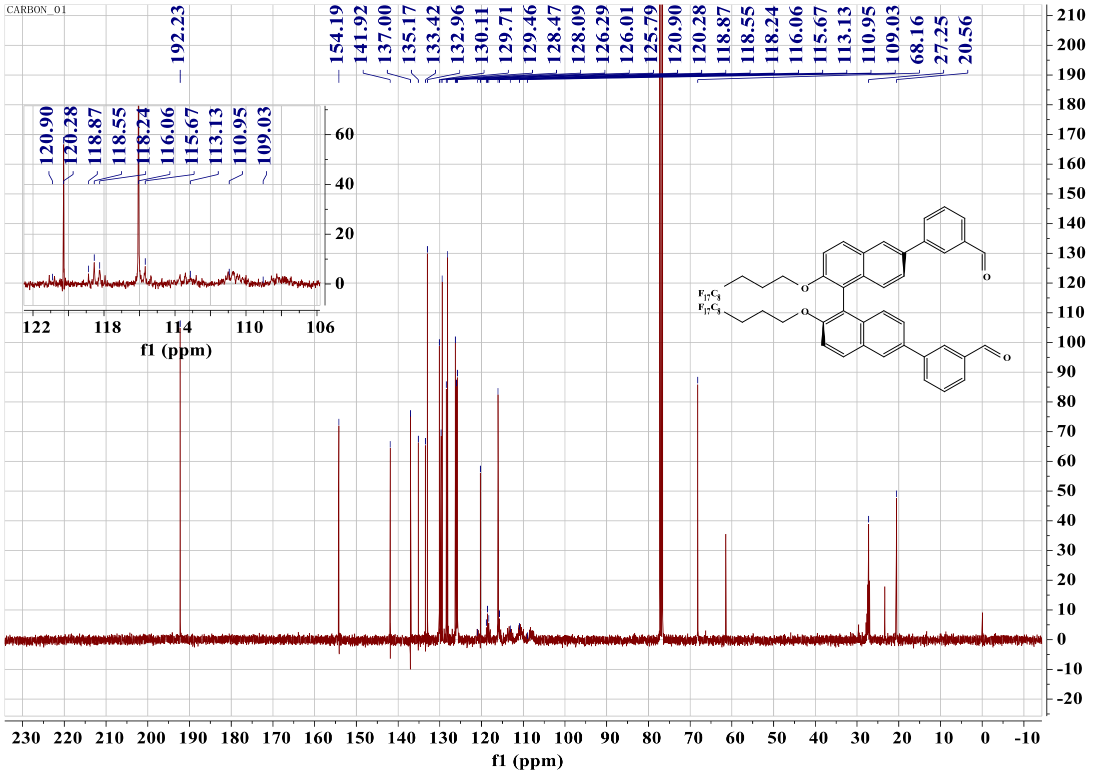

^{19}F NMR (376 MHz, Chloroform-*d*) spectrum of (*R*)-**7**

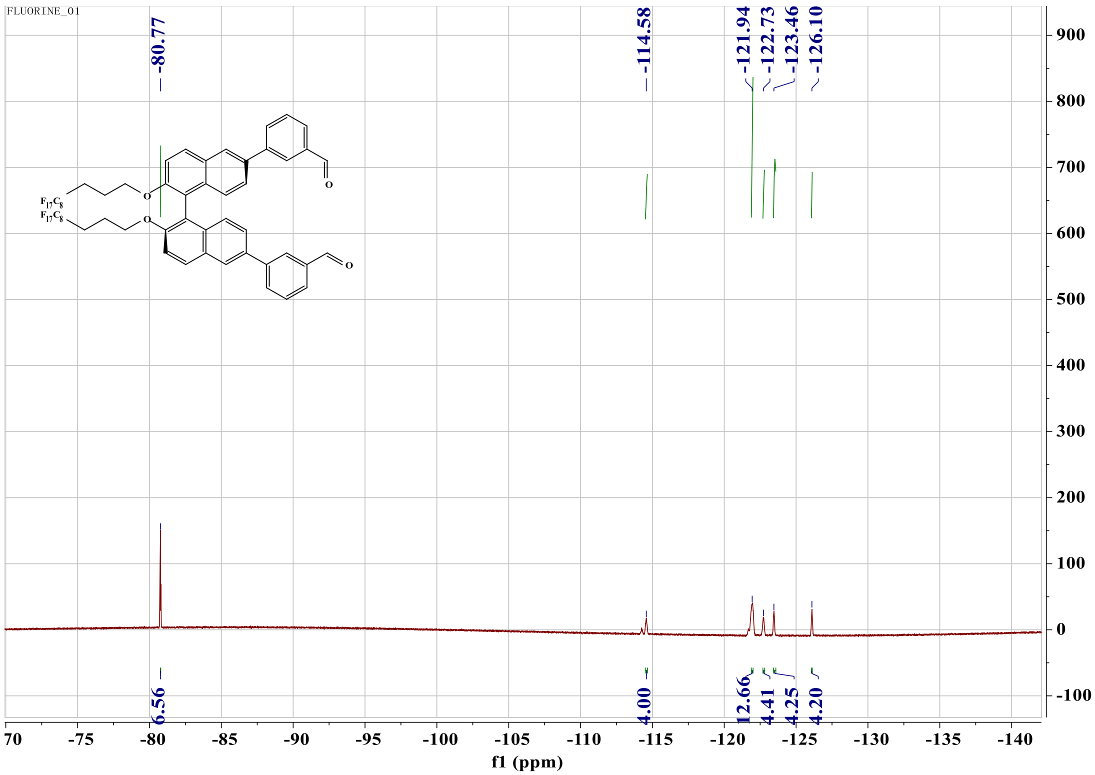

1.3.11. Synthesis and characterization of (*R*)-**8**.

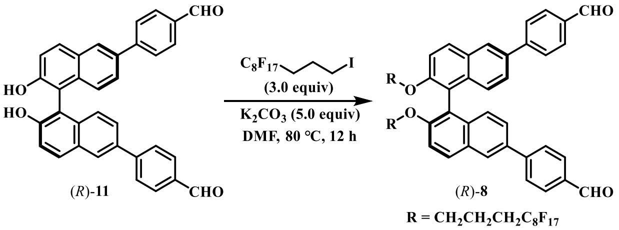

(*R*)-**8** (0.3500 g) was purified by flash column chromatography on silica gel (eluted with petroleum ether/ethyl acetate, 10/1, v/v) to afford a yellow amorphous solid in 62% yield in the same way as (*R*)-**2** by starting with (*R*)-**11** (0.40 mmol, 0.1970 g, 1.0 equiv). ^{1}H NMR (400 MHz, Chloroform-*d*) δ 10.06 (s, 2H), 8.16 (s, 2H), 8.07 (d, *J* = 8.92 Hz, 2H), 7.97 (d, *J* = 8.60 Hz, 4H), 7.84 (d, *J* = 8.20 Hz, 4H), 7.56 (d, *J* = 8.80 Hz, 2H), 7.47 (d, *J* = 9.00 Hz, 2H), 7.31 (d, *J* = 8.80 Hz, 2H), 4.13 (m, 2H), 3.99 (m, 2H), 1.76 (m, 4H), 1.61 (m, 4H). ^{13}C{^{1}H} NMR (100 MHz, Chloroform-*d*) δ 191.8, 154.4, 146.9, 135.1, 135.1, 133.6, 130.3, 130.3, 129.6, 127.6, 126.8, 126.0, 125.8, 120.1, 116.0, 121.1-108.2 (m), 68.1, 27.2 (t, *J* = 22.00 Hz), 20.5. ^{19}F NMR (376 MHz, Chloroform-*d*) δ -80.76 (t, *J* =10.23 Hz, 6F), -114.59 (m, 4F), -121.96 (m, 12F), -122.74 (m, 4F), -123.47 (m, 4F), -126.11 (m, 4F). HRMS (ESI-IT-TOF) m/z: [M+Na]$^+$ Calcd for $C_{56}H_{32}F_{34}NaO_4^+$ 1437.1650; Found: 1437.1720. $[\alpha]^{26.0}_{589}$ = -55.2 (c= 0.1, in CHCl$_3$). mp: 76.4-77.2 °C.

^{1}H NMR (400 MHz, Chloroform-*d*) spectrum of (*R*)-**8**

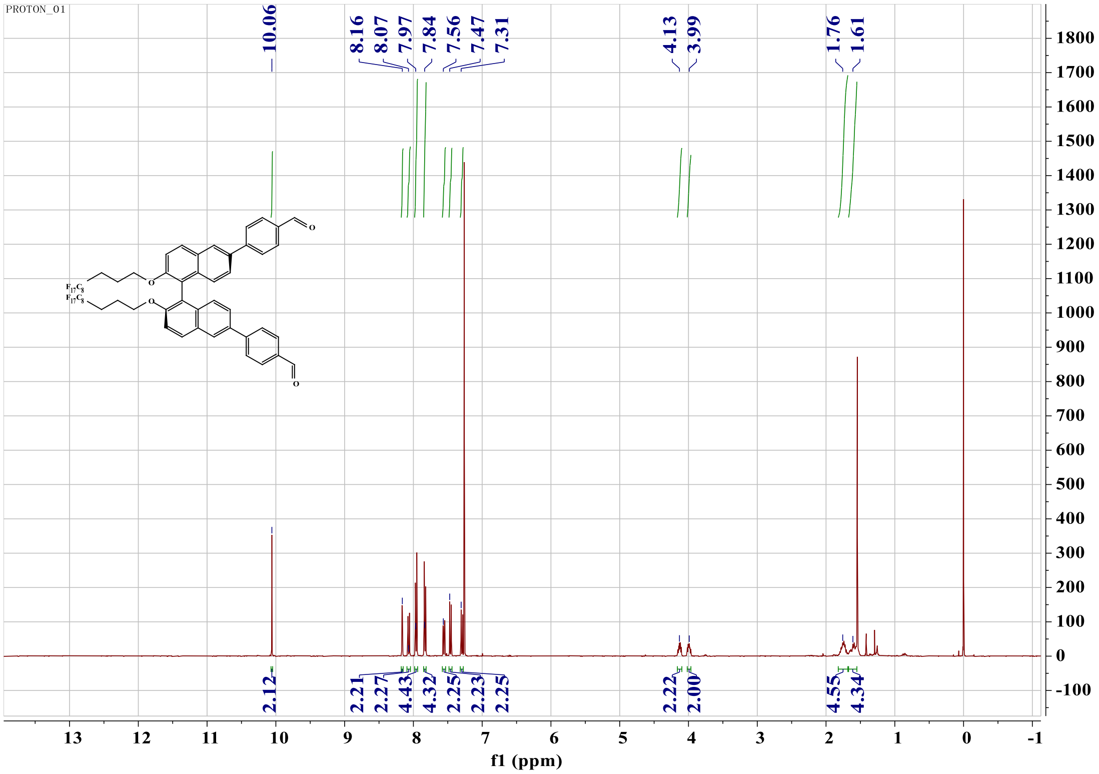

$^{13}C\{^{1}H\}$ NMR (100 MHz, Chloroform-*d*) spectrum of (*R*)-**8**

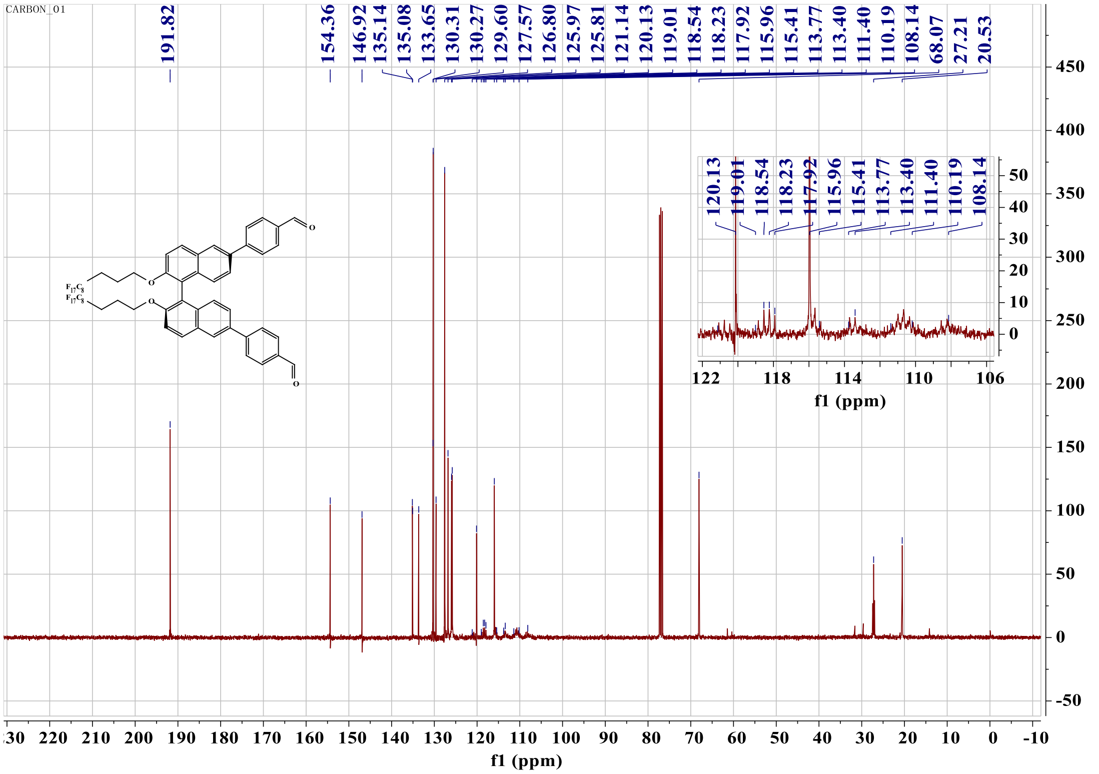

^{19}F NMR (376 MHz, Chloroform-*d*) spectrum of (*R*)-**8**

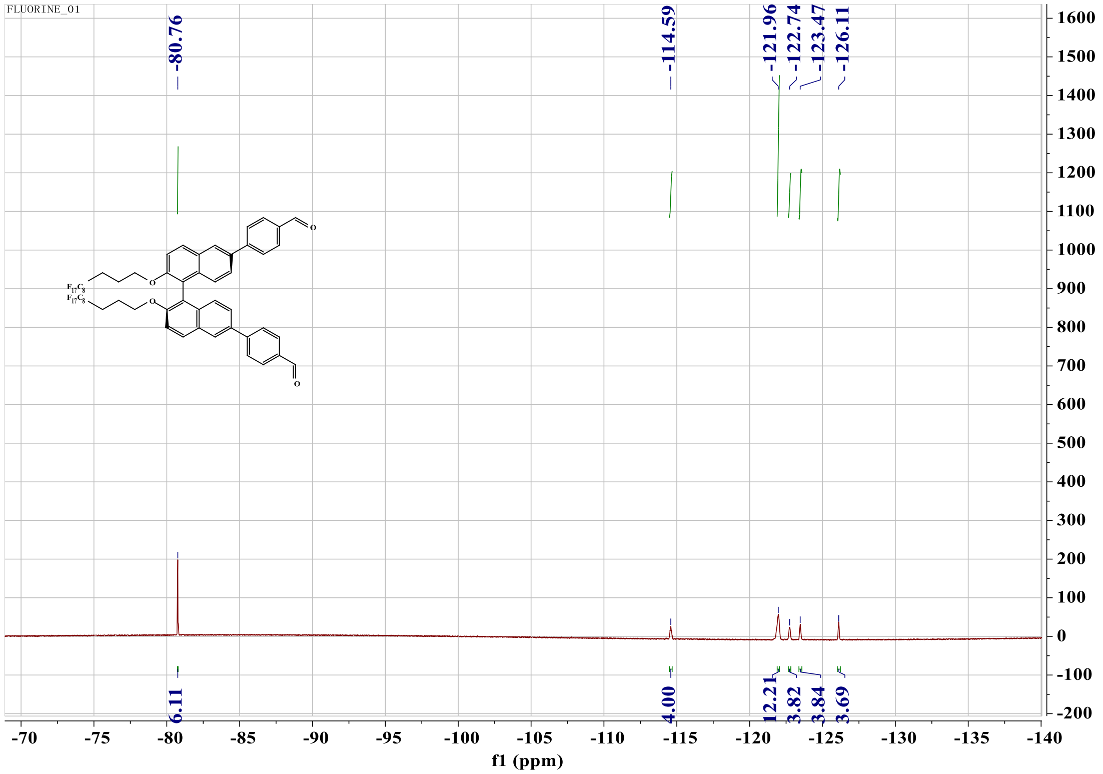

1.3.12. Synthesis and characterization of (*R*)-**10**.

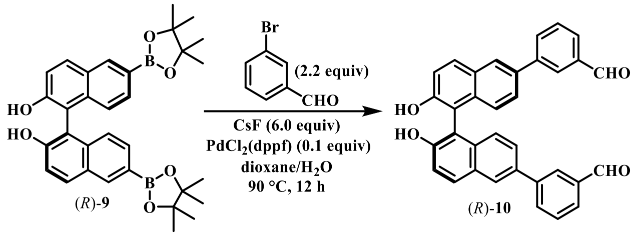

(*R*)-**10** (0.2860 g) was purified by flash column chromatography on silica gel (eluted with petroleum ether/ethyl acetate, 10/1, v/v) to afford a faint yellow amorphous solid in 21% yield in the same way as (*R*)-**3** by starting with (*R*)-**9** (2.8 mmol, 1.5000 g, 1.0 equiv) and 3-bromobenzaldehyde (6.2 mmol, 1.1471 g, 2.2 equiv). ^{1}H NMR (400 MHz, Chloroform-*d*) δ 10.08 (s, 2H), 8.18 (s, 2H), 8.16 (s, 2H), 8.09 (d, *J* = 9.32 Hz, 2H), 7.95 (d, *J* = 7.72 Hz, 2H), 7.87 (d, *J* = 7.64 Hz, 2H), 7.63 (m, 4H), 7.48 (d, *J* = 8.88 Hz, 2H), 7.30 (d, *J* = 8.72 Hz, 2H), 5.24 (s, 2H). ^{13}C{^{1}H} NMR (100 MHz, Chloroform-*d*) δ 192.4, 153.3, 141.7, 136.9, 135.2, 133.1, 133.0, 131.8, 129.6, 128.8, 128.1, 126.7, 125.1, 118.7, 110.9. HRMS (ESI-IT-TOF) m/z: [M+Na]$^{+}$ Calcd for $C_{34}H_{22}NaO_4^{+}$ 517.1410; Found: 517.1411. $[\alpha]^{26.0}_{589}$ = -238.8 (c= 0.1, in $CHCl_3$). mp: 167.6-168.7 °C.

^{1}H NMR (400 MHz, Chloroform-*d*) spectrum of (*R*)-**10**

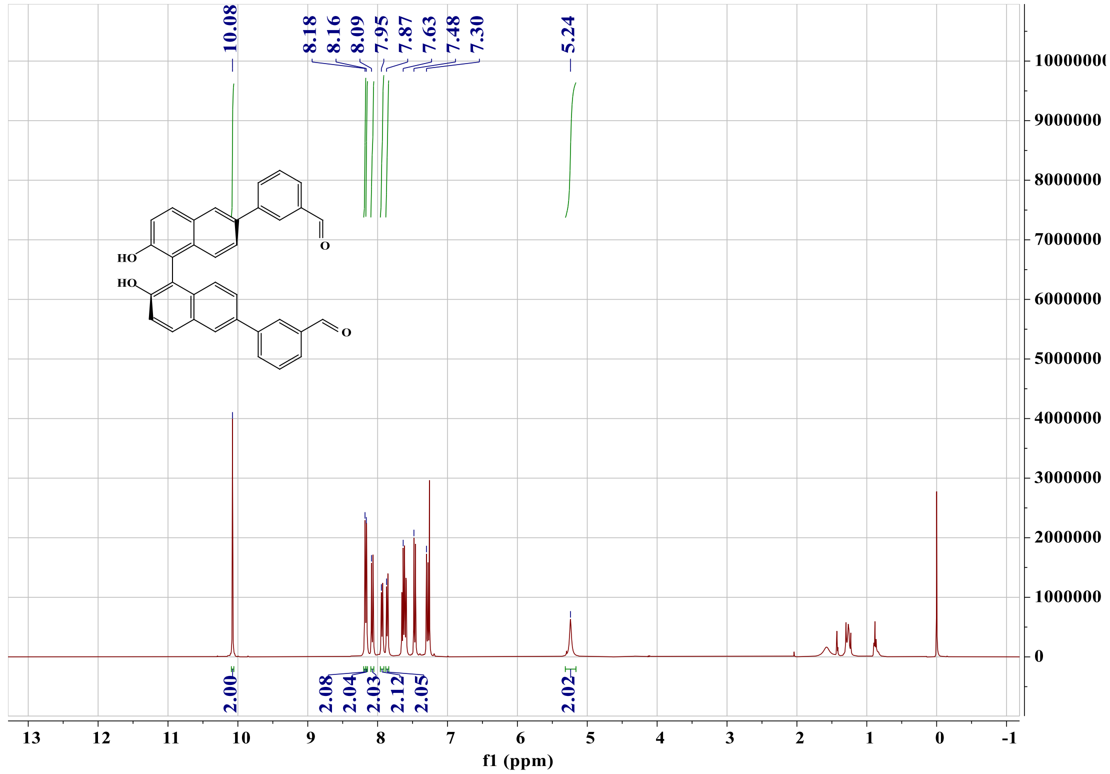

^{13}C{^{1}H} NMR (100 MHz, Chloroform-*d*) spectrum of (*R*)-**10**

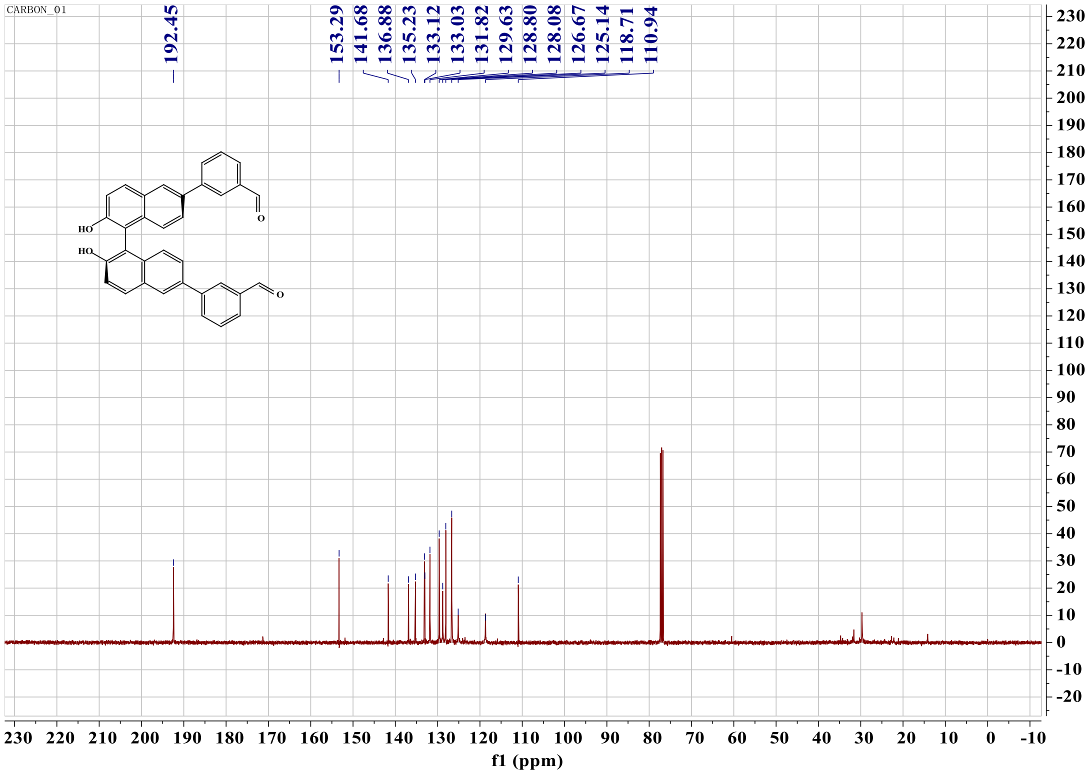

1.3.13. Synthesis and characterization of (*R*)-**11**.

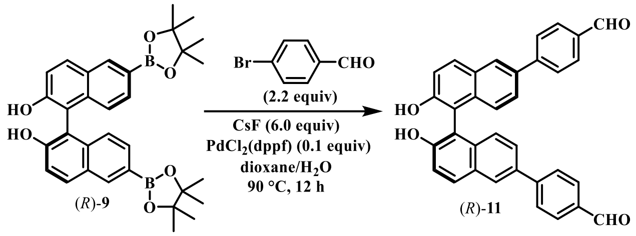

(*R*)-**11** (0.1970 g) was purified by flash column chromatography on silica gel (eluted with petroleum ether/ethyl acetate, 10/1, v/v) to afford a faint yellow amorphous solid in 14% yield in the same way as (*R*)-**3** by starting with (*R*)-**9** (2.8 mmol, 1.5000 g, 1.0 equiv) and 4-bromobenzaldehyde (6.2 mmol, 1.1471 g, 2.2 equiv). ^{1}H NMR (400 MHz, Chloroform-*d*) δ 10.03 (s, 2H), 8.16 (s, 2H), 8.10 (d, *J* = 8.40 Hz, 2H), 7.97 (d, *J* = 8.60 Hz, 4H), 7.84 (d, *J* = 8.40 Hz, 4H), 7.62 (d, *J* = 8.72 Hz, 2H), 7.49 (d, *J* = 9.00 Hz, 2H), 7.30 (d, *J* = 8.72 Hz, 2H), 5.29 (s, 2H). ^{13}C{^{1}H} NMR (100 MHz, Chloroform-*d*) δ 192.0, 153.6, 146.7, 135.2, 135.0, 133.3, 132.0, 130.4, 129.6, 127.6, 127.1, 126.6, 125.1, 118.8, 111.0. HRMS (ESI-IT-TOF) m/z: $[M+Na]^+$ Calcd for $C_{34}H_{22}NaO_4^+$ 517.1410; Found: 517.1409. $[\alpha]^{26.0}_{589}$ = -344.6 (c= 0.1, in $CHCl_3$). mp: 154.9-155.7 °C.

^{1}H NMR (400 MHz, Chloroform-*d*) spectrum of (*R*)-**11**

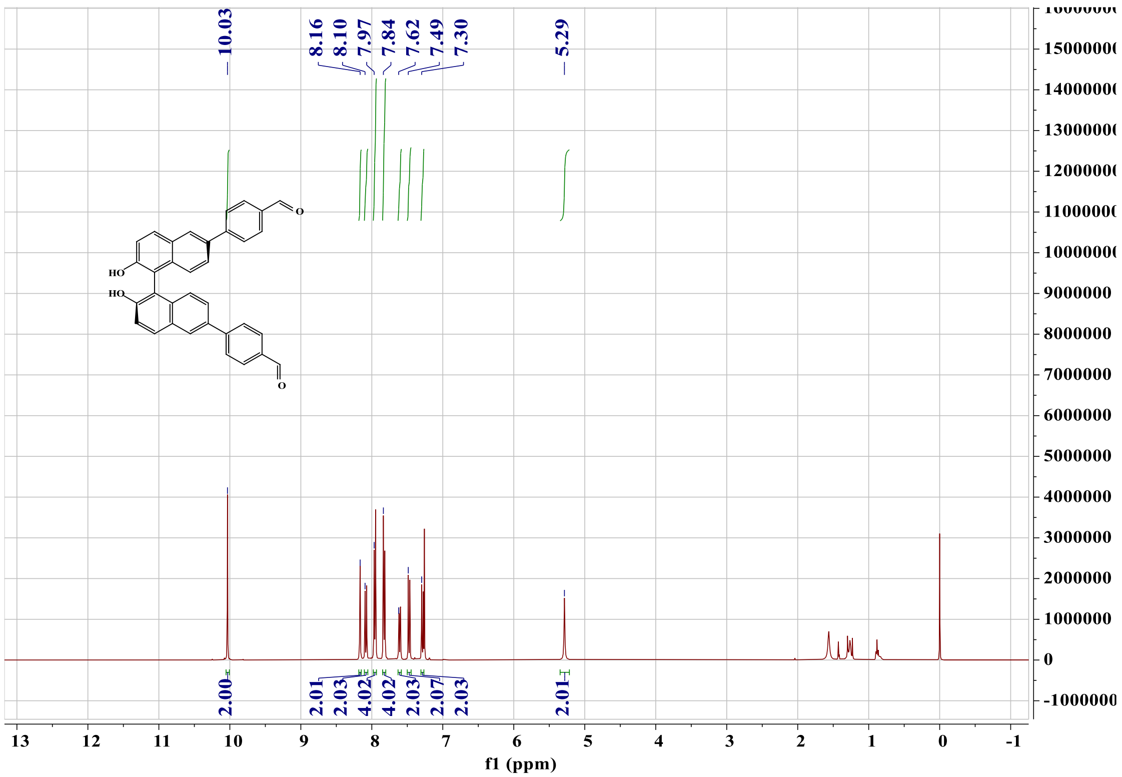

$^{13}C\{^{1}H\}$ NMR (100 MHz, Chloroform-*d*) spectrum of (*R*)-**11**

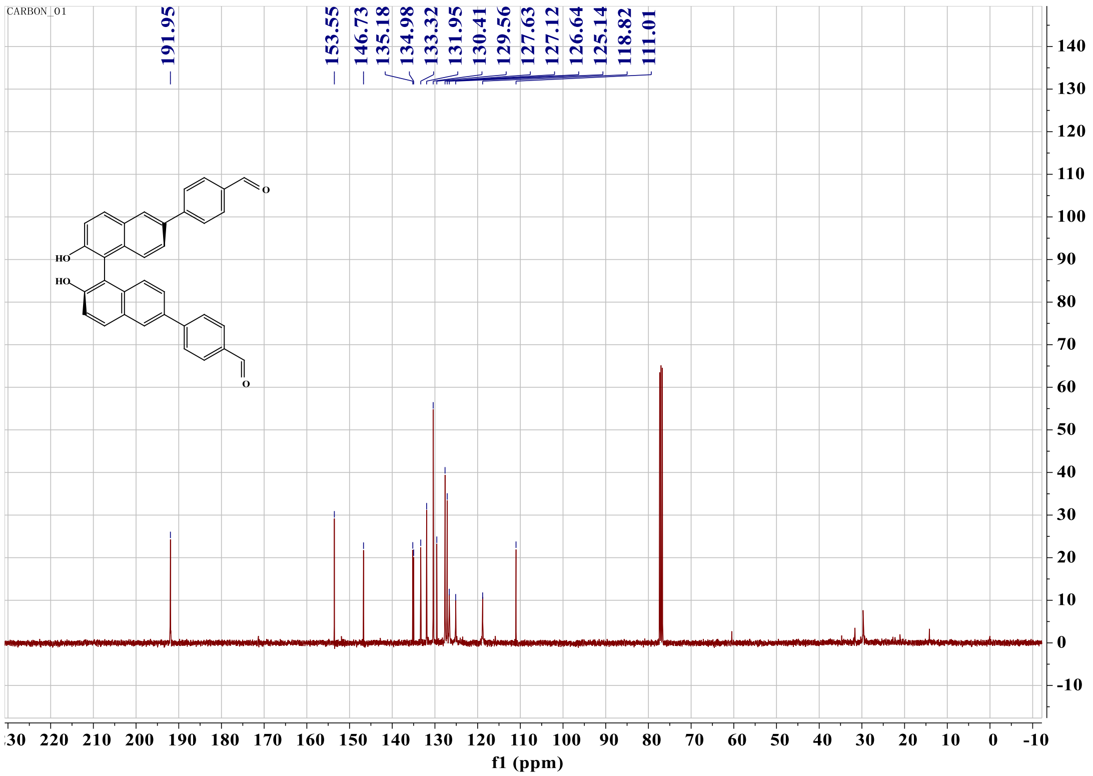

1.3.14. Synthesis and characterization of compound **12**.

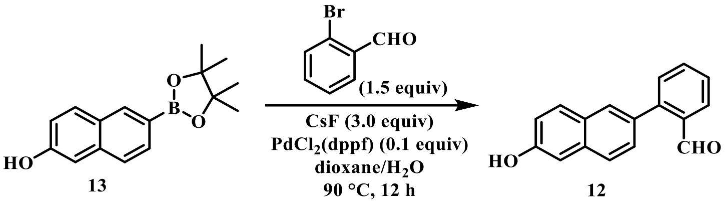

Under argon atmosphere, compound **13** (4.0 mmol, 1.0806 g, 1.0 equiv), 2-bromobenzaldehyde (6.0 mmol, 1.1101 g, 1.5 equiv), Pd(dppf)Cl_2 (0.4 mmol, 0.2927 g, 0.1 equiv) and CsF (12.0 mmol, 1.8228 g, 3.0 equiv) were dissolved in deoxidized dioxane/water (120 mL, 2/1, v/v). Stirred at 90 °C (oil bath) for 12 h, the reaction mixture was cooled to room temperature and extracted with ethyl acetate (150 mL). The organic layer was washed with saturated brine, dried with anhydrous Na_2SO_4, filtered and concentrated under reduced pressure. The residue was purified by flash column chromatography on silica gel (eluted with petroleum ether/ethyl acetate, 10/1, v/v) to afford compound **12** (0.5891 g) as a yellow solid in 59% yield. ^{1}H NMR (400 MHz, Chloroform-*d*) δ 10.04 (s, 1H), 8.08 (d, *J* = 7.84 Hz, 1H), 7.79 (m, 3H), 7.68 (t, *J* = 7.44 Hz, 1H), 7.54 (m, 2H), 7.49 (d, *J* = 8.40 Hz, 1H), 7.23 (s, 1H), 7.19 (d, *J* = 8.80 Hz, 1H). $^{13}C\{^{1}H\}$ NMR (100 MHz, Acetone-d_6) δ 192.2, 156.9, 146.8, 135.4, 134.8, 134.4, 133.1, 132.0, 130.8, 130.1, 129.0, 128.5, 128.1, 127.3, 120.1, 109.7. HRMS (ESI-IT-TOF) m/z: $[M+H]^+$ Calcd for $C_{17}H_{12}NaO_2^+$ 271.0730; Found: 271.0725. mp: 166.7-167.3 °C.

^{1}H NMR (400 MHz, Chloroform-*d*) spectrum of **compound 12**

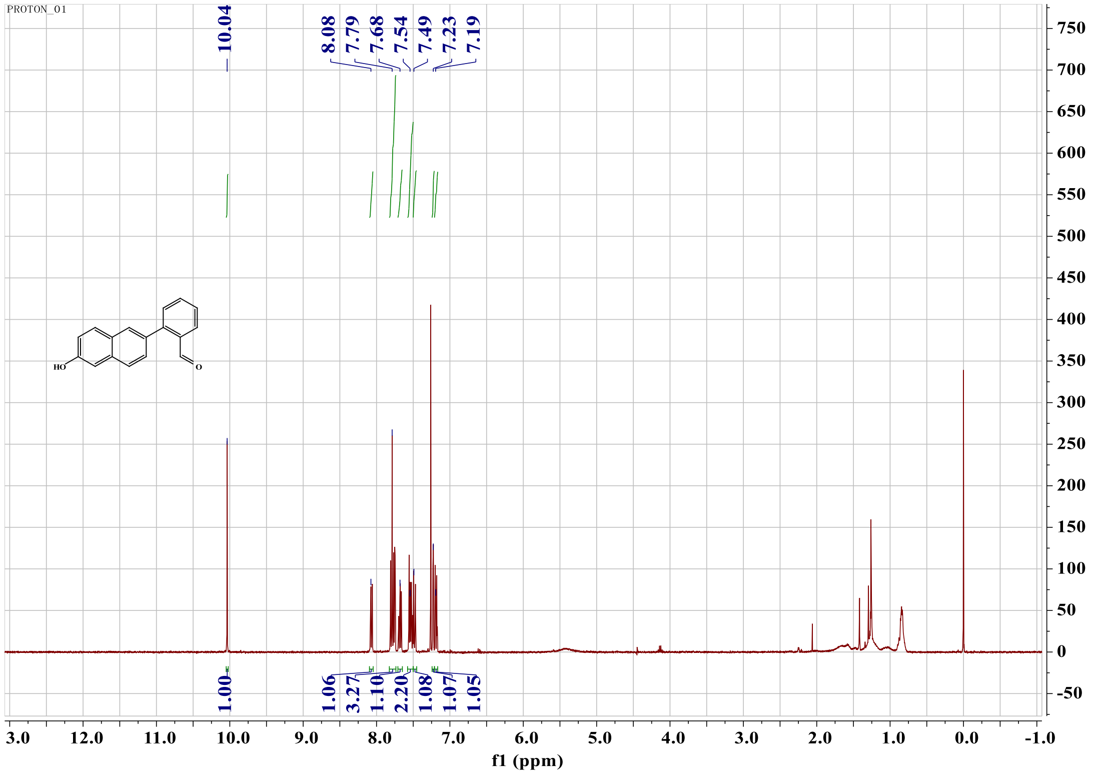

$^{13}C\{^{1}H\}$ NMR (100 MHz, Acetone-*d₆*) spectrum of **compound 12**

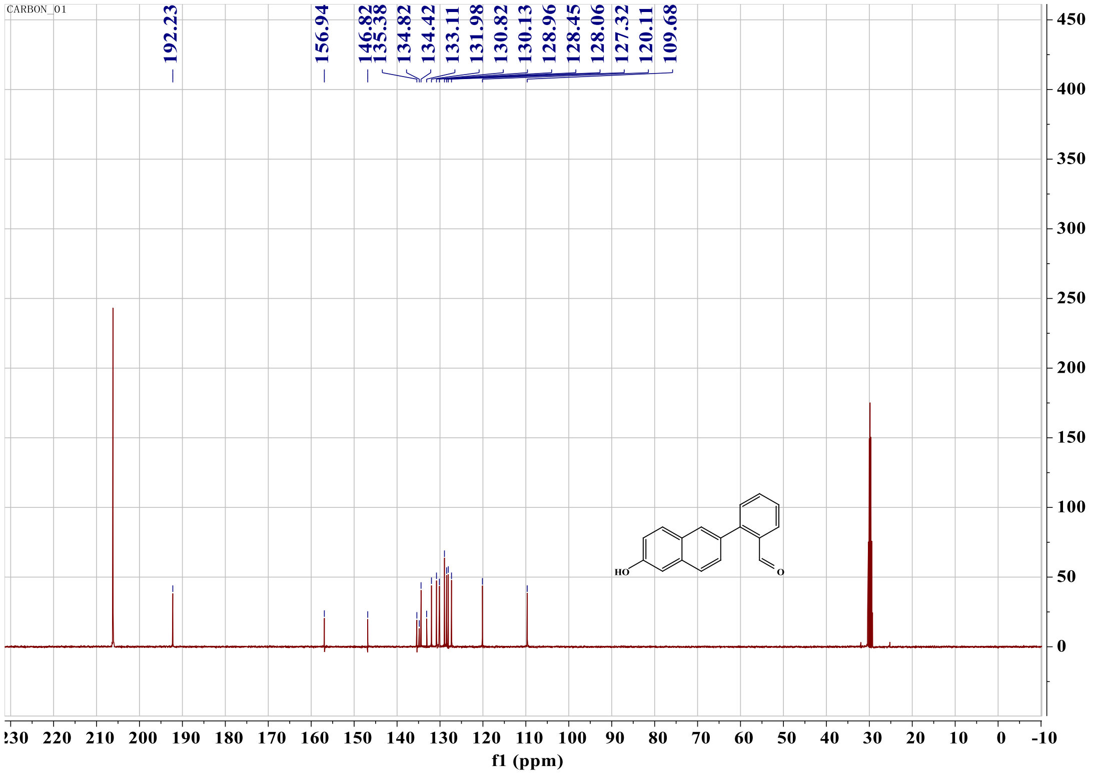

1.3.15. Synthesis and characterization of **14**.

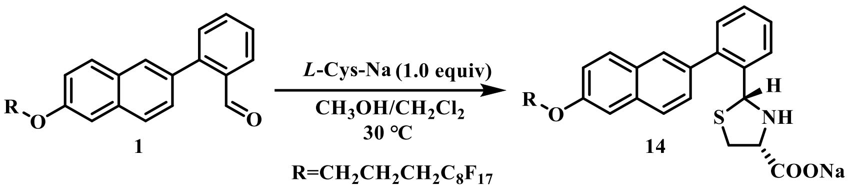

Under argon atmosphere, *L*-Cys (0.10 mmol, 0.0121 g, 1.0 equiv) and NaOH (0.1 mL, 1 M in CH_3OH, 1.0 equiv) were mixed in CH_3OH (9.5 mL) under ultrasound for 20 min to give clear and transparent methanol solution of *L*-Cys-Na. Then compound **1** (0.1 mmol, 0.0708 g, 1.0 equiv) in CH_2Cl_2 (0.5 mL) and *L*-Cys-Na in CH_3OH (9.5 mL) were stirred at 30 °C (oil bath) for 72 h. After filtration of reaction mixture, the filtrate was concentrated under reduced pressure to afford compound **14** (0.0834 g) as a light-yellow solid in quantitative yield. ^{1}H NMR (400 MHz, Methanol-*d*4) δ 7.91 (d, *J* = 8.12 Hz, 2H), 7.82 (m, 3H), 7.47 (m, 2H), 7.39 (t, *J* = 8.16 Hz, 2H), 7.31 (m, 2H), 7.22 (d, *J* = 8.72 Hz, 1H), 5.46 (s, 1H), 4.23 (t, *J* = 4.60 Hz, 2H), 3.53 (t, *J* = 7.92 Hz, 1H), 3.36 (m, 1H), 3.12 (m, 1H), 2.47 (m, 2H), 2.17 (m, 2H). $^{13}C\{^{1}H\}$ NMR (100 MHz, Methanol-*d*4) δ 178.0, 158.4, 143.4, 137.6, 137.0, 135.2, 131.3, 130.8, 130.2, 129.1, 129.1, 129.0, 128.9, 128.4, 127.8, 120.3, 135.1-120.8 (m), 107.6, 69.8, 69.6, 67.5, 41.4, 28.8 (t, *J* = 22.76 Hz), 21.7. ^{19}F NMR (376 MHz, MFethanol-*d*4) δ -82.35 (t, *J* = 8.84 Hz, 3F), -115.43 (m, 2F), -122.91 (m, 6F), -123.75 (m, 2F), -124.41 (m, 2F), -127.30 (m, 2F). HRMS (ESI-IT-TOF) m/z: $[M+H]^+$ Calcd for $C_{31}H_{22}F_{17}NNaO_3S^+$ 834.0941; Found: 834.0942. $[M-Na+2H]^+$ Calcd for $C_{31}H_{23}F_{17}NO_3S^+$ 812.1122; Found: 812.1134. $[\alpha]^{26.0}_{589}$ = -61.6 (c= 0.1, in $CHCl_3$). mp: 254.8-255.8 °C.

^{1}H NMR (400 MHz, Methanol-*d*4) spectrum of compound **14**

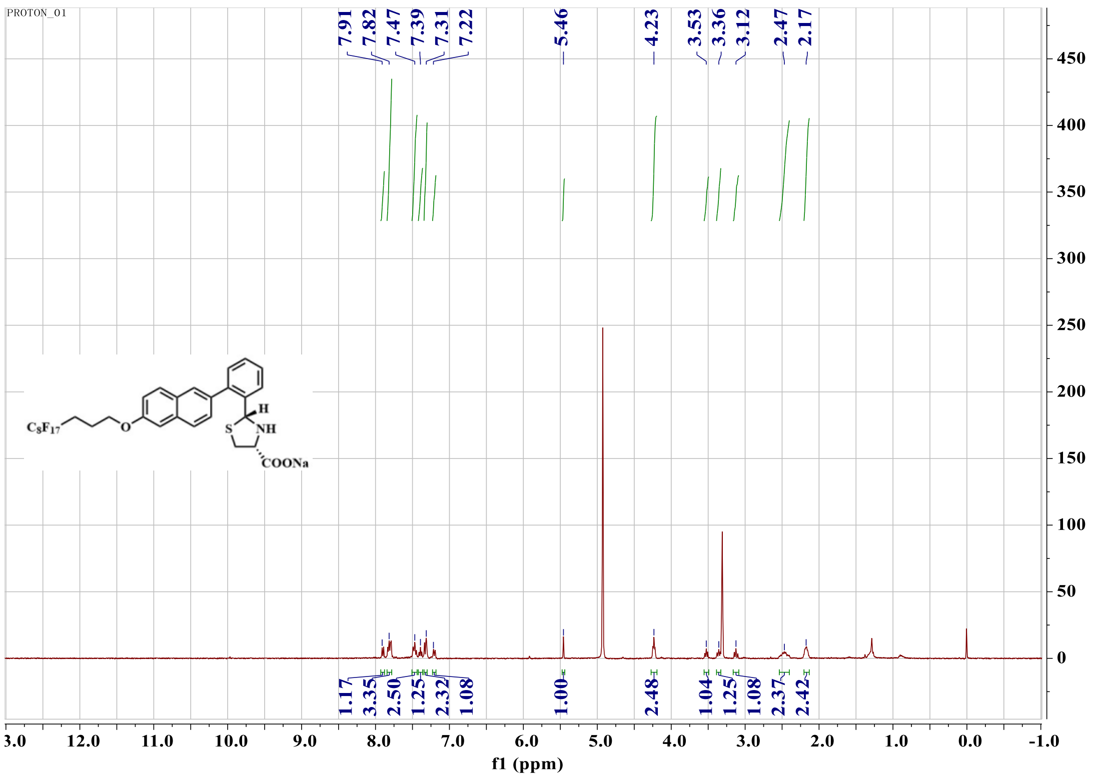

$^{13}C\{^{1}H\}$ NMR (100 MHz, Methanol-d_4) spectrum of compound **14**

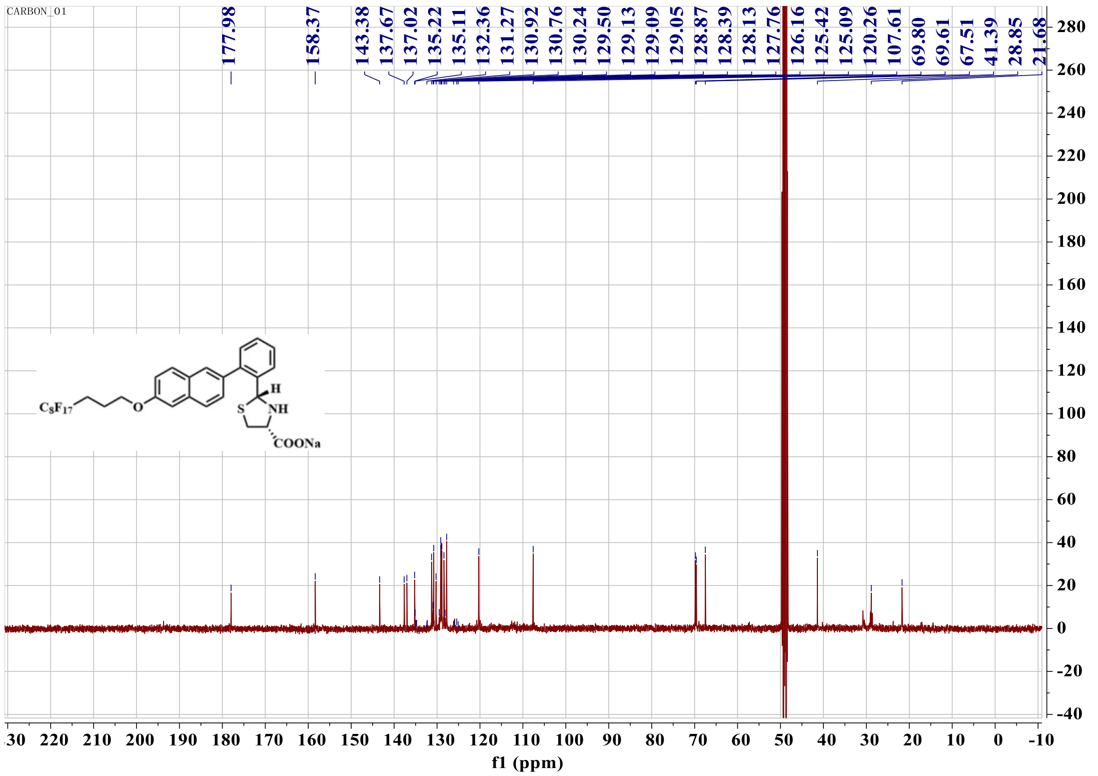

^{19}F NMR (376 MHz, Methanol-d_4) spectrum of compound **14**

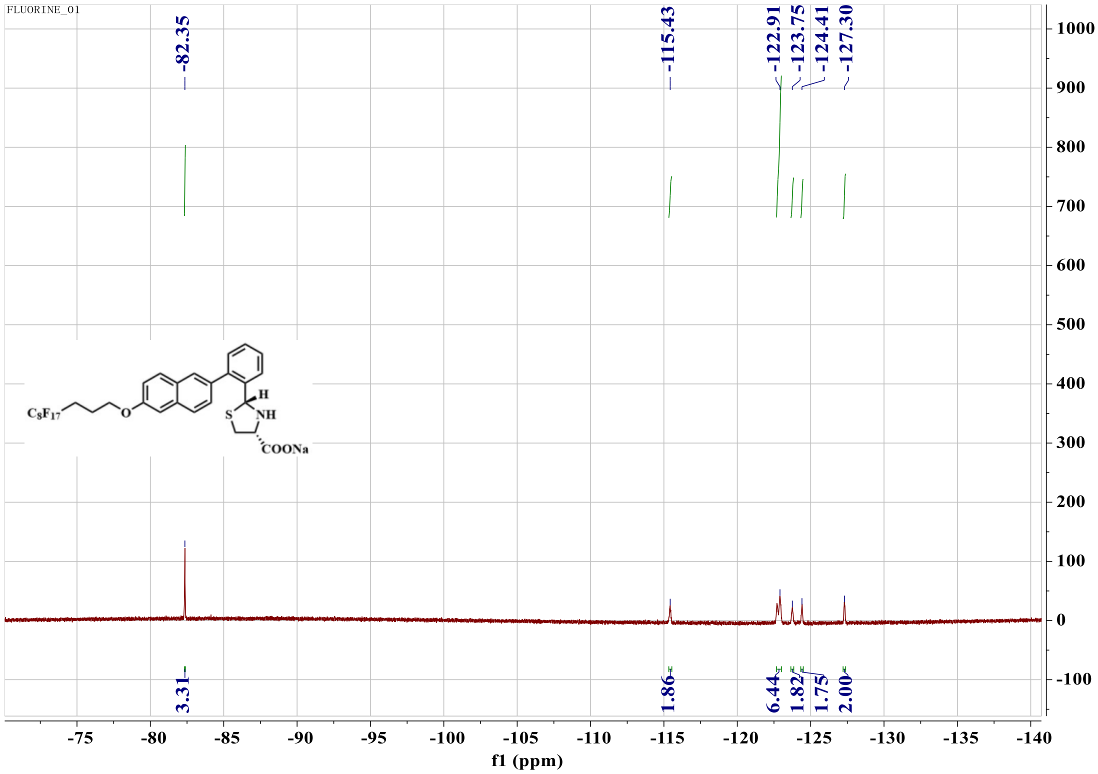

2. Optical measurements.

2.1. Sample preparation for fluorescence measurement.

Stock solutions of 5.0 mM (*R*)-**2** in CH_2Cl_2 and 50.0 mM TBA salts of amino acids were freshly prepared for each measurement. Amino acid-TBA salts were prepared by mixing amino acids and tetrabutylammonium hydroxide (TBAOH, 1M in methanol) with the equivalent of the carboxylic groups in methanol in situ. For optical analysis, solutions of (*R*)-**2** (5 μL each) were added to test tubes which contains 0.5 mL 2-(perfluorohexyl) ethyl alcohol (PEOH), then a solution of a TBA salt of an amino acid was added to each test tube. The resulting solution was put into an incubator at 298 K. After 120 min, the mixed solution of (*R*)-**2** of 5.0×10^{-5} M concentration in each test tube was used for fluorescence measurements. Fluorescence spectra were recorded within 10 min after the sample preparation.

2.2. Normalized UV-vis absorption and fluorescence spectra.

Figure S1. Normalized UV-vis absorption and fluorescence spectra of (*R*)-**2,** (*R*)-**7** and (*R*)-**8** (5.0×10^{-5} M in PEOH/CH_2Cl_2 = 99:1, v/v. λ_{exc} = 284 nm)

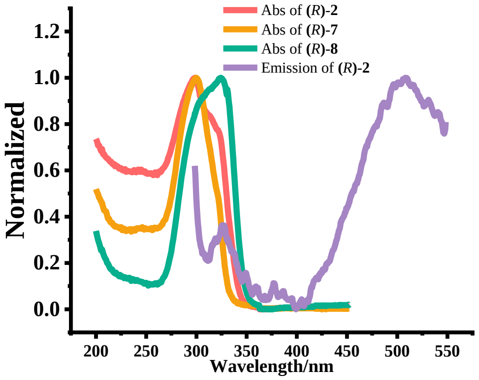

UV-vis spectra give absorption at λ (ε) = 300 (9.3×10^3) nm for (*R*)-**7** and 324 (9.7×10^3) nm for (*R*)-**8**.

2.3. Fluorescence spectra of (*R*)-2 in the fluorous phase (PEOH) with Cysteine and the other 19 common amino acids-TBAs.

Structures of amino acids studied:

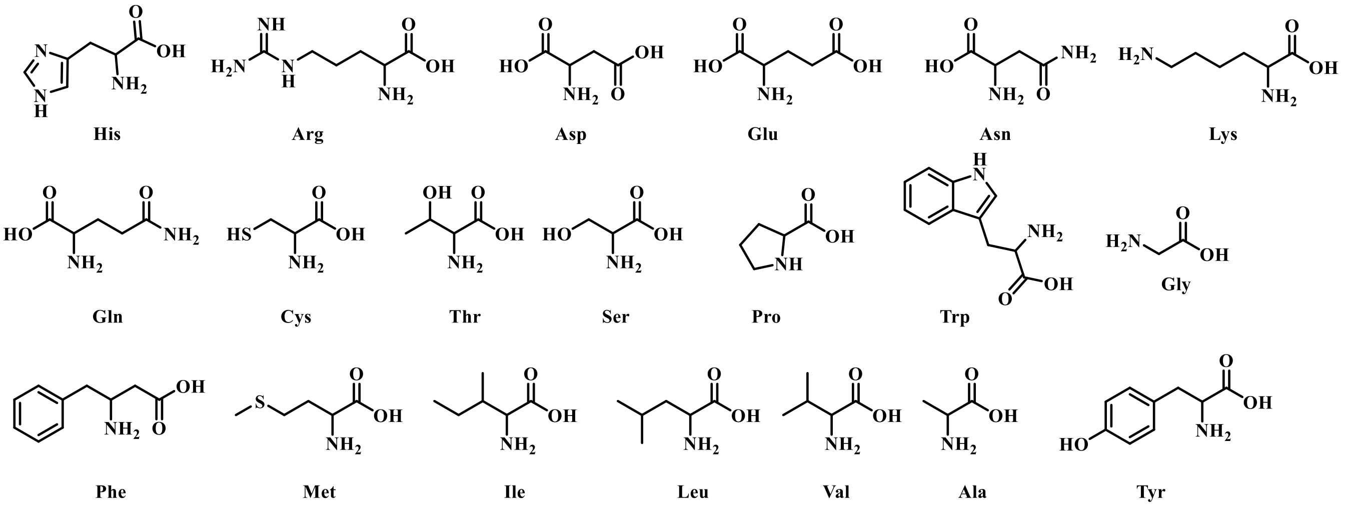

Figure S2. Fluorescent spectra of (*R*)-**2** (5.0×10^{-5} M) with Glycine and other 19 enantiomeric pairs of common amino acid-TBAs (16.0 equiv). (Solvent: PEOH/MeOH/CH_2Cl_2 = 98:1:1, v/v/v. λ_{exc} = 284 nm; slits = 5/5 nm; reaction time: 120 min; temperature: 25 °C).

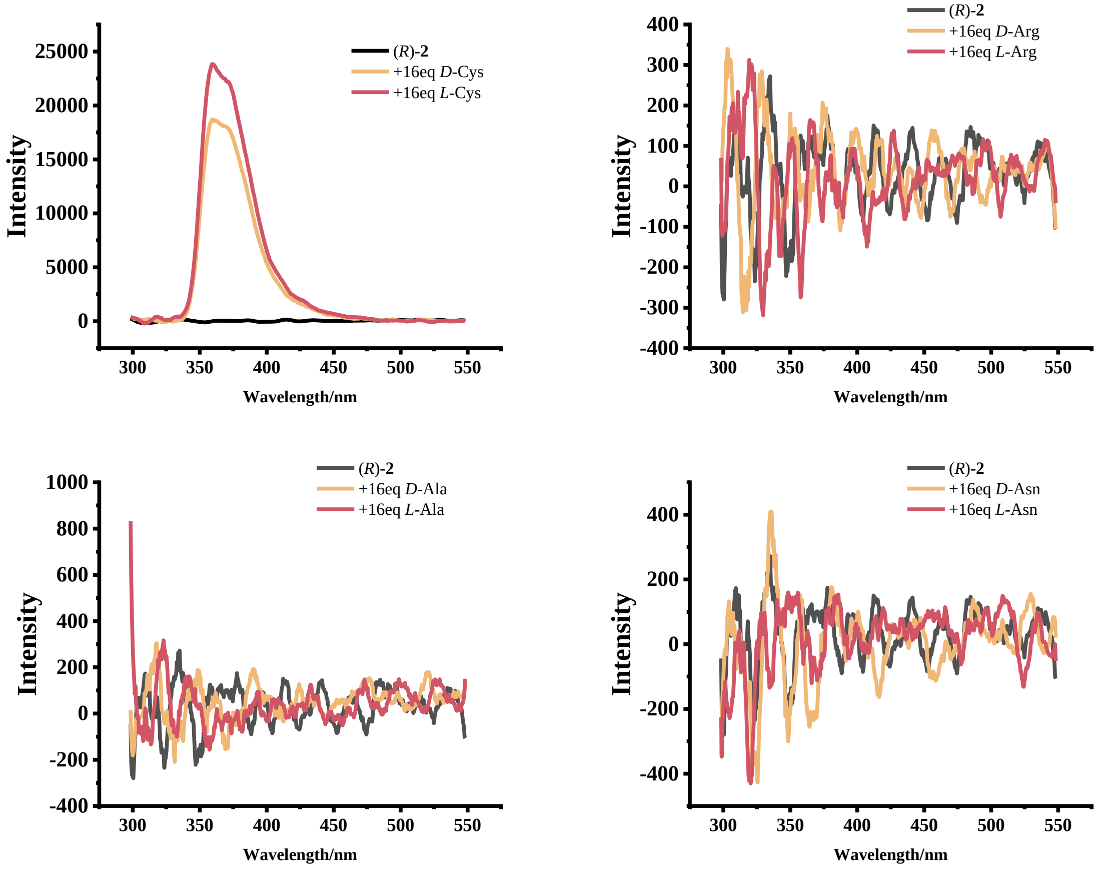

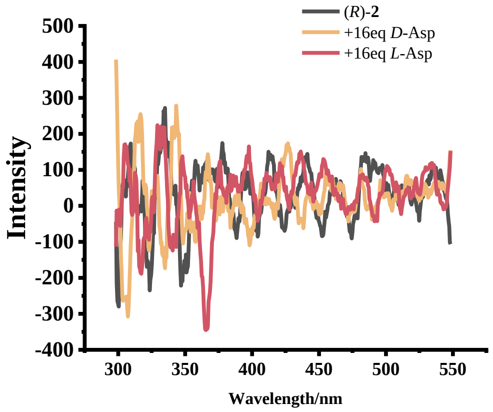
(R)-2
+16eq D-Asp
+16eq L-Asp
Intensity
Wavelength/nm

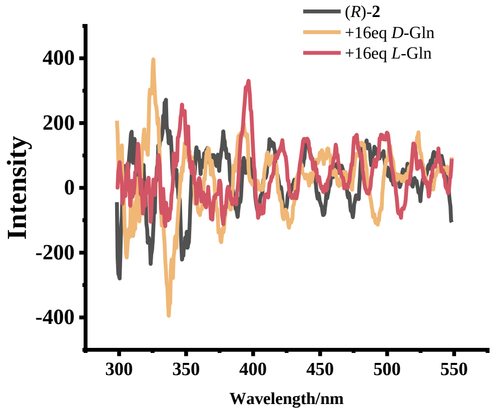
(R)-2
+16eq D-Gln
+16eq L-Gln
Intensity
Wavelength/nm

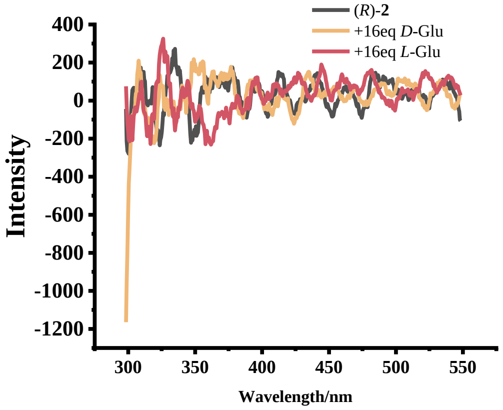
(R)-2
+16eq D-Glu
+16eq L-Glu
Intensity
Wavelength/nm

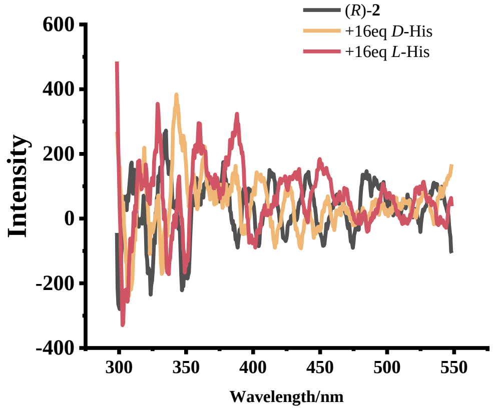
(R)-2
+16eq D-His
+16eq L-His
Intensity
Wavelength/nm

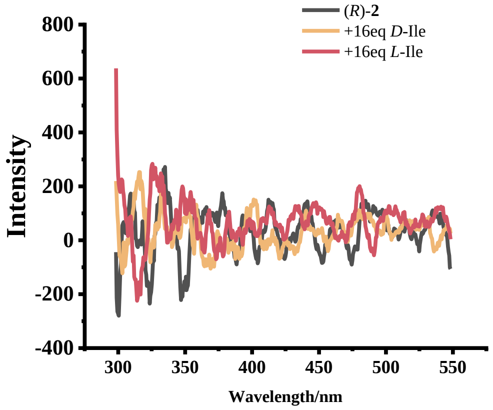
(R)-2
+16eq D-Ile
+16eq L-Ile
Intensity
Wavelength/nm

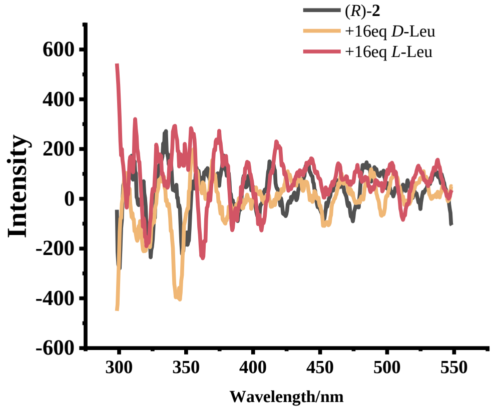
(R)-2
+16eq D-Leu
+16eq L-Leu
Intensity
Wavelength/nm

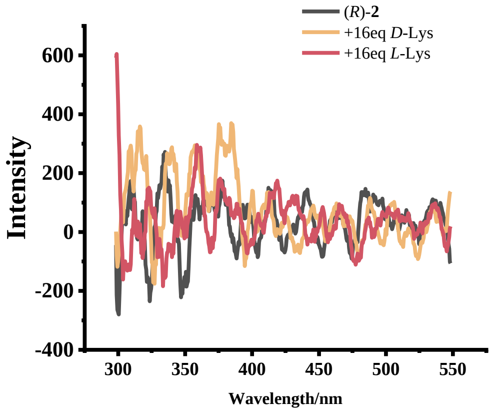
(R)-2
+16eq D-Lys
+16eq L-Lys
Intensity
Wavelength/nm

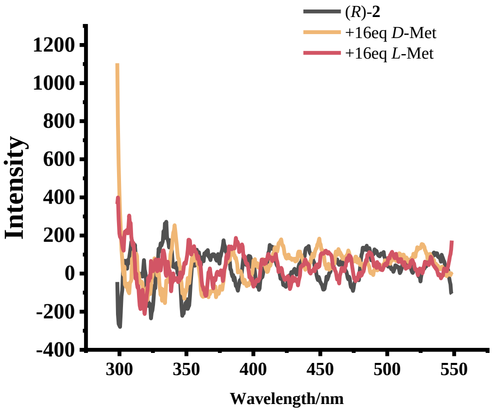
(R)-2
+16eq D-Met
+16eq L-Met
Intensity
Wavelength/nm

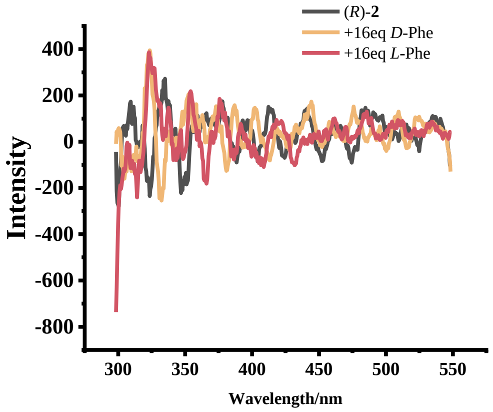
(R)-2
+16eq D-Phe
+16eq L-Phe
Intensity
Wavelength/nm

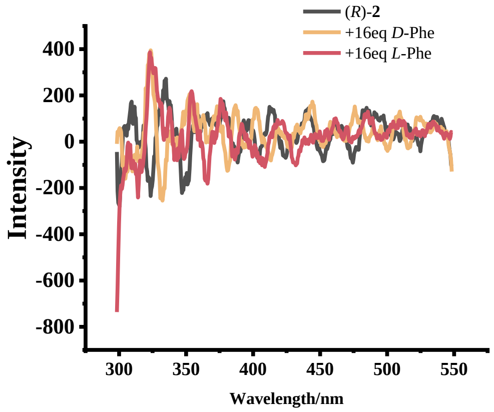
(R)-2
+16eq D-Phe
+16eq L-Phe
Intensity
Wavelength/nm

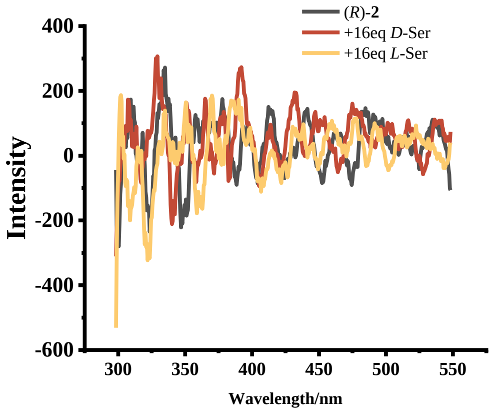
(R)-2
+16eq D-Ser
+16eq L-Ser
Intensity
Wavelength/nm

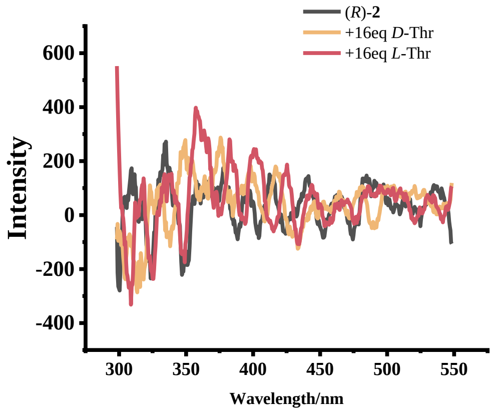
(R)-2
+16eq D-Thr
+16eq L-Thr
Intensity
Wavelength/nm

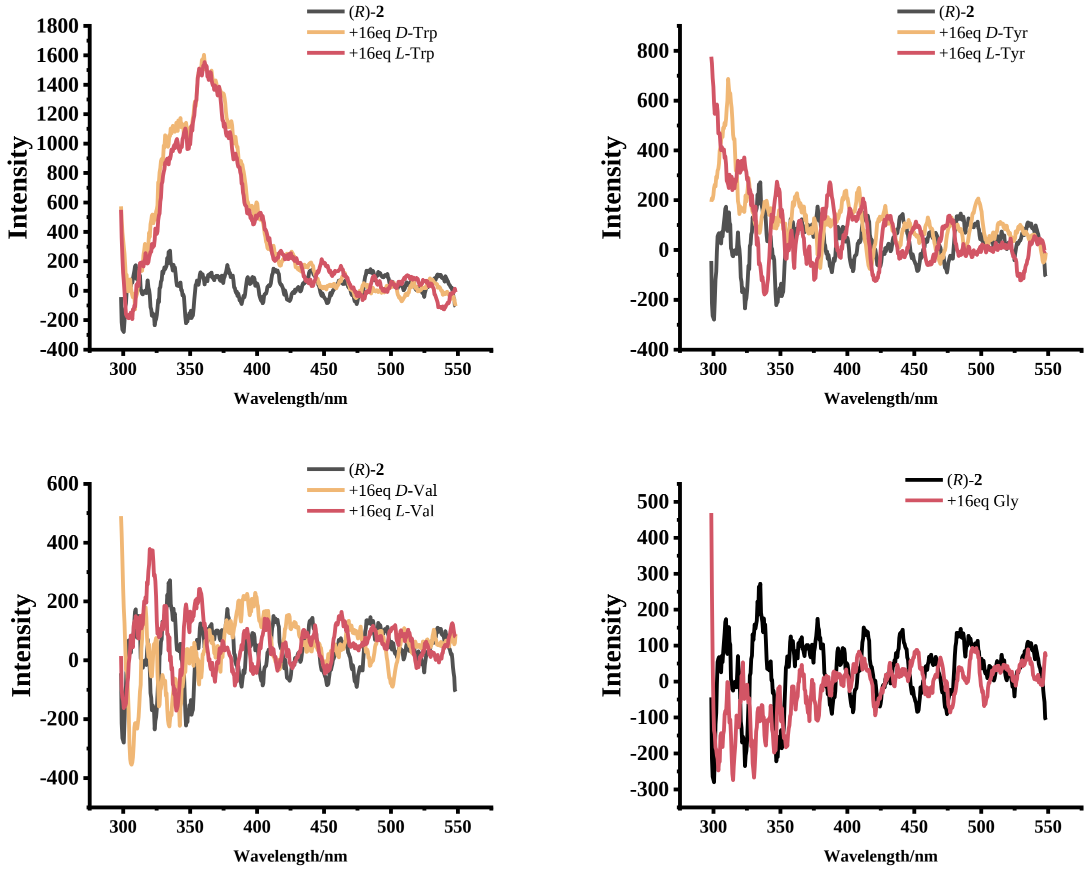

Figure S3. Fluorescent spectra of (*R*)-**7** (5.0×10^{-5} M) with Glycine and other 19 enantiomeric pairs of common amino acid-TBAs (16.0 equiv). (Solvent: PEOH/MeOH/CH_2Cl_2 = 98:1:1, v/v/v. λ_{exc} = 284 nm; slits = 5/5 nm; reaction time: 120 min; temperature: 25 °C; I_0: fluorescence intensity of (*R*)-**7** at 360 nm without amino acids.)

Plot of the fluorescence intensity at 360 nm, I_{360}/I_0:

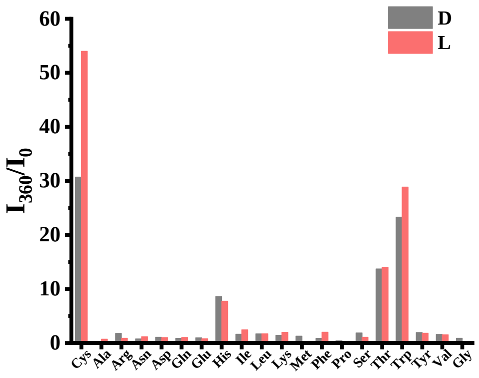

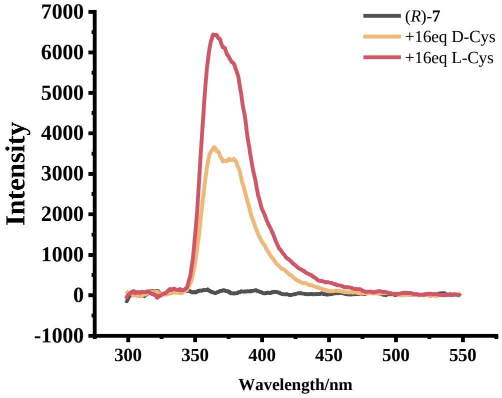
(R)-7
+16eq D-Cys
+16eq L-Cys
Intensity
Wavelength/nm

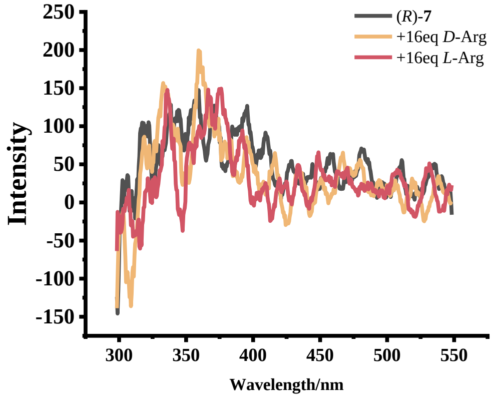
(R)-7
+16eq D-Arg
+16eq L-Arg
Intensity
Wavelength/nm

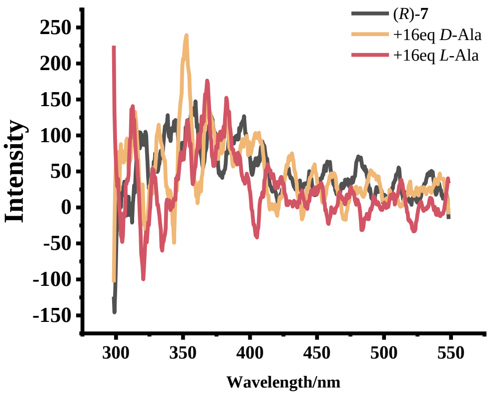
(R)-7
+16eq D-Ala
+16eq L-Ala
Intensity
Wavelength/nm

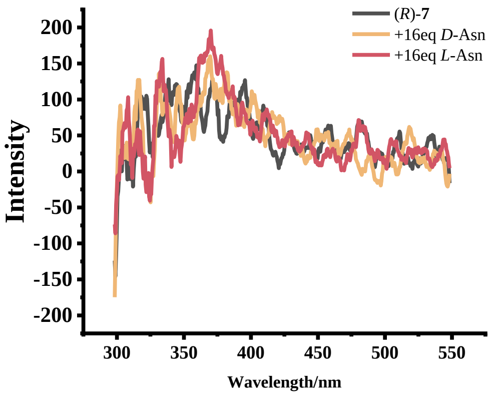
(R)-7
+16eq D-Asn
+16eq L-Asn
Intensity
Wavelength/nm

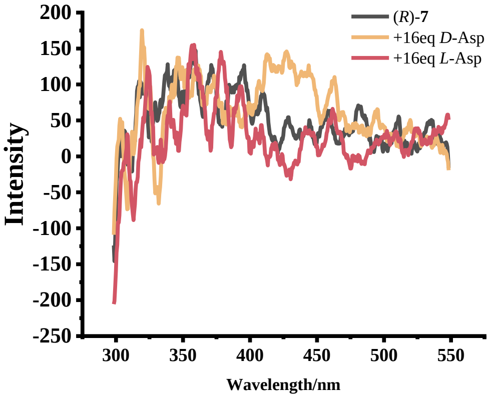
(R)-7
+16eq D-Asp
+16eq L-Asp
Intensity
Wavelength/nm

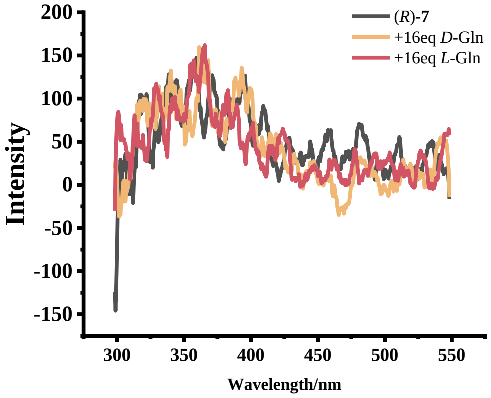
(R)-7
+16eq D-Gln
+16eq L-Gln
Intensity
Wavelength/nm

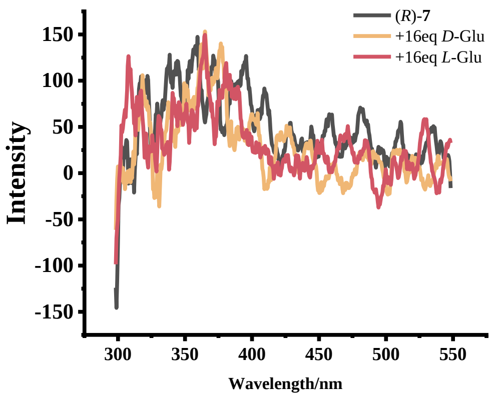
(R)-7
+16eq D-Glu
+16eq L-Glu
Intensity
Wavelength/nm

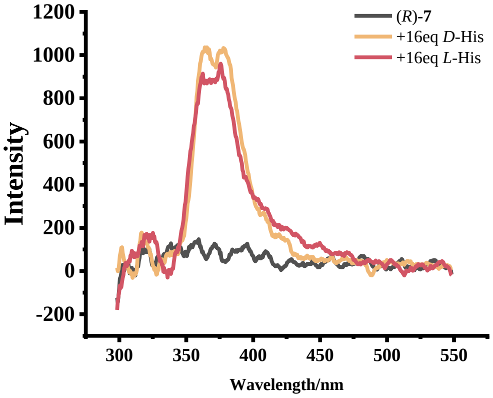
(R)-7
+16eq D-His
+16eq L-His
Intensity
Wavelength/nm

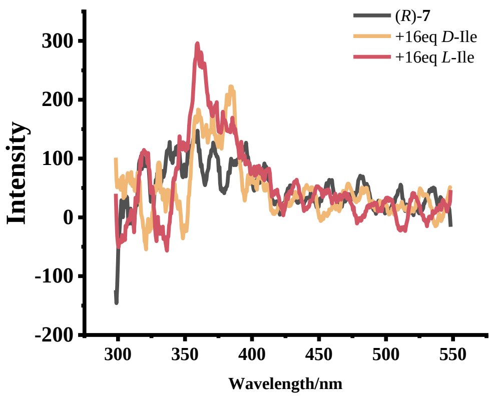
(R)-7
+16eq D-Ile
+16eq L-Ile
Intensity
Wavelength/nm

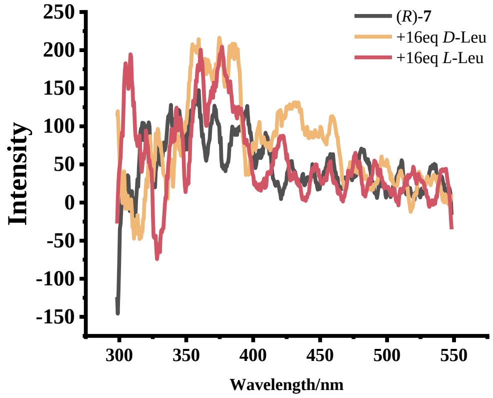
(R)-7
+16eq D-Leu
+16eq L-Leu
Intensity
Wavelength/nm

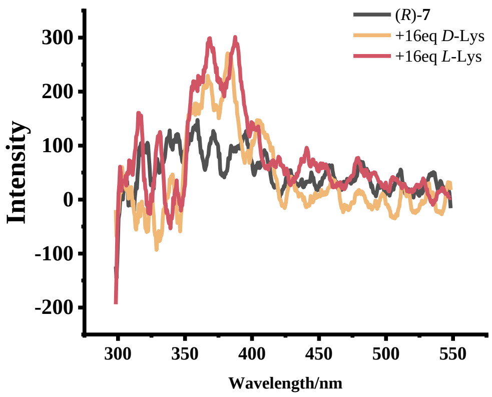
(R)-7
+16eq D-Lys
+16eq L-Lys
Intensity
Wavelength/nm

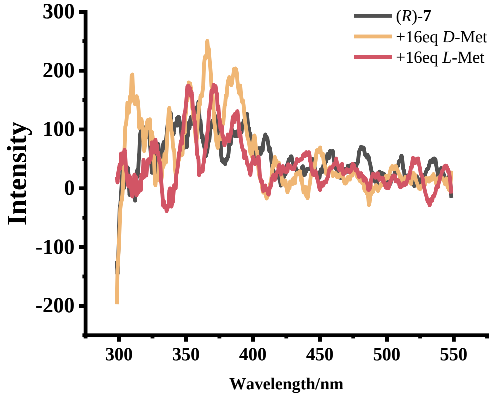
(R)-7
+16eq D-Met
+16eq L-Met
Intensity
Wavelength/nm

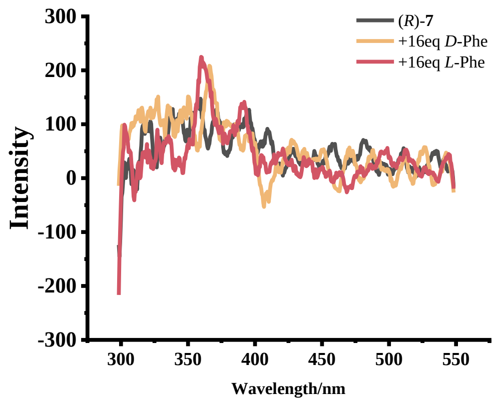
(R)-7
+16eq D-Phe
+16eq L-Phe
Intensity
300
200
100
0
-100
-200
-300
300
350
400
450
500
550
Wavelength/nm

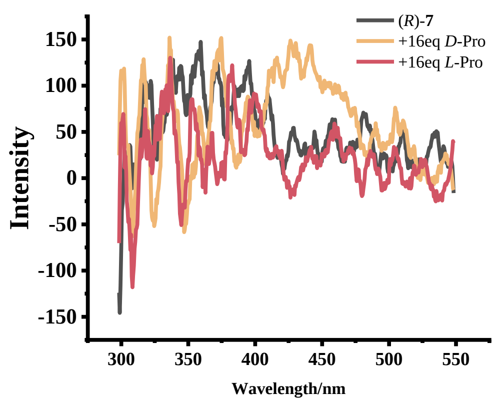
(R)-7
+16eq D-Pro
+16eq L-Pro
Intensity
150
100
50
0
-50
-100
-150
300
350
400
450
500
550
Wavelength/nm

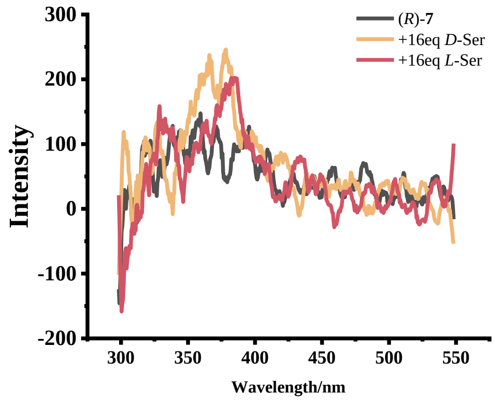
(R)-7
+16eq D-Ser
+16eq L-Ser
Intensity
300
200
100
0
-100
-200
300
350
400
450
500
550
Wavelength/nm

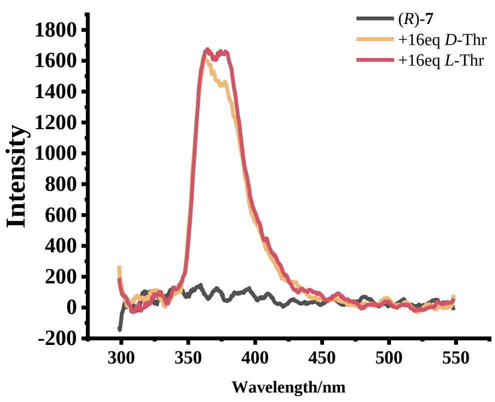
(R)-7
+16eq D-Thr
+16eq L-Thr
Intensity
1800
1600
1400
1200
1000
800
600
400
200
0
-200
300
350
400
450
500
550
Wavelength/nm

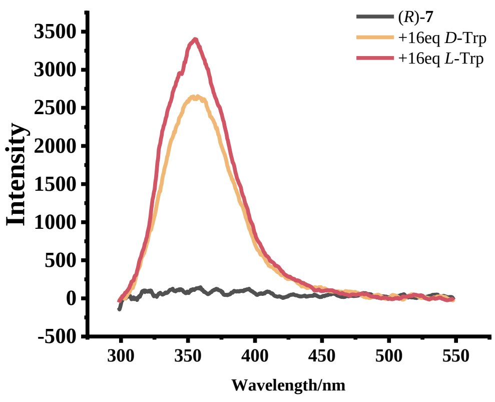
(R)-7
+16eq D-Trp
+16eq L-Trp
Intensity
3500
3000
2500
2000
1500
1000
500
0
-500
300
350
400
450
500
550
Wavelength/nm

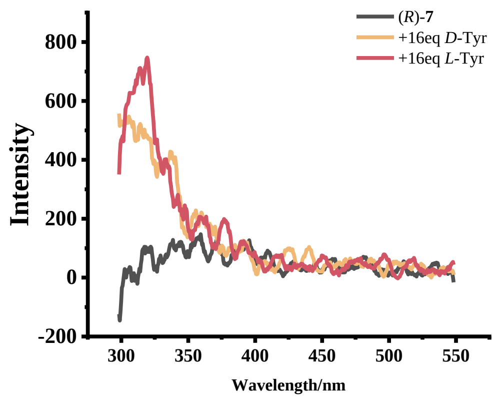
(R)-7
+16eq D-Tyr
+16eq L-Tyr
Intensity
800
600
400
200
0
-200
300
350
400
450
500
550
Wavelength/nm

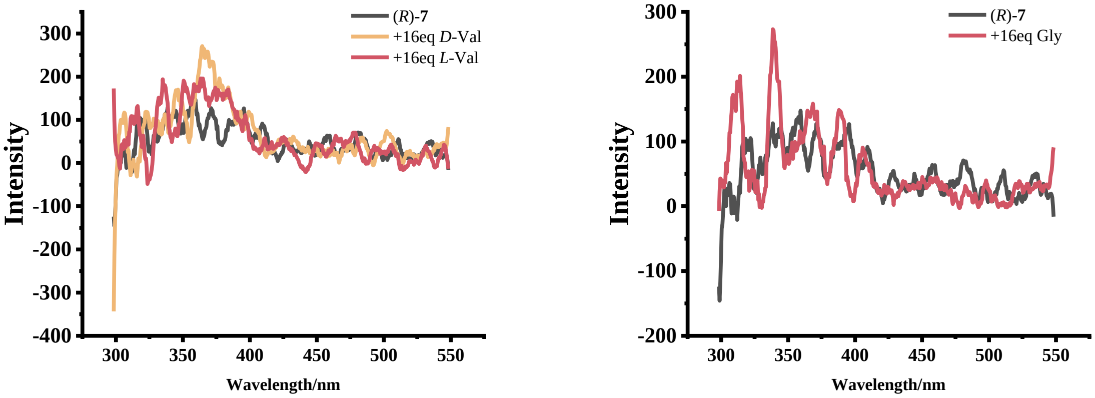

Figure S4. Fluorescent spectra of (*R*)-**8** (5.0×10^{-5} M) with Glycine and other 19 enantiomeric pairs of common amino acid-TBAs (16.0 equiv). (Solvent: PEOH/MeOH/CH_2Cl_2 = 98:1:1, v/v/v. λ_{exc} = 284 nm; slits = 5/5 nm; reaction time: 120 min; temperature: 25 °C; I_0: fluorescence intensity of (*R*)-**8** at 360 nm without amino acids.)

Plot of the fluorescence intensity at 360 nm, I_{360}/I_0:

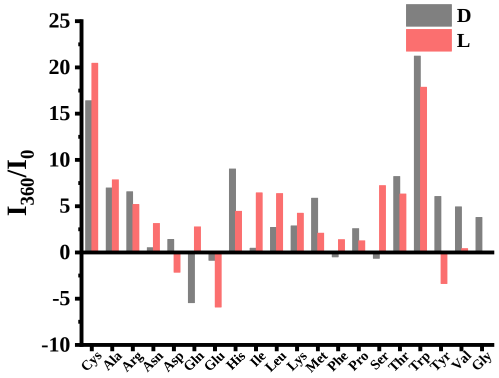

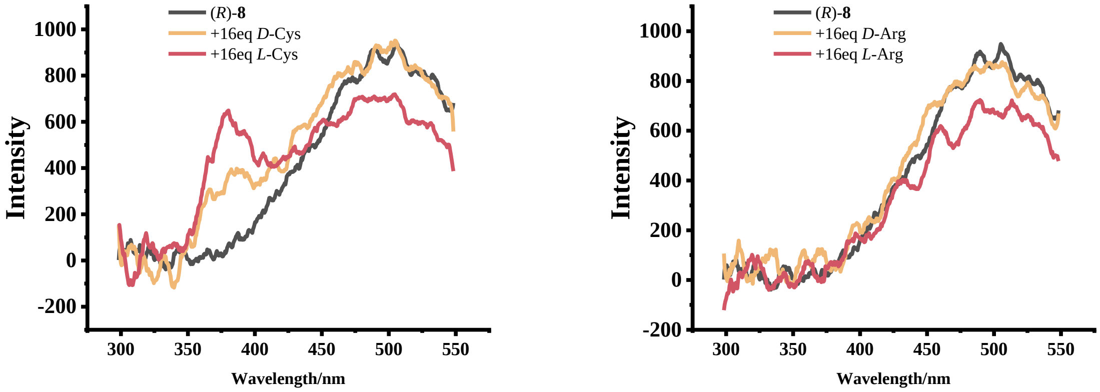

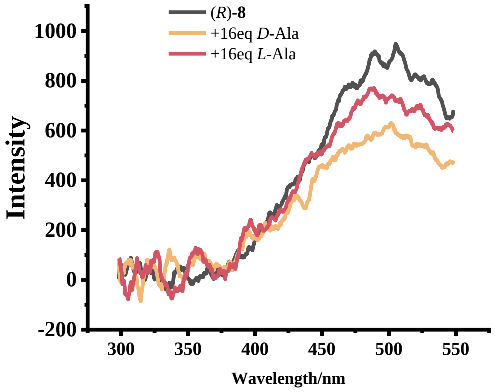
(*R*)-**8**
+16eq *D*-Ala
+16eq *L*-Ala
Intensity
Wavelength/nm

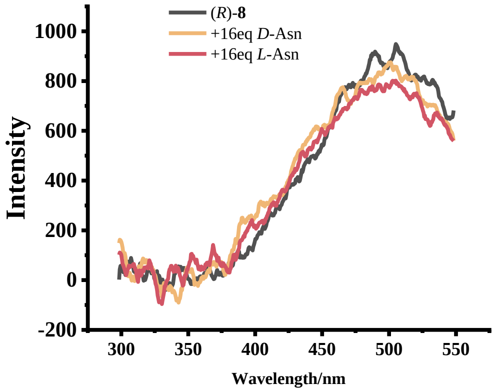
(*R*)-**8**
+16eq *D*-Asn
+16eq *L*-Asn
Intensity
Wavelength/nm

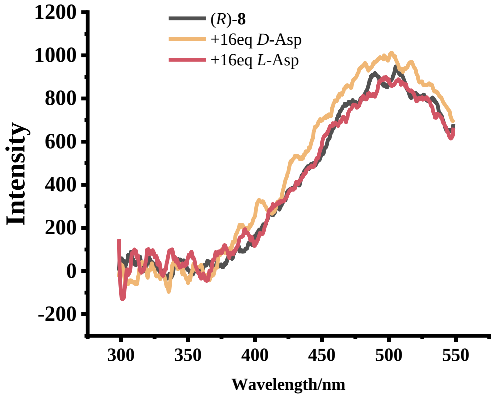
(*R*)-**8**
+16eq *D*-Asp
+16eq *L*-Asp
Intensity
Wavelength/nm

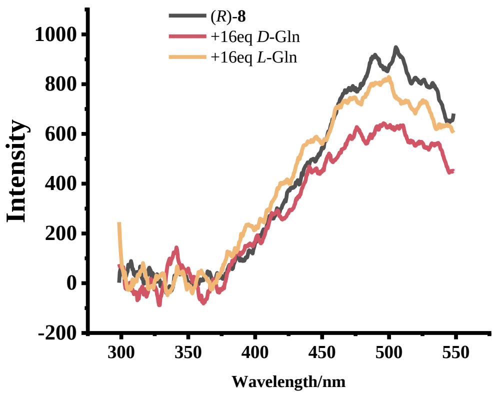
(*R*)-**8**
+16eq *D*-Gln
+16eq *L*-Gln
Intensity
Wavelength/nm

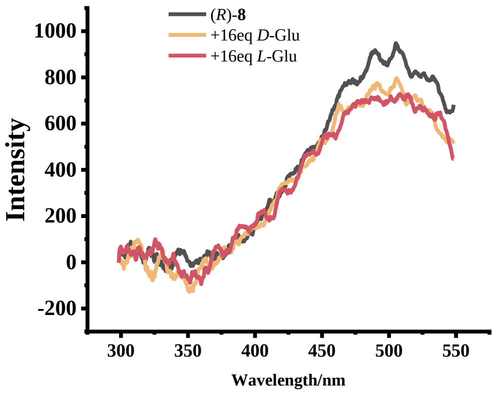
(*R*)-**8**
+16eq *D*-Glu
+16eq *L*-Glu
Intensity
Wavelength/nm

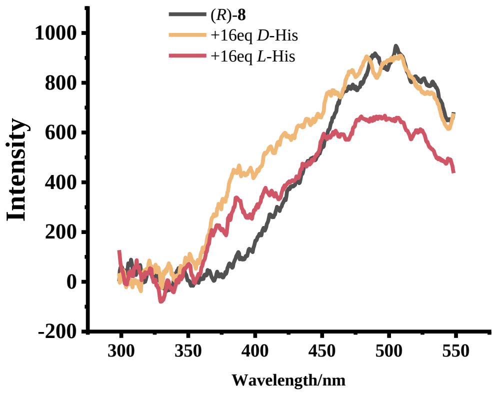
(*R*)-**8**
+16eq *D*-His
+16eq *L*-His
Intensity
Wavelength/nm

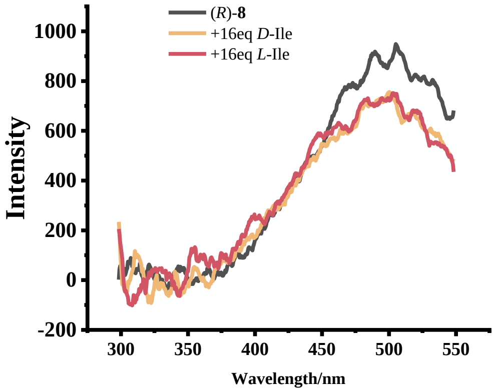
(R)-8
+16eq D-Ile
+16eq L-Ile
Intensity
Wavelength/nm
-200
0
200
400
600
800
1000
300
350
400
450
500
550

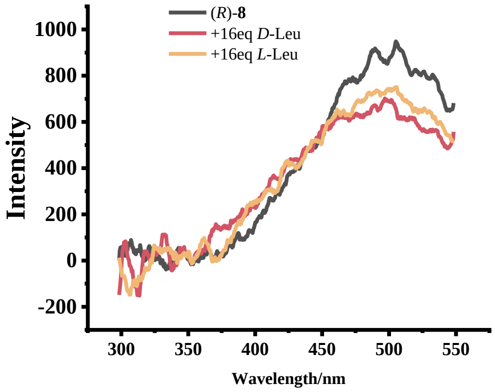
(R)-8
+16eq D-Leu
+16eq L-Leu
Intensity
Wavelength/nm
-200
0
200
400
600
800
1000
300
350
400
450
500
550

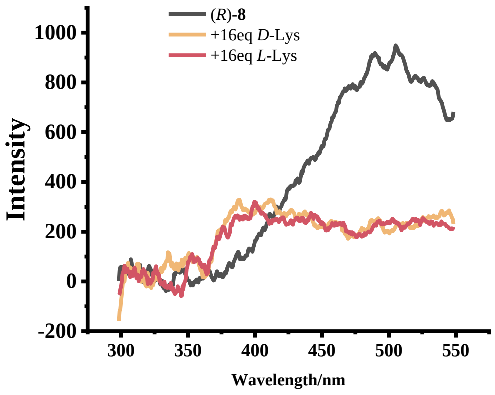
(R)-8
+16eq D-Lys
+16eq L-Lys
Intensity
Wavelength/nm
-200
0
200
400
600
800
1000
300
350
400
450
500
550

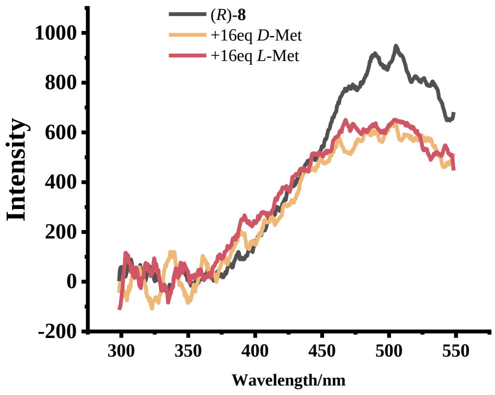
(R)-8
+16eq D-Met
+16eq L-Met
Intensity
Wavelength/nm
-200
0
200
400
600
800
1000
300
350
400
450
500
550

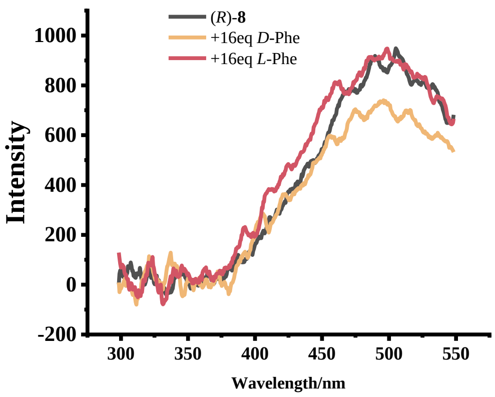
(R)-8
+16eq D-Phe
+16eq L-Phe
Intensity
Wavelength/nm
-200
0
200
400
600
800
1000
300
350
400
450
500
550

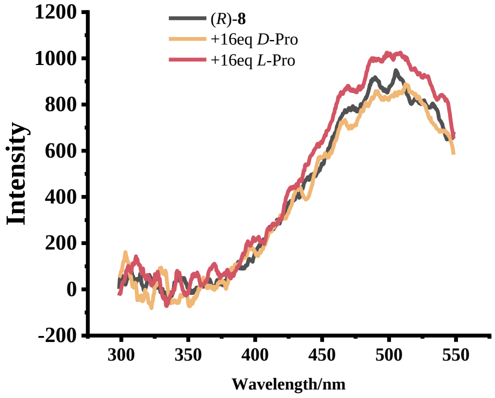
(R)-8
+16eq D-Pro
+16eq L-Pro
Intensity
Wavelength/nm
-200
0
200
400
600
800
1000
1200
300
350
400
450
500
550

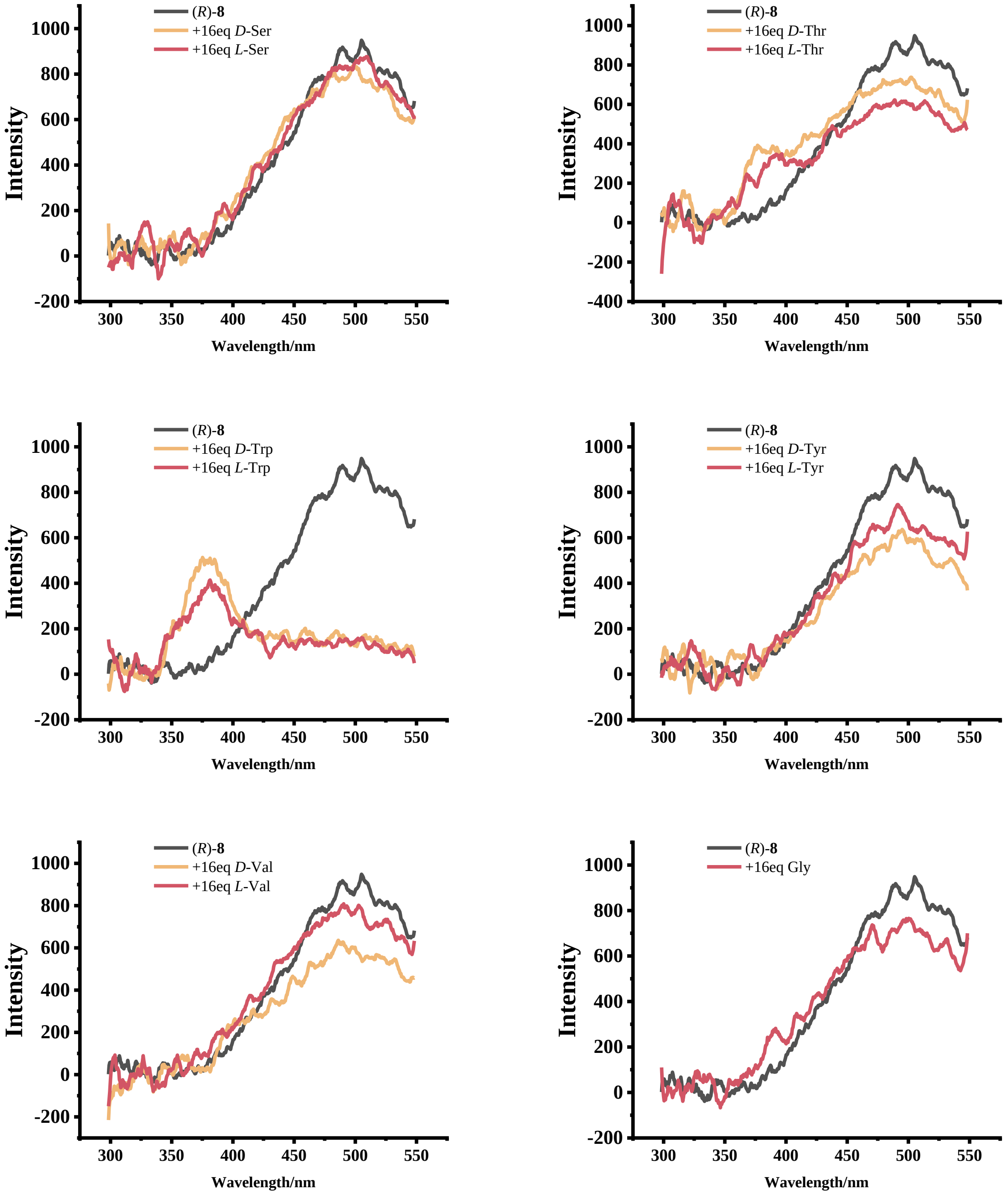
(R)-8
+16eq D-Ser
+16eq L-Ser
(R)-8
+16eq D-Thr
+16eq L-Thr
(R)-8
+16eq D-Trp
+16eq L-Trp
(R)-8
+16eq D-Tyr
+16eq L-Tyr
(R)-8
+16eq D-Val
+16eq L-Val
(R)-8
+16eq Gly
Intensity
Wavelength/nm

2.4. Fluorescence spectra of (*R*)-2 with 2.0-20.0 equivalent *D*-Cys-TBA.

Figure S5. Fluorescence spectra of (*R*)-**2** (5.0×10^{-3} M, 5 μL in CH_2Cl_2) with 2.0 - 10.0 equivalent *L*-/*D*-Cys-TBA (10, 20, 30, 40, 50 μL of 5.0×10^{-3} M stock solution in MeOH) and 12.0 - 20.0 equivalent *L*-/*D*-Cys-TBA (6, 7, 8, 9, 10 μL of 5.0×10^{-2} M stock solution in MeOH) in PEOH (0.5 mL). Additional methanol was added to the test tube to give a final volume of 555 μL (Solvent: PEOH/MeOH/CH_2Cl_2 = 90:9:1, v/v/v. λ_{exc} = 284 nm; slits = 5/5 nm; reaction time: 120 min; temperature: 25 °C).

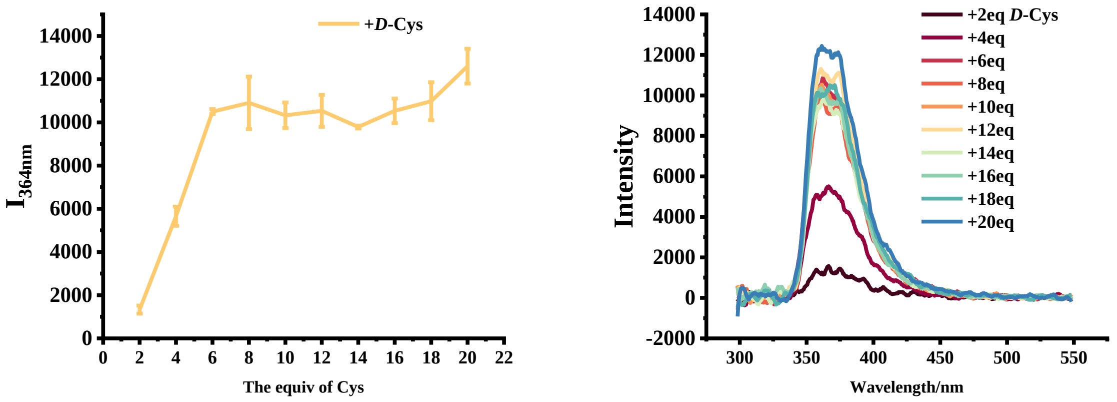

2.5. Fluorescence intensity at 360 nm for (*S*)-2 with *L*-Cys and *D*-Cys-TBA versus the reaction time.

Figure S6. Fluorescence intensity at 360 nm for (*S*)-**2** (5.0×10^{-5} M) with *L*- and *D*-Cys-TBA (16.0 equiv) vs the reaction time of 2, 6, 10, 20, 30, 40, 50, 60, 80, 100, 120, 150, 180 min respectively. (Solvent: 98:1:1 (v:v:v) PEOH/MeOH/CH_2Cl_2; λ_{exc} = 284 nm; slits, 5/5 nm; temperature, 25°C).

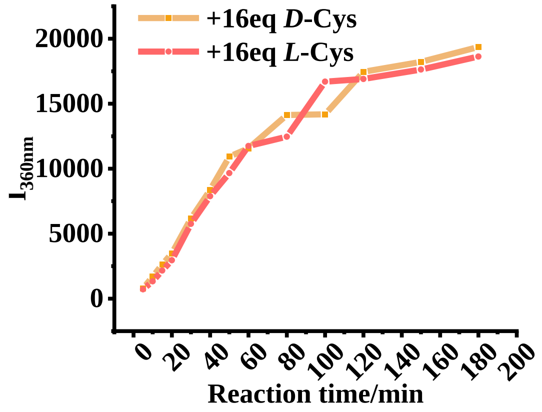

2.6. The limit of detection for *L*-Cys-TBA by using (*R*)-2.

Figure S7. Determination of the limit of detection for the fluorescence response of (*R*)-**2** (5×10^{-3} M, 20 μL in CH_2Cl_2) and PEOH (2.0 mL) upon addition of *L*-Cys-TBA (22, 24, 26, 28, 30, 32, 34, 36, 38, 40 μL of 5 mM stock solution in MeOH respectively). Because of the different volumes of cysteine added, additional methanol was added to the test tube for a final total volume of 2060 μL (solvent: 98:1:1 (v:v:v) PEOH/MeOH/CH_2Cl_2; λ_{exc} = 284 nm; slits, 10/10 nm; reaction time, 120 min at 25 °C; Error bars from three independent experiments).

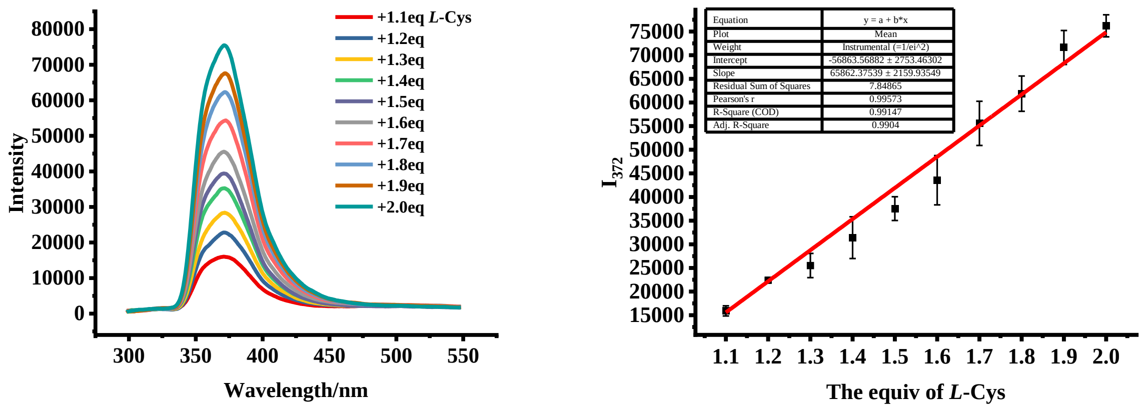

Standard deviation: 79.5719

Limit of detection (LOD)

$$= (3 \times 79.5719)/65862.37539 \times 10^{-6} = 3.6\times10^{-9} \text{ M}$$

2.7. The limit of quantification for *L*-Cys-TBA by using (*R*)-2.

Figure S8. Fluorescence spectra of (*R*)-**2** (5.0×10^{-5} M) with 0.1 - 1.0 equiv *L*-Cys-TBA. (Solvent: 98:1:1 (v:v:v) PEOH/MeOH/CH_2Cl_2; λ_{exc} = 284 nm; slits, 10/10 nm; reaction time, 120 min at 25 °C).

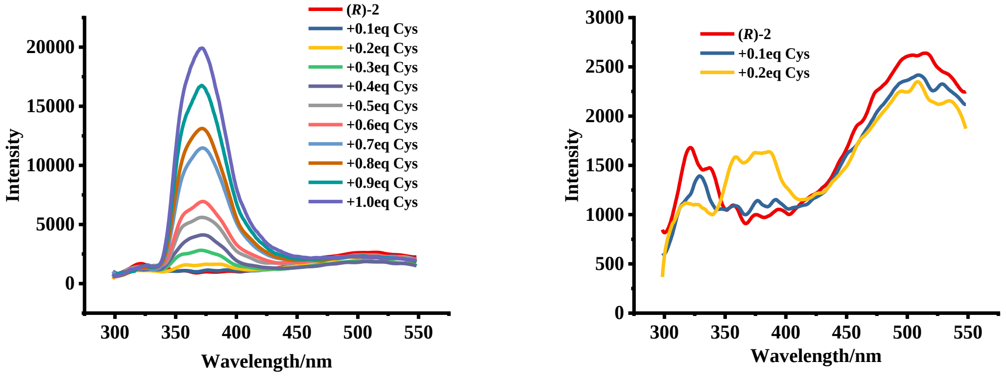

Figure S9. Fluorescence intensity at λ = 372 nm, I_{372}, from the interaction of (*R*)-**2** (5.0×10^{-5} M) with 0 - 1.0 equiv *L*-Cys-TBA. (Solvent: 98:1:1 (v:v:v) PEOH/MeOH/CH_2Cl_2; λ_{exc} = 284 nm; slits, 10/10 nm; reaction time, 120 min at 25 °C; Error bars from three independent experiments).

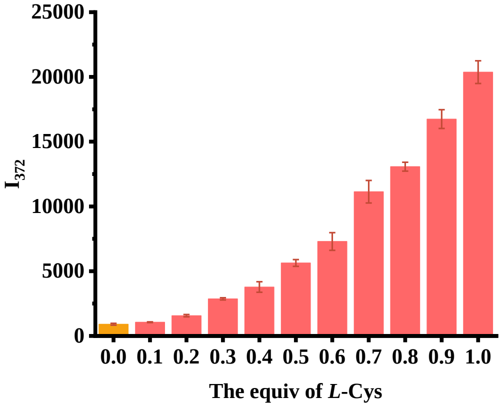

2.8. Determination of the concentration of cysteine samples.

Figure S10. The standard curve for the fluorescence response of (*R*)-**2** (5×10^{-3} M, 20 μL in CH_2Cl_2) and PEOH (2.0 mL) upon addition of *L*-Cys-TBA (22, 26, 30, 34, 38 μL of 5 mM stock solution in MeOH respectively). Because of the different volumes of cysteine added, additional methanol was added to the test tube for a final total volume of 2060 μL (Solvent: 97:2:1 (v:v:v) PEOH/MeOH/CH_2Cl_2; λ_{exc} = 284 nm; slits, 10/10 nm; reaction time, 120 min at 25 °C; Error bars from three independent experiments).

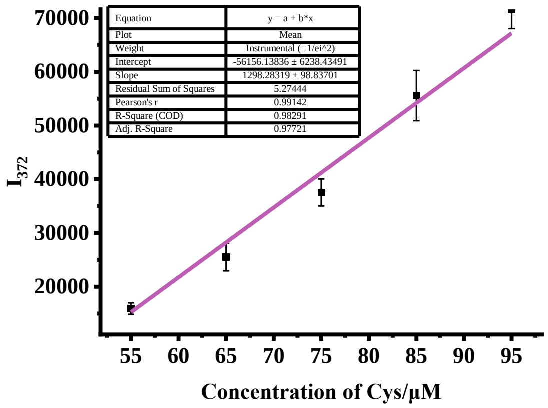

Figure S11. Fluorescence response of (*R*)-**2** (5×10^{-3} M, 20 μL in CH_2Cl_2) and PEOH (2.0 mL) upon addition of *L*-Cys-TBA (24, 28, 32, 36, 40 μL of 5 mM stock solution in MeOH respectively). Because of the different volumes of cysteine added, additional methanol was added to the test tube for a final total volume of 2060 μL. The standard curve in **Figure S10.** was applied to determine the concentration of cysteine (Solvent: 97:2:1 (v:v:v) PEOH/MeOH/CH_2Cl_2; λ_{exc} = 284 nm; slits, 10/10 nm; reaction time, 120 min at 25 °C; all the plots were obtained from three independent experiments).

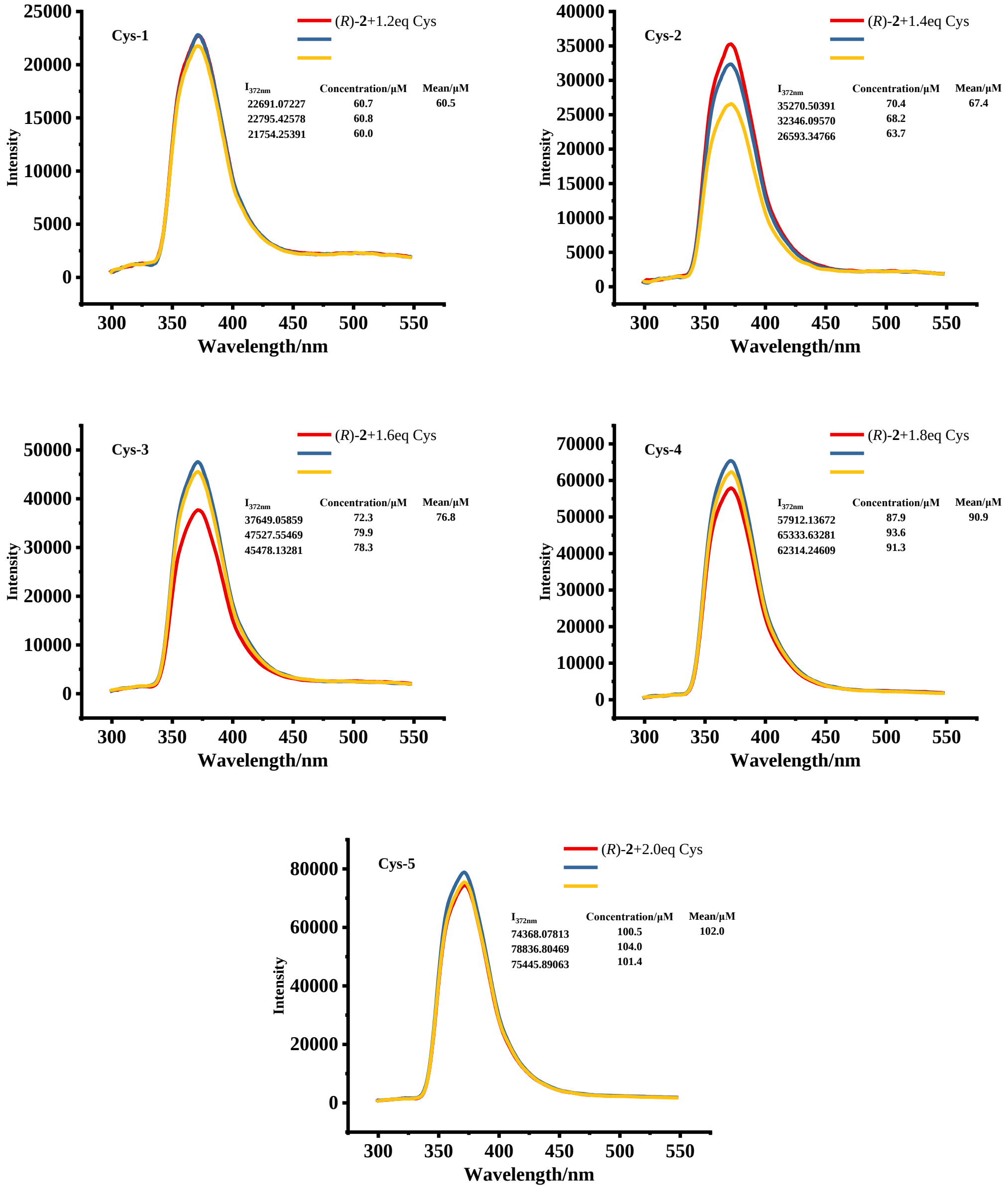

Table S1. Comparison of concentration of the control samples and determined by fluorescence (FL).

Sample name	Concentration/μM (control)	Concentration/μM (FL)	Deviation/%
**Cys-1**	60	60.5	+0.8%
**Cys-2**	70	67.4	-3.7%
**Cys-3**	80	76.8	-4.0%
**Cys-4**	90	90.9	+1.0%
**Cys-5**	100	100.4	+0.4%

2.9. Determination of the concentration of cysteine in ternary mixtures of Cys, Hcy and Met.

Figure S12. The standard curve for the fluorescence response of (*R*)-**2** (5×10^{-3} M, 20 μL in CH_2Cl_2) and PEOH (2.0 mL) upon addition of *L*-Cys-TBA (24, 28, 32, 36, 40 μL of 5 mM stock solution in MeOH respectively), *L*-Hcy-TBA (1.0 equiv, 2.5×10^{-2} M, 4 μL in MeOH) and *L*-Met-TBA (1.0 equiv, 2.5×10^{-2} M, 4 μL in MeOH). Because of the different volumes of cysteine added, additional methanol was added to the test tube for a final total volume of 2068 μL (Solvent: 97:2:1 (v:v:v) PEOH/MeOH/CH_2Cl_2; λ_{exc} = 284 nm; slits, 10/10 nm; reaction time, 120 min at 25 °C; Error bars from three independent experiments).

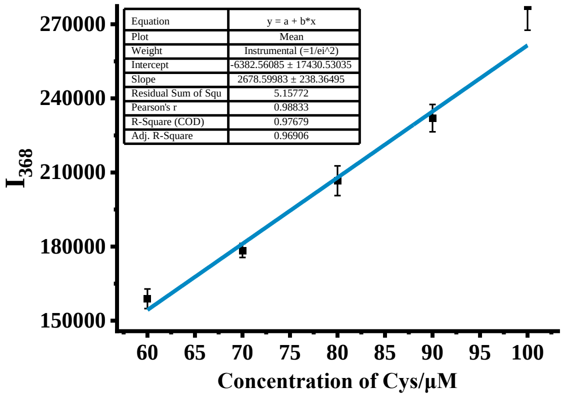

Figure S13. Fluorescence response of (*R*)-**2** (5×10^{-3} M, 20 μL in CH_2Cl_2) and PEOH (2.0 mL) upon addition of *L*-Cys-TBA (22, 26, 30, 34 μL of 5 mM stock solution in MeOH respectively), *L*-Hcy-TBA (1.0 equiv, 2.5×10^{-2} M, 4 μL in MeOH) and *L*-Met-TBA (1.0 equiv, 2.5×10^{-2} M, 4 μL in MeOH). Because of the different volumes of cysteine added, additional methanol was added to the test tube for a final total volume of 2068 μL. The standard curve in **Figure S12.** was applied to determine the concentration of cysteine (Solvent: 97:2:1 (v:v:v) PEOH/MeOH/CH_2Cl_2; λ_{exc} = 284 nm; slits, 10/10 nm; reaction time, 120 min at 25 °C; all the plots were obtained from three independent experiments).

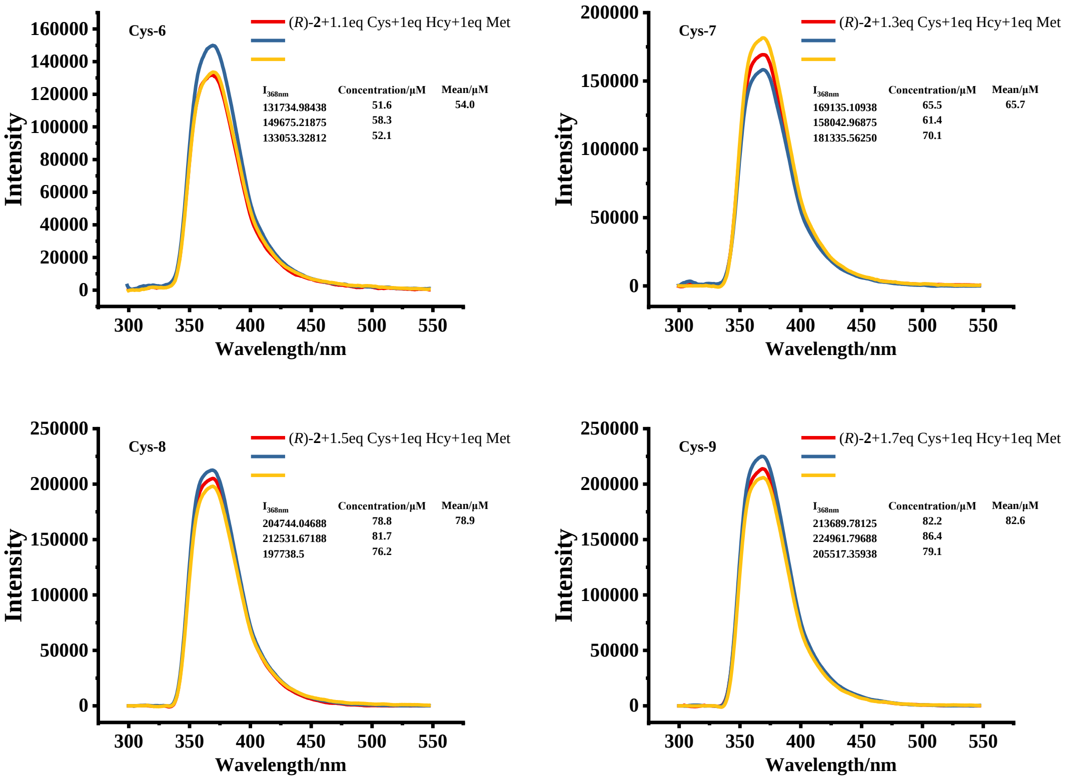

Table S2. Comparison of concentration of the control samples and determined by fluorescence (FL).

Sample name	Concentration/μM (control)	Concentration/μM (FL)	Deviation/%
**Cys-6**	55	54.0	-1.8%
**Cys-7**	65	65.7	+1.1%
**Cys-8**	75	78.9	+5.2%
**Cys-9**	85	82.6	-2.8%

2.10. Fluorescence spectra of (*R*)-2, (*R*)-7, (*R*)-8 with *L*-Cys-TBA and *L*-Cys-Na.

Figure S14. Fluorescence spectra of (*R*)-**2**, (*R*)-**7**, (*R*)**-8** (5.0 × 10^{-3} M, 5 μL in CH_2Cl_2) with *L*-Cys-TBA and *L*-Cys-Na (16.0 equiv, 5.0 × 10^{-2} M, 8 μL in MeOH) in PEOH (0.5 mL) respectively (solvent: 98:1:1 (v:v:v) PEOH/MeOH/CH_2Cl_2; λ_{exc} = 284 nm; slits, 5/5 nm; temperature, 25°C).

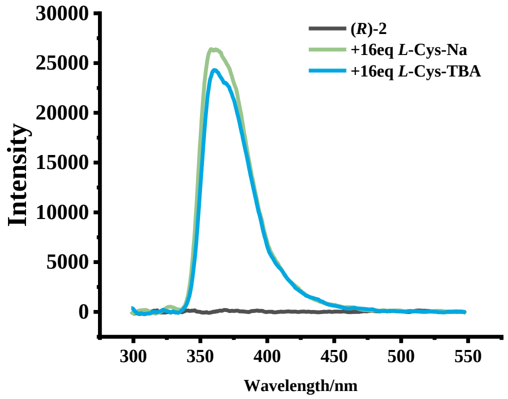

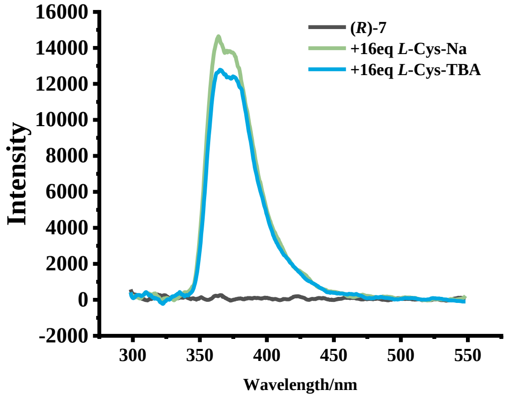

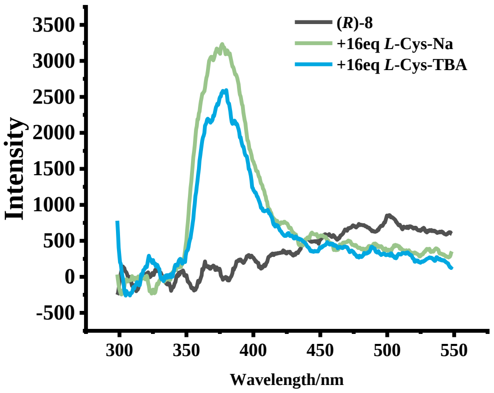

2.11. Fluorescence spectra of (*R*)-2 with *L*-Cys-TBA, N-Boc-*L*-Cys-TBA, N-Acetyl-*L*-Cys, S-Carboxymethyl-*L*-Cys and 2-Amino-ethanethio.

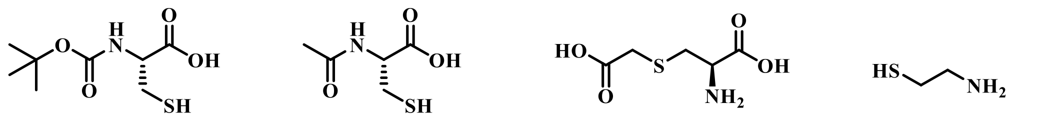

N-Boc-*L*-Cys   N-Acetyl-*L*-Cys   S-Carboxymethyl-*L*-Cys   2-Amino-ethanethio

Figure S15. Fluorescence spectra of (*R*)-**2** (5.0×10^{-3} M, 5 μL in CH_2Cl_2) with *L*-Cys-TBA, N-Boc-*L*-Cys-TBA, N-Acetyl-*L*-Cys, S-Carboxymethyl-*L*-Cys and 2-Amino-ethanethio (16.0 equiv, 5.0×10^{-2} M, 8 μL in MeOH) in PEOH (0.5 mL) respectively (solvent: 98:1:1 (v:v:v) PEOH/MeOH/CH_2Cl_2; λ_{exc} = 284 nm; slits, 5/5 nm; temperature, 25°C).

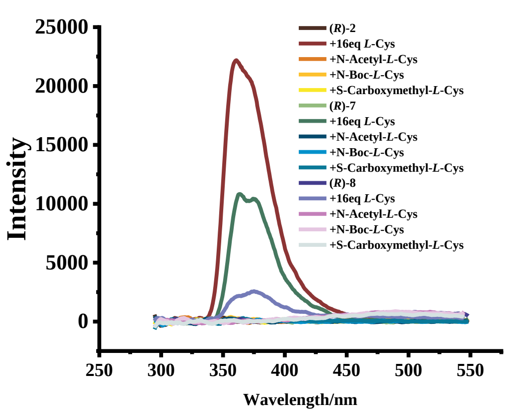

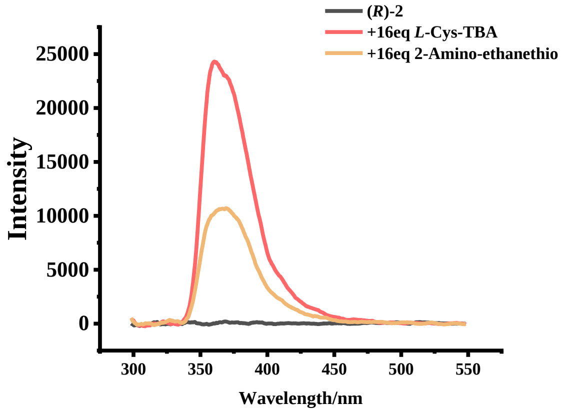

2.12. Fluorescence spectra of (*R*)-2 in the fluorous phase (PEOH) with Cysteine and the other biothiols.

Figure S16. Fluorescence intensity at λ = 360 nm, I_{360}, for the interaction of (*R*)-**2** (5.0×10^{-3} M, 5 μL in CH_2Cl_2) with Cys-TBA, Hcy-TBA, GSH-TBA, Met-TBA, HS^- and SCN^- (16.0 equiv, 5.0×10^{-2} M, 8 μL in MeOH) in PEOH (0.5 mL) (Solvent: 98:1:1 (v:v:v) PEOH/MeOH/CH_2Cl_2; λ_{exc} = 284 nm; slits, 5/5 nm; reaction time, 120 min at 25 °C; Error bars from three independent experiments).

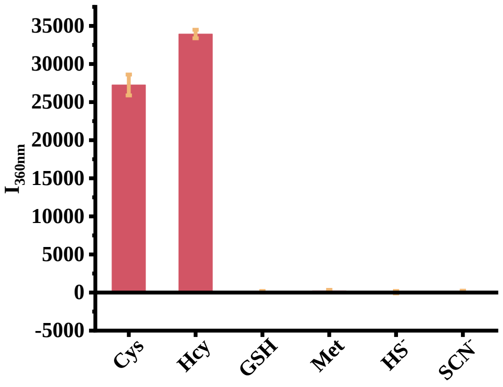

2.13. Influence of other biothiols and amino acids on cysteine.

Figure S17. Fluorescence intensity at λ = 360 nm, I_{360}, for the interaction of (*R*)-**2** (5.0 × 10^{-3} M, 5 μL in CH_2Cl_2) + Cys-TBA (16.0 equiv, 5.0 × 10^{-2} M, 8 μL in MeOH) with other biothiols (Hcy-TBA, GSH-TBA, HS^- and SCN^-) (16.0 equiv, 5.0 × 10^{-2} M, 8 μL in MeOH respectively) in PEOH (0.5 mL) (Solvent: 96:3:1 (v:v:v) PEOH/MeOH/CH_2Cl_2; λ_{exc} = 284 nm; slits, 5/5 nm; reaction time, 120 min at 25 °C; Error bars from three independent experiments).

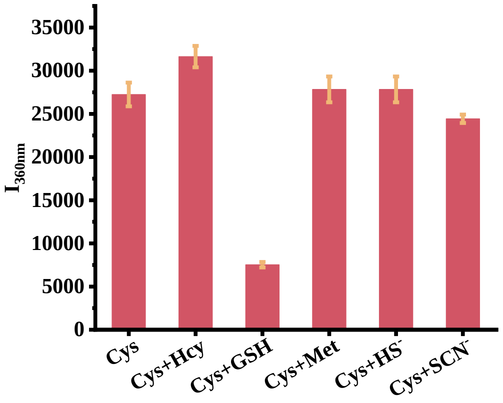

Figure S18. Fluorescence spectra of (*R*)-**2** (5.0 × 10^{-3} M, 5 μL in CH_2Cl_2) + Cys-TBA (16.0 equiv, 5.0 × 10^{-2} M, 8 μL in MeOH) with Hcy-TBA (16.0 equiv, 5.0 × 10^{-2} M, 8 μL in MeOH), GSH-TBA (16.0 equiv, 5.0 × 10^{-2} M, 8 μL in MeOH) and Hcy-TBA + GSH-TBA respectively in PEOH (0.5 mL) (solvent: (v:v:v) PEOH/MeOH/CH_2Cl_2; λ_{exc} = 284 nm; slits, 5/5 nm; reaction time, 120 min at 25 °C; Error bars from three independent experiments).

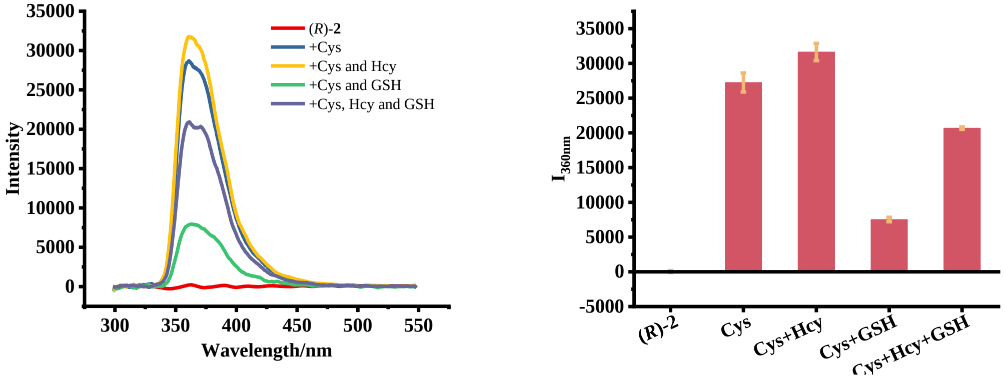

Figure S19. Fluorescent intensity at 360 nm of (*R*)-**2** (5.0×10^{-3} M, 5 μL in CH_2Cl_2) + Cys-TBA (16.0 equiv, 5.0×10^{-2} M, 8 μL in MeOH) versus the equivalent of GSH-TBA (0, 2, 4, 6, 8, 10, 12, 14 and 16 equiv, 5.0×10^{-2} M, 0, 1, 2, 3, 4, 5, 6, 7 and 8 μL in MeOH respectively). Because of the different volumes of cysteine added, additional methanol was added to the test tube for a final total volume of 521 μL. (solvent: 96:3:1 (v:v:v) PEOH/MeOH/CH_2Cl_2; λ_{exc} = 284 nm; slits, 5/5 nm; reaction time, 120 min at 25 °C; Error bars from three independent experiments).

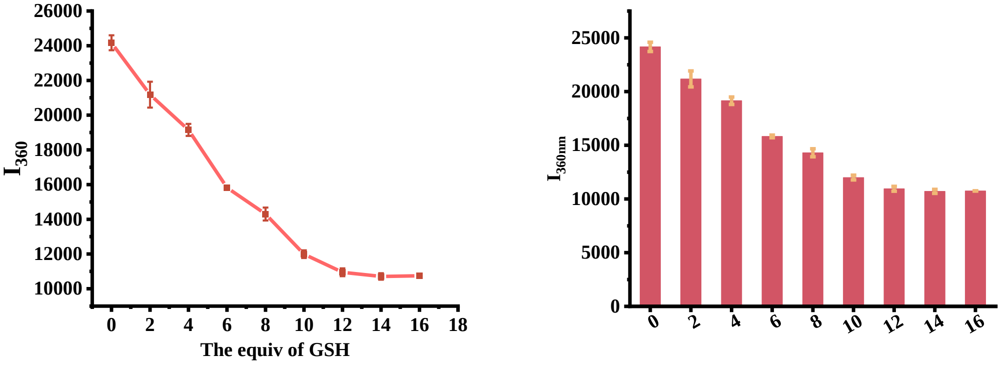

Figure S20. Fluorescence intensity at λ = 360 nm, I_{360}, for the interaction of (*R*)-**2** (5.0×10^{-3} M, 5 μL in CH_2Cl_2) + Cys (16.0 equiv, 5.0×10^{-2} M, 8 μL in MeOH) with other 19 amino aicds (16.0 equiv, 5.0×10^{-2} M, 8 μL in MeOH) in PEOH (0.5 mL) (Solvent: 96:3:1 (v:v:v) PEOH/MeOH/CH_2Cl_2; λ_{exc} = 284 nm; slits, 5/5 nm; reaction time, 120 min at 25 °C; Error bars from three independent experiments).

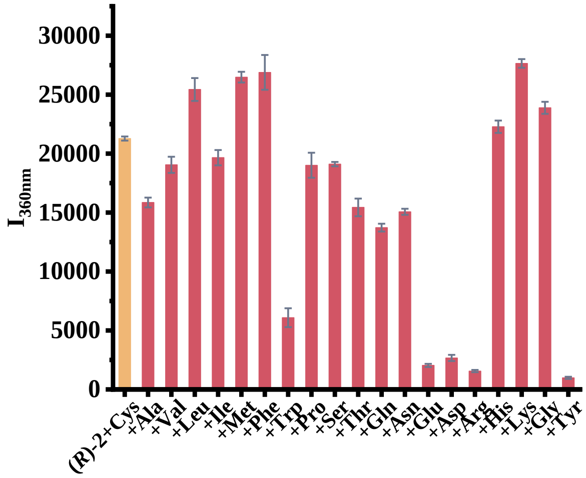

2.14. Fluorescence spectra of (*S*)-2 with 1.0-20.0 equivalent *L*-Hcy-TBA.

Figure S21. Fluorescence spectra of (*S*)-**2** (5.0 × 10^{-3} M, 5 μL in CH_2Cl_2) with 1.0 - 10.0 equivalent *L*-Hcy-TBA (5, 10, 15, 20, 25, 30, 35, 40, 45, 50 μL of 5.0 × 10^{-3} M stock solution in MeOH) and 12.0 - 20.0 equivalent *L*-Hcy-TBA (6, 7, 8, 9, 10 μL of 5.0 × 10^{-2} M stock solution in MeOH) in PEOH (0.5 mL). Additional methanol was added to the test tube to give a final volume of 555 μL (Solvent: PEOH/MeOH/CH_2Cl_2 = 90:9:1, v/v/v. λ_{exc} = 284 nm; slits = 5/5 nm; reaction time: 120 min; temperature: 25 °C).

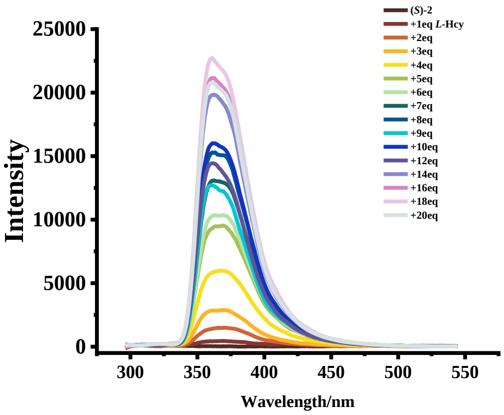

2.15. Fluorescence spectra of compound 4', compound 5, compound 5', and compound 13.

Figure S22. Fluorescence spectra of compound **5** (5.0 × 10^{-5} M) and compound **5'** (5.0 × 10^{-5} M) in MeOH, compound **4'** (5.0 × 10^{-5} M) and compound **13** (5.0 × 10^{-5} M) in PEOH/MeOH = 99:1, v/v, respectively (λ_{exc} = 284 nm; slits, 5/5 nm; temperature, 25°C).

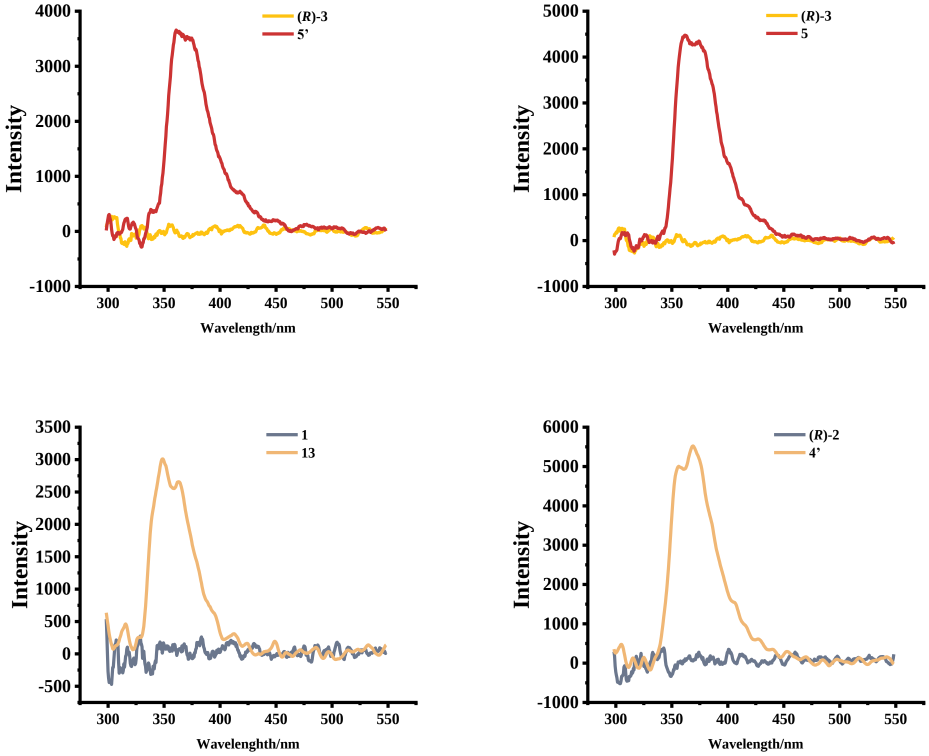

2.16. Fluorescence spectra of compound 5’ with 18 metal ions in MeOH.

Figure S23. Fluorescence intensity at λ = 370 nm, I_{370}/I_0, for the interaction of compound **5’** (5.0×10^{-5} M) with 18 metal ions (2.0 equiv) in MeOH (λ_{exc} = 284 nm; slits = 5/5 nm; reaction time: 120 min; temperature: 25 °C; I_0: fluorescence intensity of compound **5’** at 370 nm without metal ions).

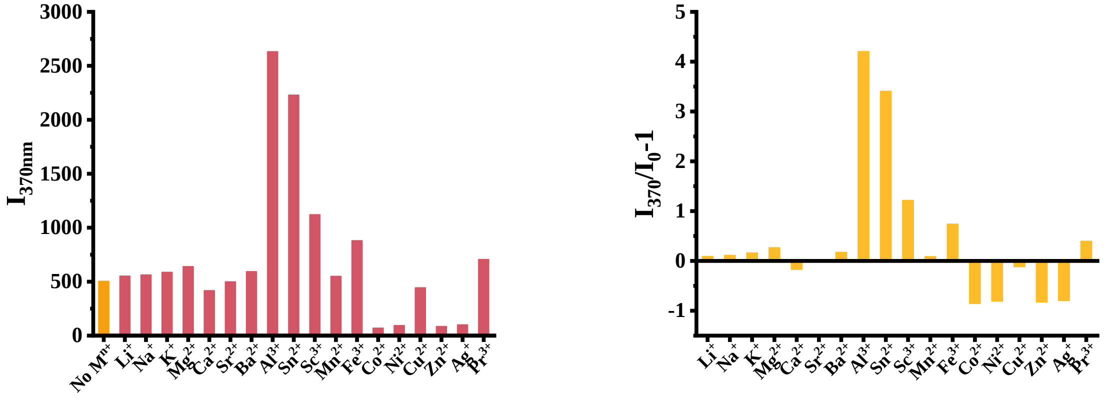

2.17. Fluorescence spectra of compound 5 with 18 metal ions in MeOH.

Figure S24. Fluorescence intensity at λ = 370 nm, I_{370}/I_0, for the interaction of compound **5** (5.0×10^{-5} M) with 18 metal ions (2.0 equiv) in MeOH (λ_{exc} = 284 nm; slits = 5/5 nm; reaction time: 120 min; temperature: 25 °C; I_0: fluorescence intensity of compound **5** at 370 nm without metal ions).

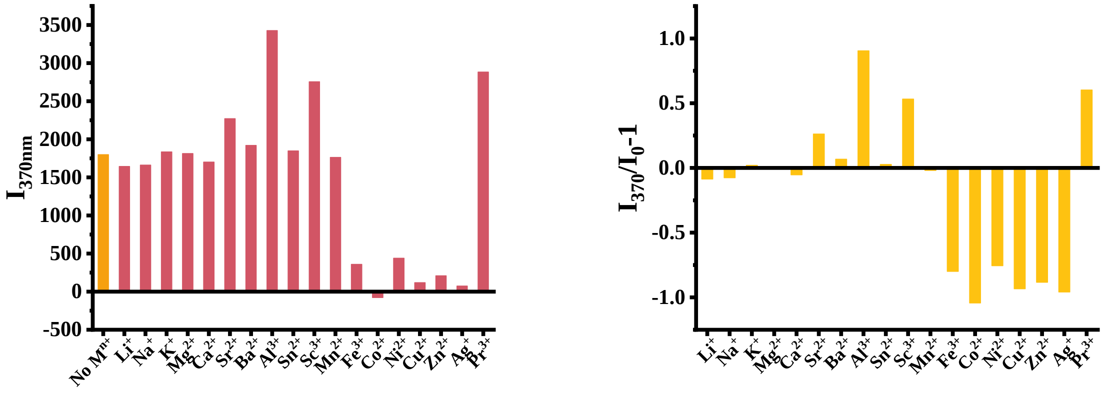

2.18. Fluorescence spectra of compound 14 with 20 metal ions in PEOH.

Figure S25. Fluorescence intensity at λ = 350 nm, I_{350}, for the interaction of compound **14** (5.0×10^{-5} M) with 20 metal ions (2.0 equiv) in PEOH (λ_{exc} = 284 nm; slits = 5/5 nm; reaction time: 120 min; temperature: 25 °C; I_0: fluorescence intensity of compound **14** at 350 nm without metal ions).

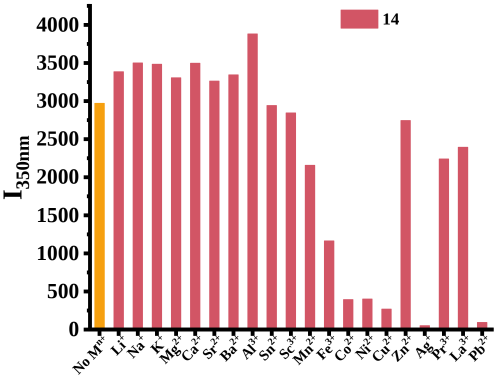

2.19. Fluorescence spectra of (*R*)-2 in the fluorous phase (PEOH) with cysteine in water.

Figure S26. (a) Fluorescence spectra of (*R*)-**2** (5.0×10^{-3} M, 20 μL in CH_2Cl_2) in PEOH (2.0 mL) with *L*-Cys-TBA (16.0 equiv, 5.0×10^{-2} M, 8 μL in deionized water) in water (2.0 mL) under stirring, (b) Fluorescence spectra of (*R*)-**2** (5.0×10^{-3} M, 5 μL in CH_2Cl_2) with *L*-Cys-TBA (16.0 equiv, 5.0×10^{-2} M, 8 μL in deionized water) in PEOH (0.5 mL) after ultrasonic mixing. (λ_{exc} = 284 nm; slits, 5/5 nm; reaction time, 120 min at 25 °C; all the plots were obtained from three independent experiments).

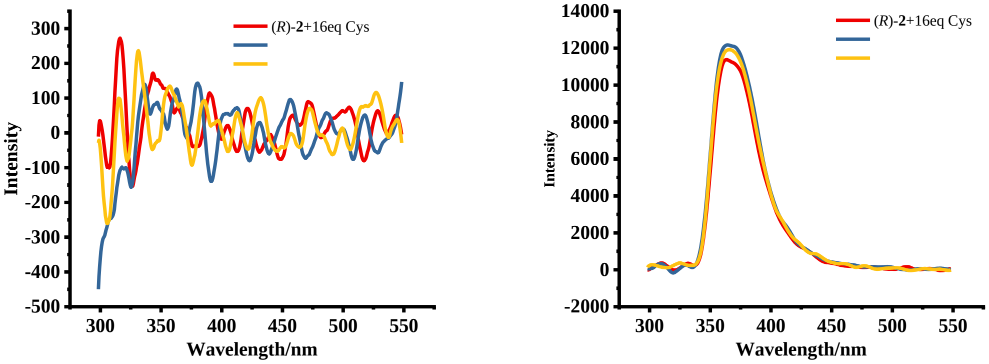

(a) (b)

3. NMR spectroscopic study.

3.1. NMR titration for the reaction of (*R*)-2, (*R*)-7 and (*R*)-8 with 0-10.0 equiv. *L*-Cys-TBA.

Figure S27. ^{1}H NMR spectra of (*R*)-**2** (2.0×10^{-2} M, 0.004 mmol in 200 μL $CDCl_3$) with *L*-Cys-TBA (0-10.0 equiv. 1.0×10^{-1} M in CD_3OD) ($CDCl_3/CD_3OD$ = 1/2, v/v, measured after 12 h reaction at room temperature).

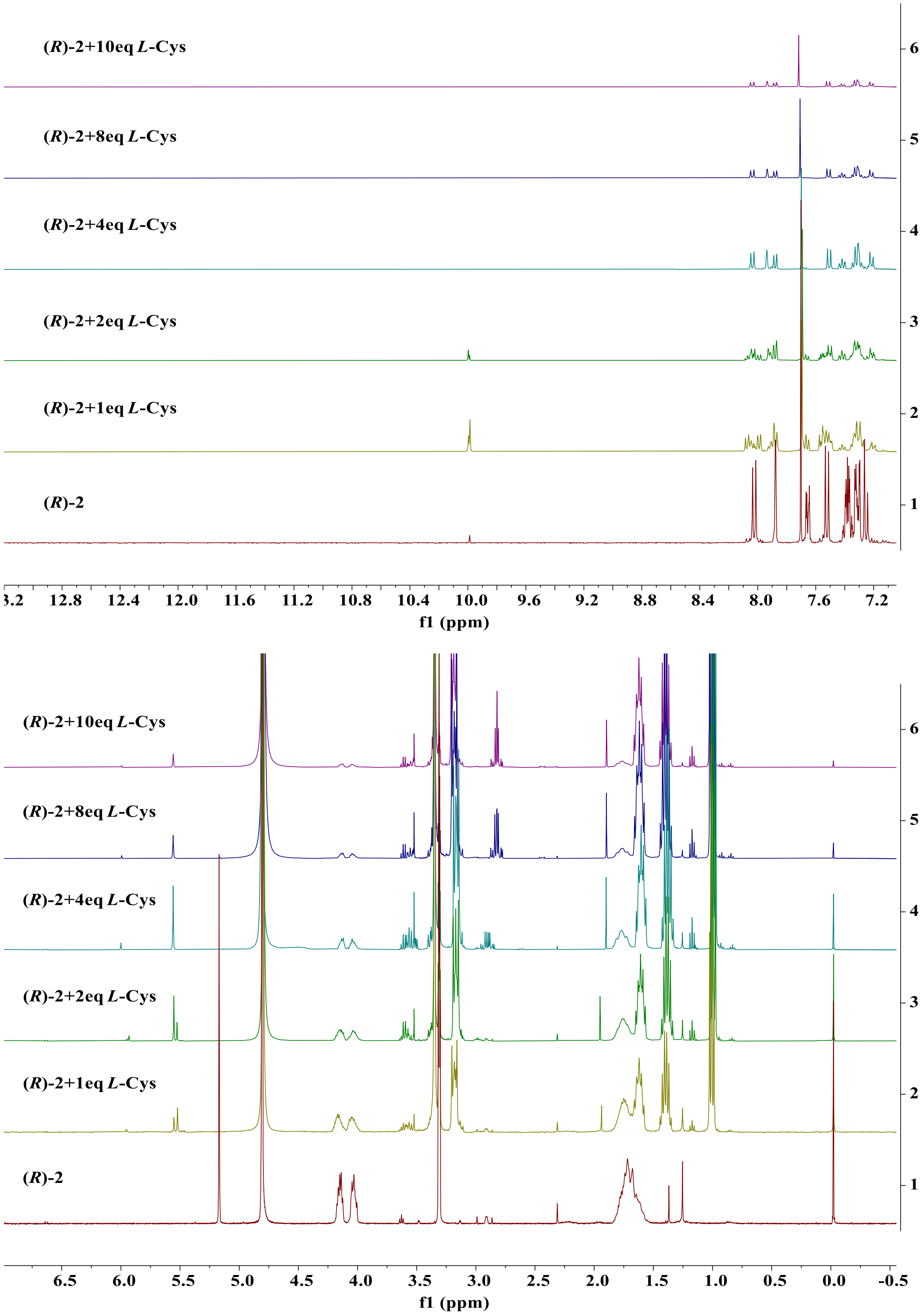

Figure S28. ^{1}H NMR spectra of (*R*)-**7** (2.0×10^{-2} M, 0.004 mmol in 200 μL $CDCl_3$) with *L*-Cys-TBA (0-10.0 equiv. 1.0×10^{-1} M in CD_3OD) ($CDCl_3/CD_3OD$ = 1/2, v/v, measured after 12 h reaction at room temperature).

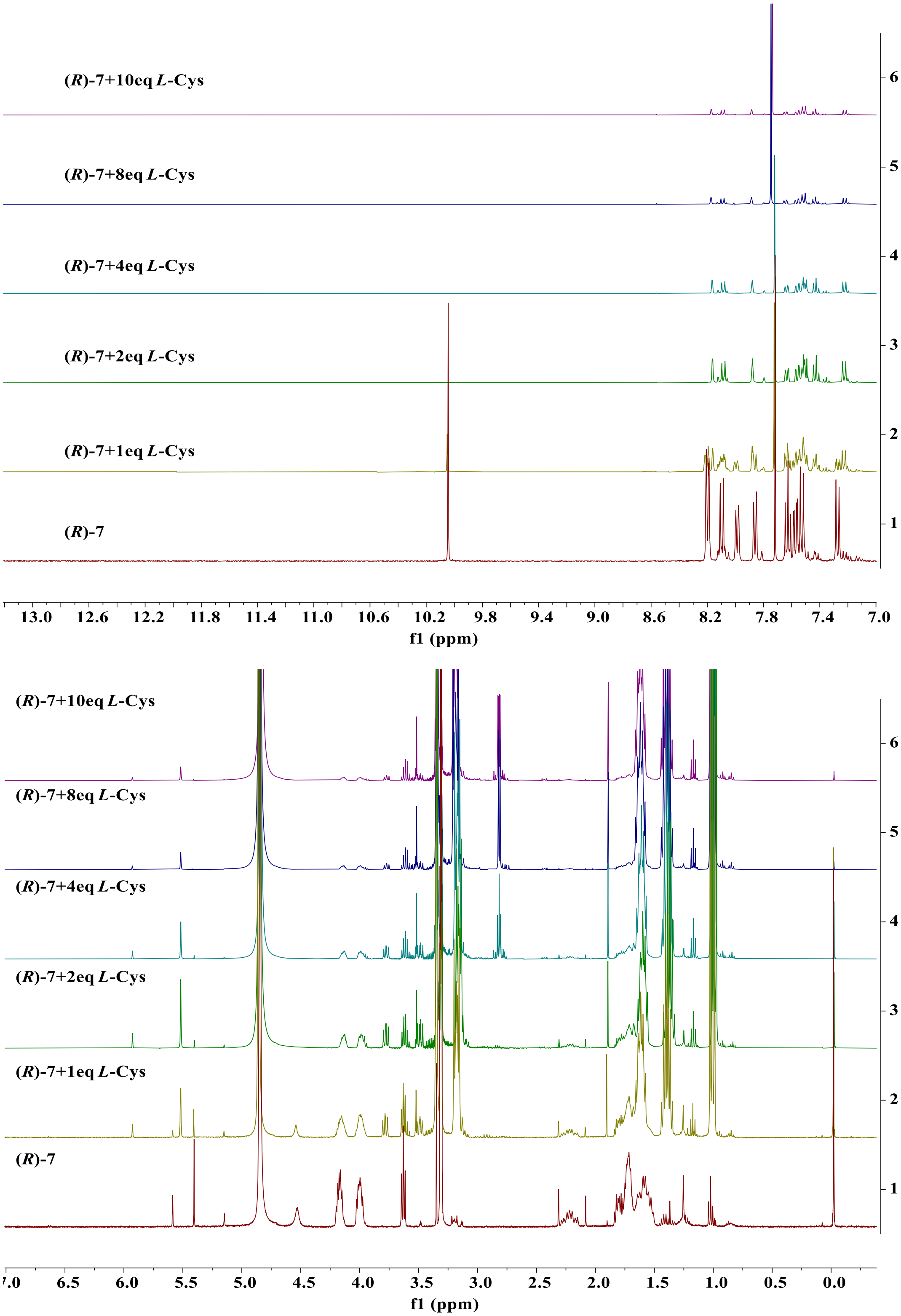

Figure S29. ^{1}H NMR spectra of (*R*)-**8** (2.0×10^{-2} M, 0.004 mmol in 200 μL $CDCl_3$) with *L*-Cys-TBA (0-10.0 equiv. 1.0×10^{-1} M in CD_3OD) ($CDCl_3/CD_3OD$ = 1/2, v/v, measured after 12 h reaction at room temperature).

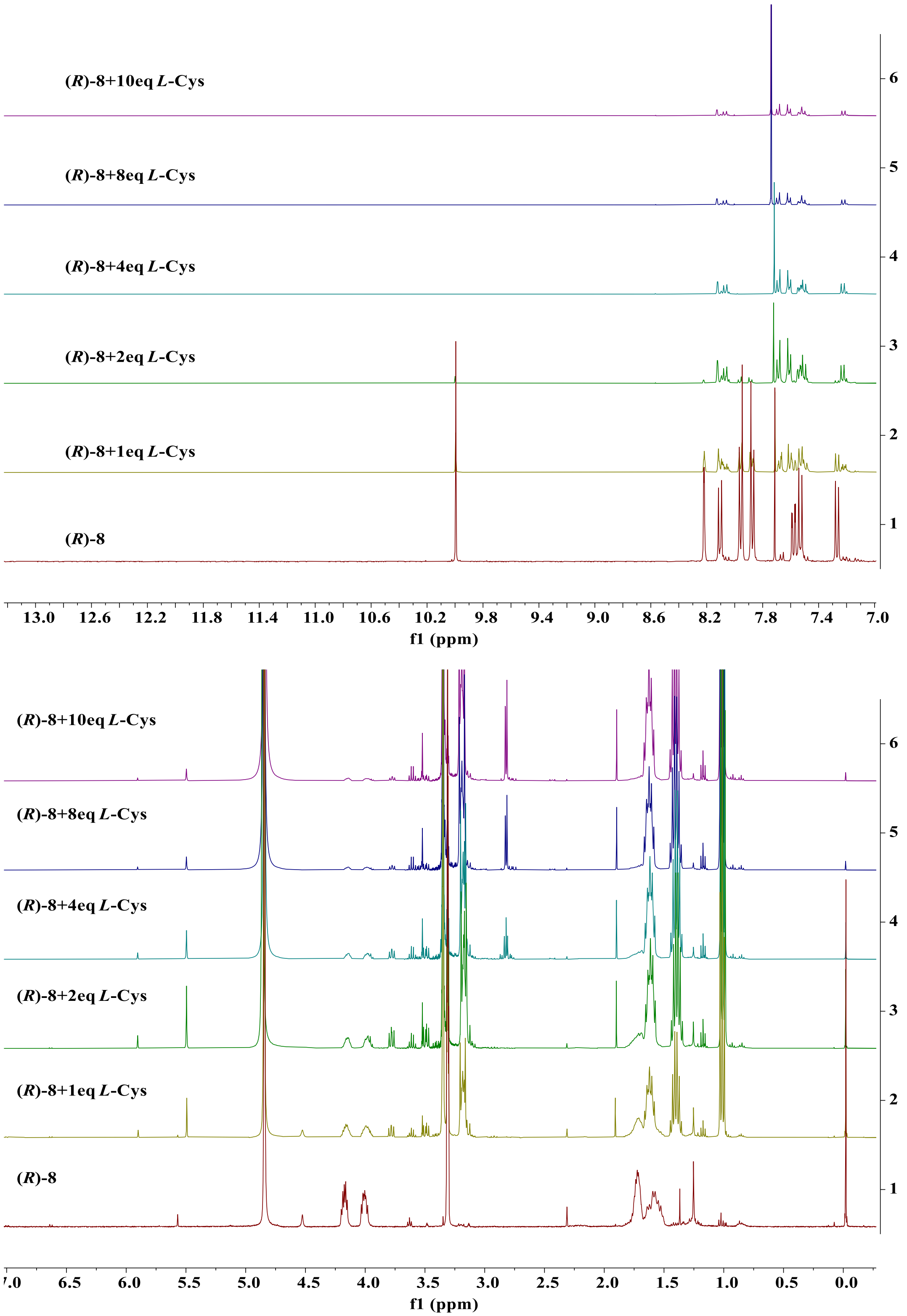

3.2. HSQC spectra of (*R*)-2, (*R*)-7 and (*R*)-8 with 4.0 equiv. *L*-Cys-TBA.

Figure S30. HSQC spectra of (*R*)-**2**, (*R*)-**7** and (*R*)-**8** (2.0×10^{-2} M, 0.004 mmol in 200 μL $CDCl_3$) with *L*-Cys-TBA (4.0 equiv. 1.0×10^{-1} M in CD_3OD) ($CDCl_3/CD_3OD$=1/2, v/v, measured after 12 h reaction at room temperature). (a) (*R*)-**2** (b) (*R*)-**7** (c) (*R*)-**8**

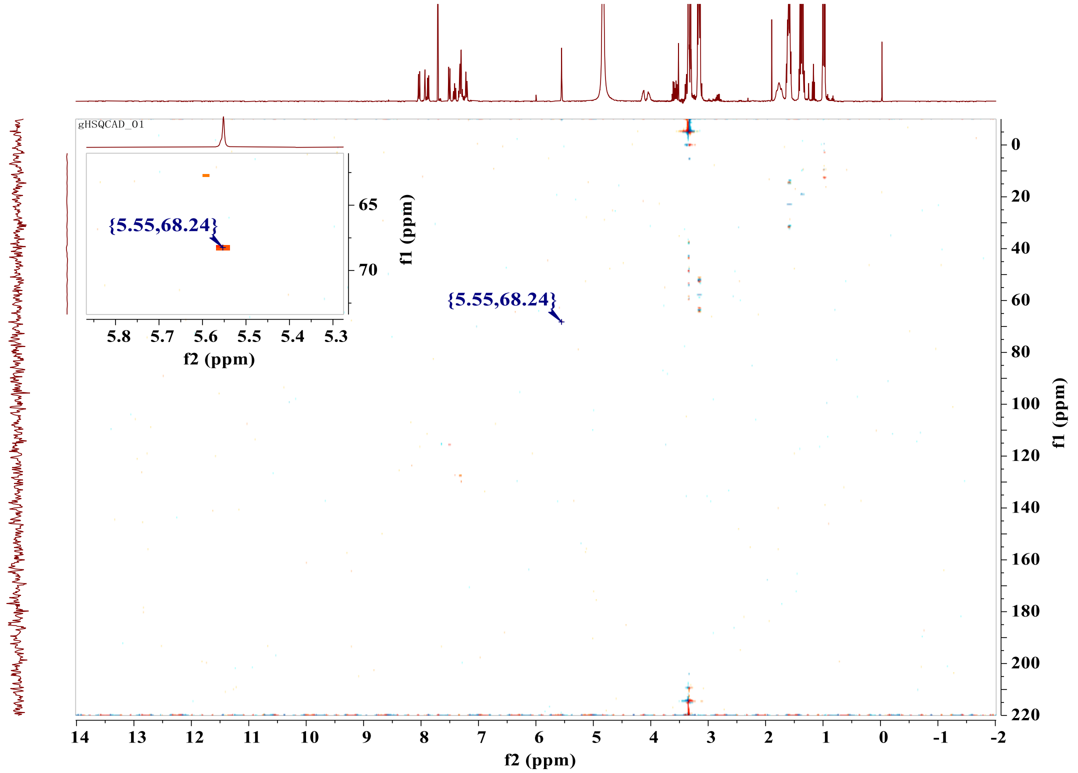

(a)

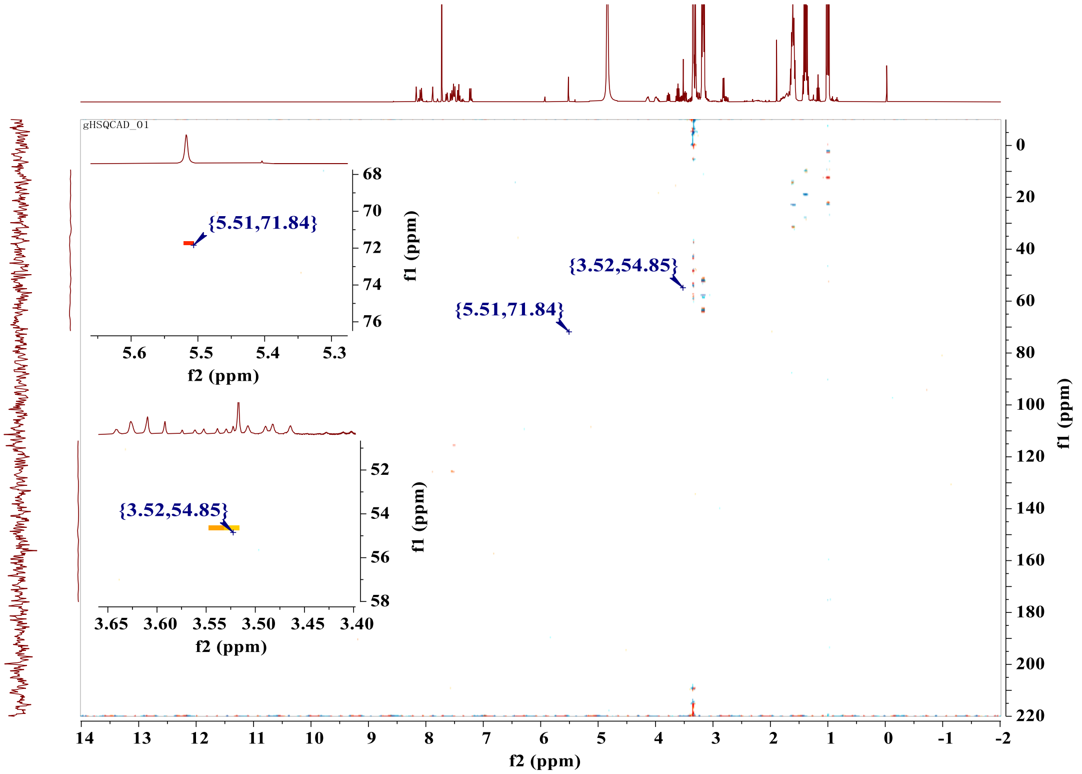

(b)

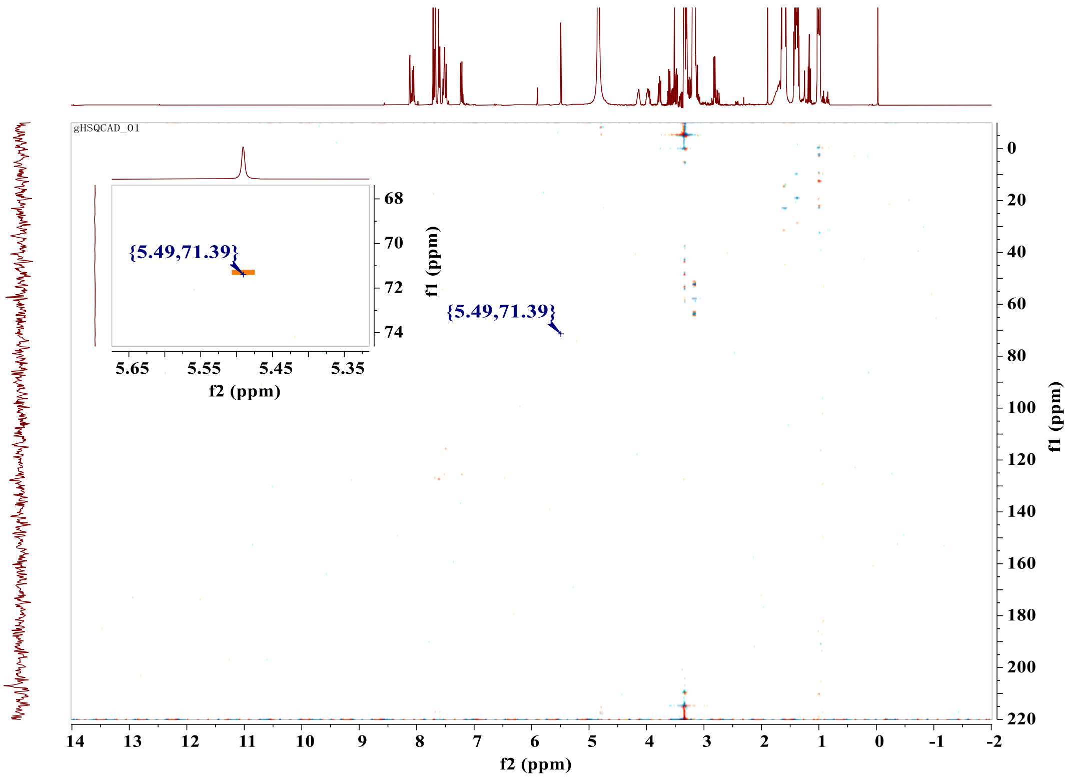

(c)

3.3. 2D NMR spectra of compound 4'

Figure S31. 2D NMR (400 MHz) spectra of compound **4'** (3.3×10^{-2} M, 0.02 mmol in 600 μL CD_3OD) (a)COSY (b)NOESY

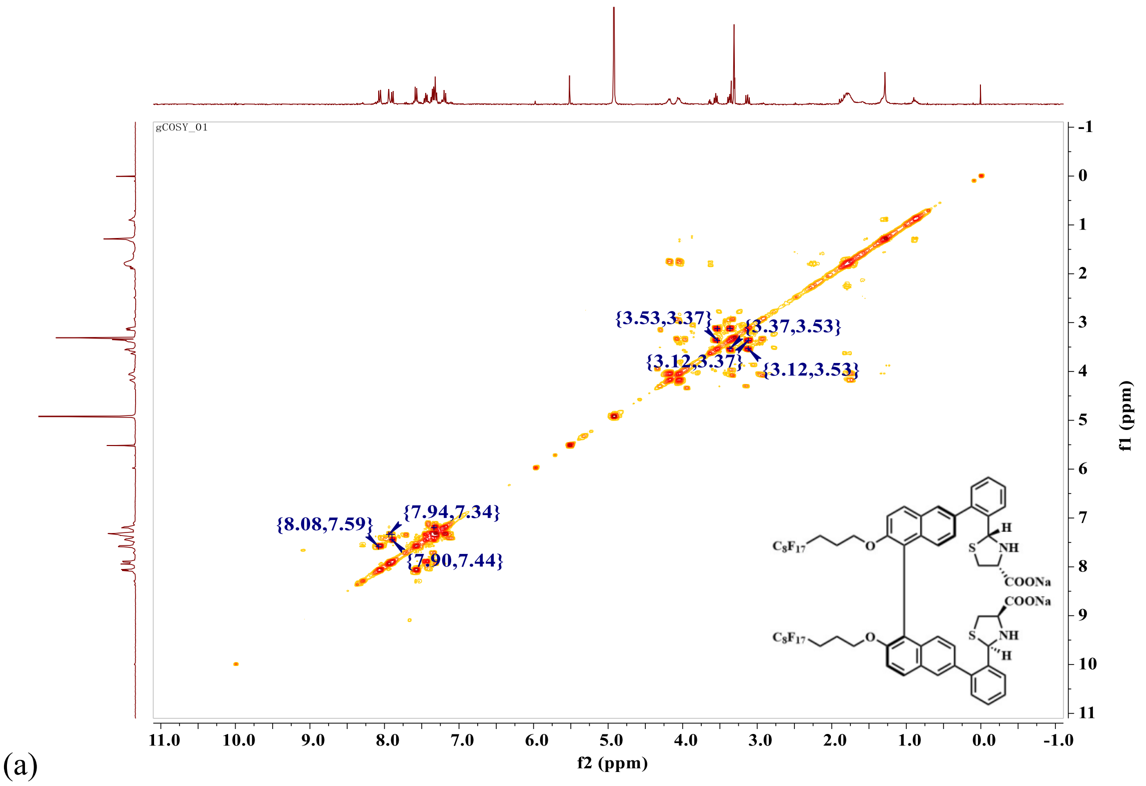

(a)

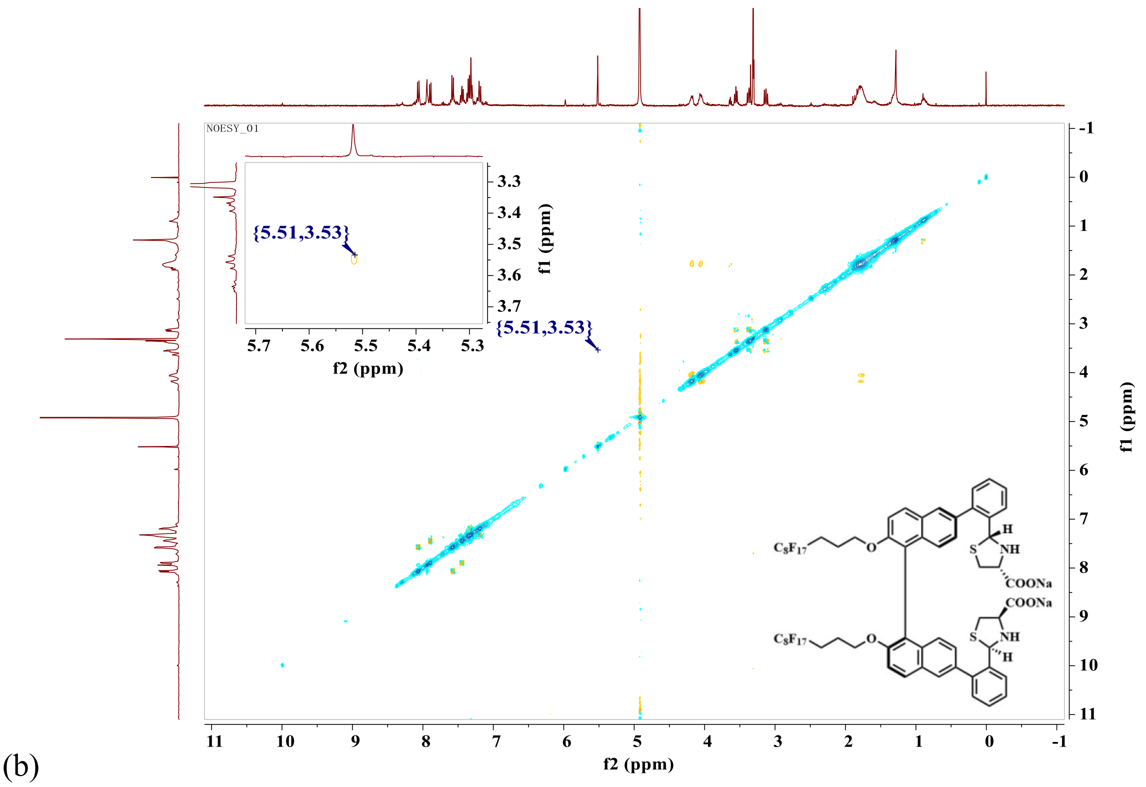

(b)

3.4. 2D NMR spectra of compound 5'.

Figure S32. 2D NMR (400 MHz) spectra of compound **5'** (3.3×10^{-2} M, 0.02 mmol in 600 μL CD_3OD). (a)HSQC (b)HMBC (c)COSY (d)NOESY

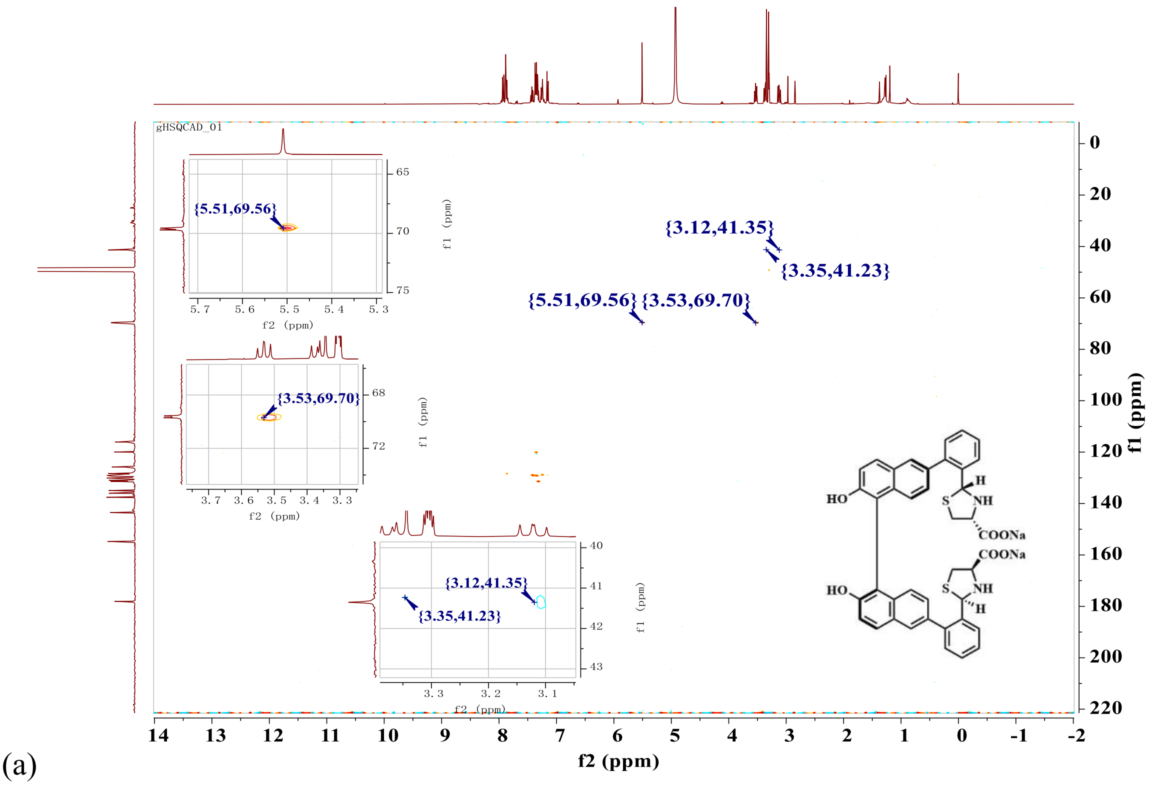

(a)

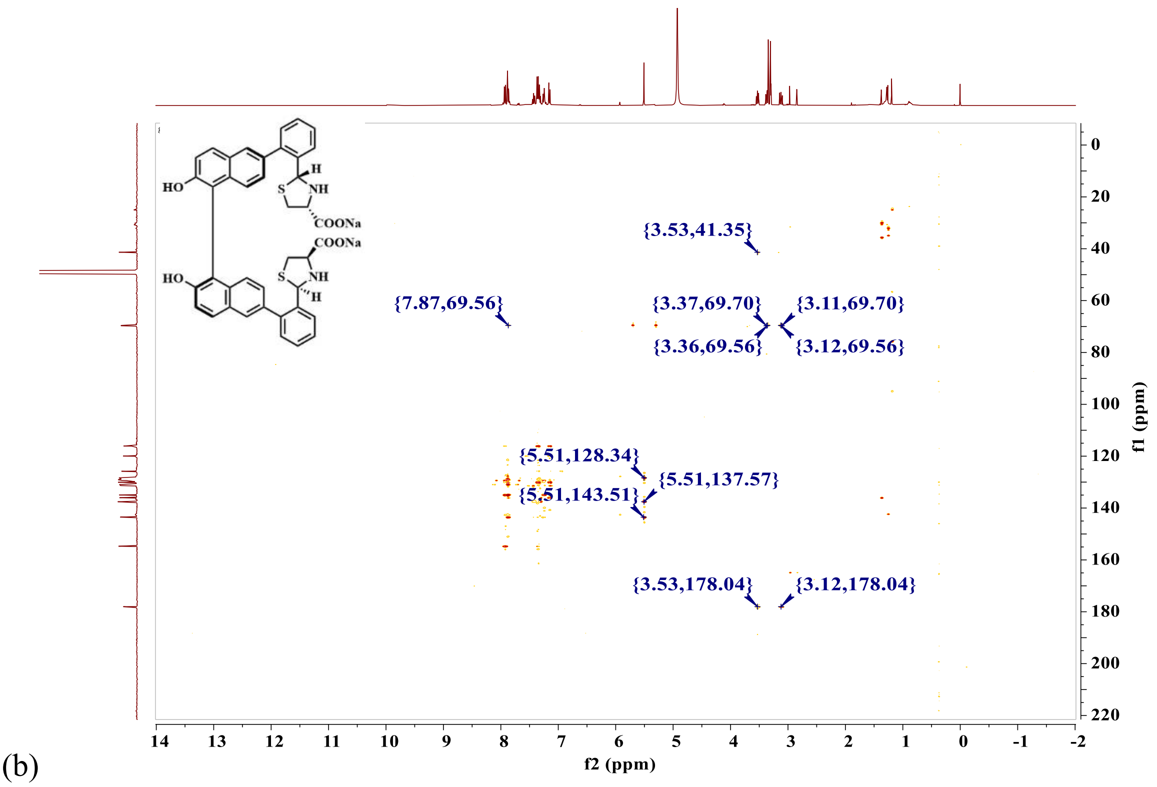
HO
H
S
NH
COONa
COONa
HO
S
NH
H
{3.53,41.35}
{7.87,69.56}
{3.37,69.70}
{3.11,69.70}
{3.36,69.56}
{3.12,69.56}
{5.51,128.34}
{5.51,137.57}
{5.51,143.51}
{3.53,178.04}
{3.12,178.04}
f1 (ppm)
f2 (ppm)

(b)

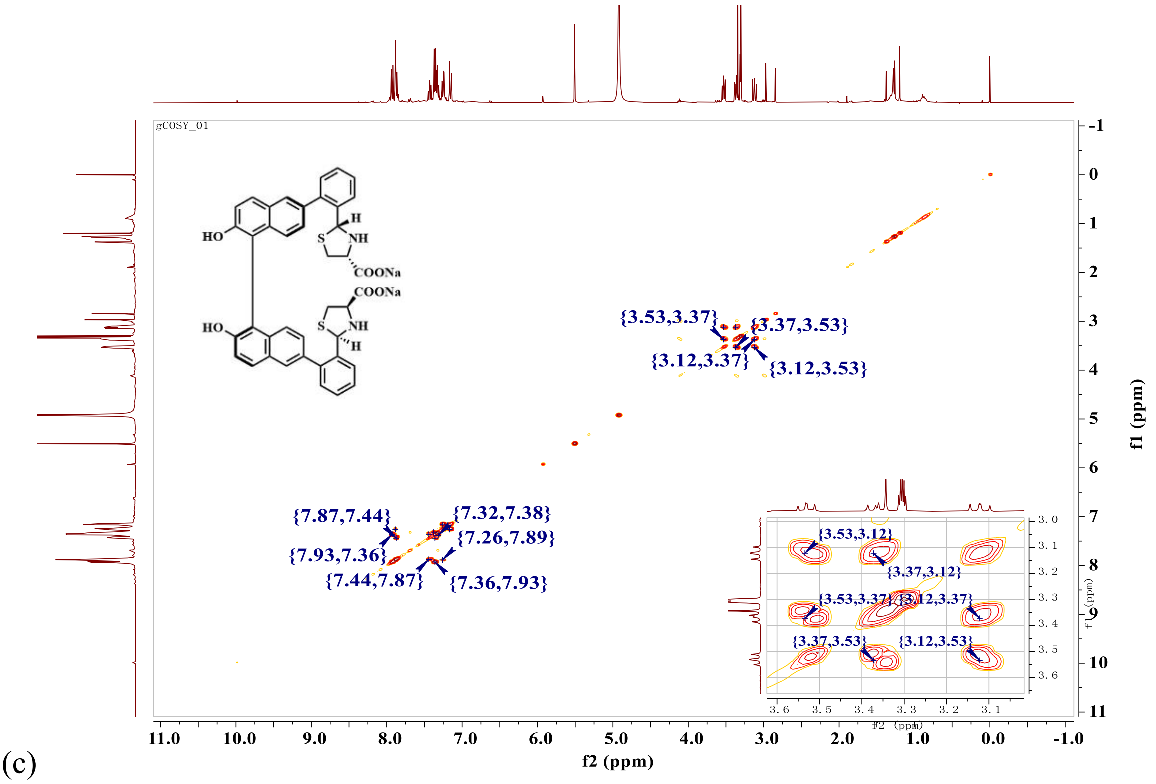
gCOSY_01
HO
H
S
NH
COONa
COONa
HO
S
NH
H
{3.53,3.37}
{3.37,3.53}
{3.12,3.37}
{3.12,3.53}
{7.87,7.44}
{7.32,7.38}
{7.26,7.89}
{7.93,7.36}
{7.44,7.87}
{7.36,7.93}
{3.53,3.12}
{3.37,3.12}
{3.53,3.37}
{3.12,3.37}
{3.37,3.53}
{3.12,3.53}
f1 (ppm)
f2 (ppm)

(c)

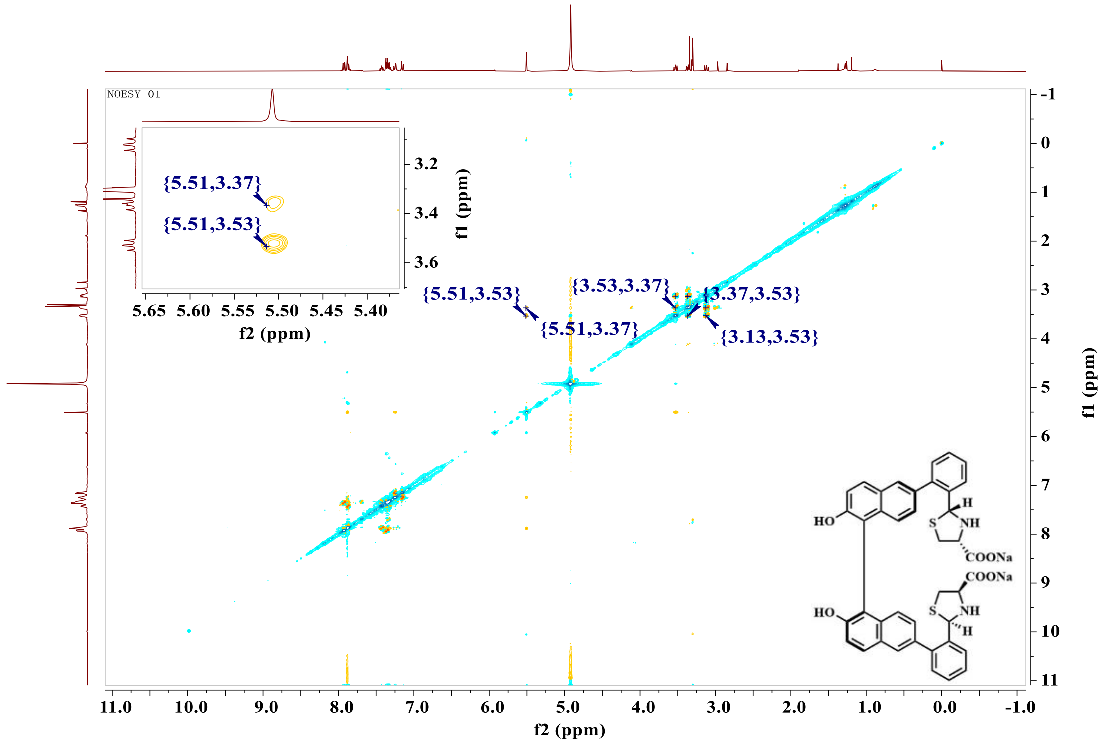

(d)

3.5. 2D NMR spectra of compound 5.

Figure S33. 2D NMR (400 MHz) spectra of compound **5** (3.3×10^{-2} M, 0.02 mmol in 600 μL CD_3OD) (a)HSQC (b)HMBC (c)COSY (d)NOESY

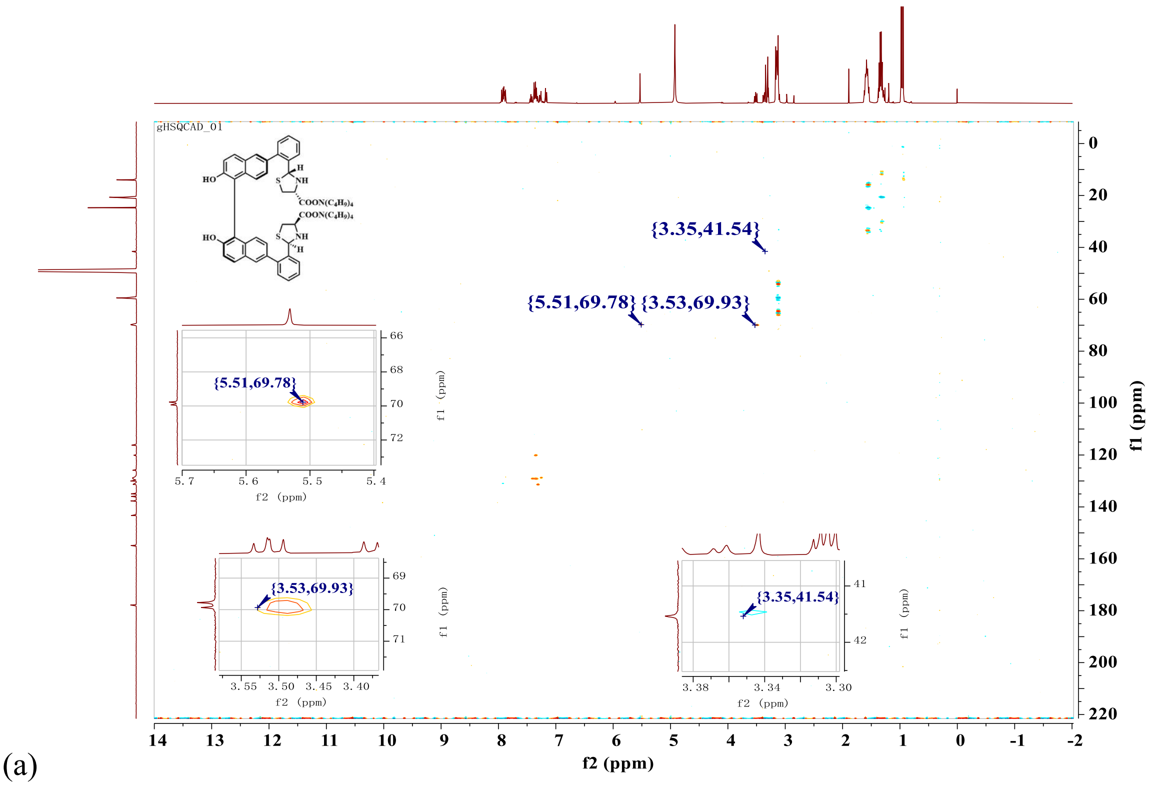

(a)

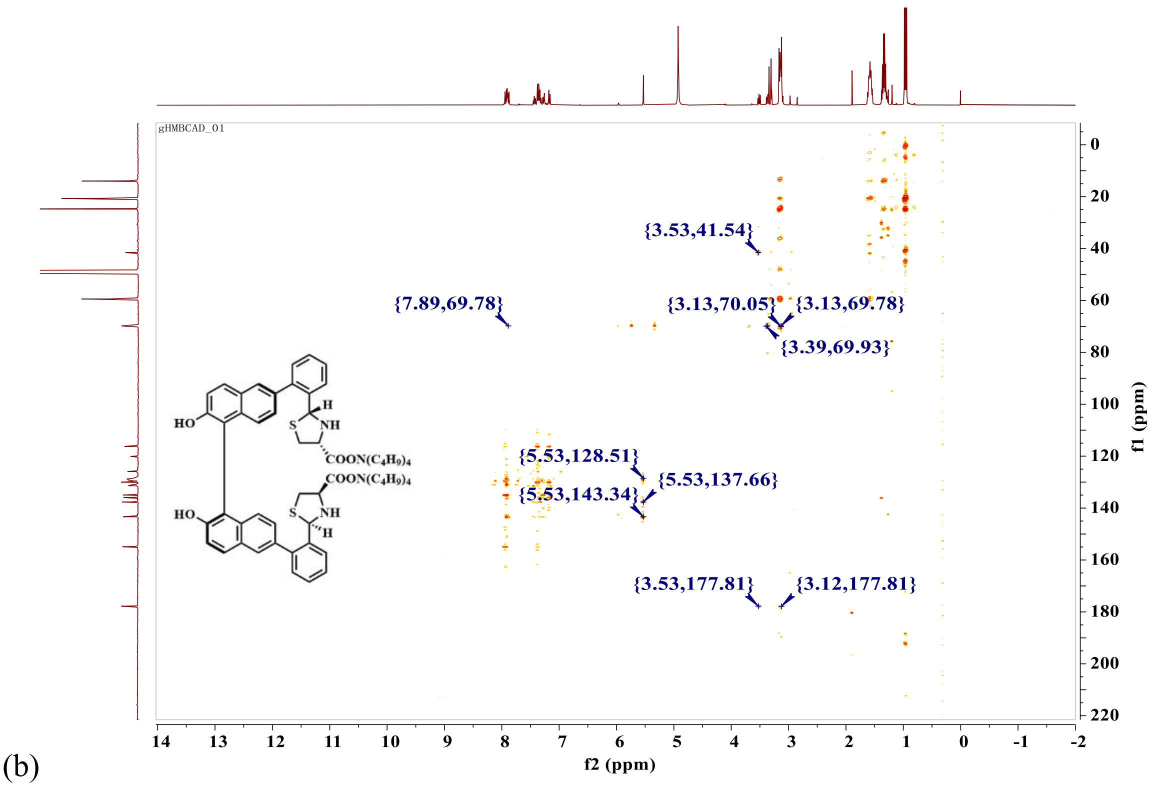
gHMBCAD_01
{3.53,41.54}
{7.89,69.78}
{3.13,70.05}
{3.13,69.78}
{3.39,69.93}
{5.53,128.51}
{5.53,137.66}
{5.53,143.34}
{3.53,177.81}
{3.12,177.81}
HO
HO
NH
S
H
COON(C4H9)4
f1 (ppm)
f2 (ppm)

(b)

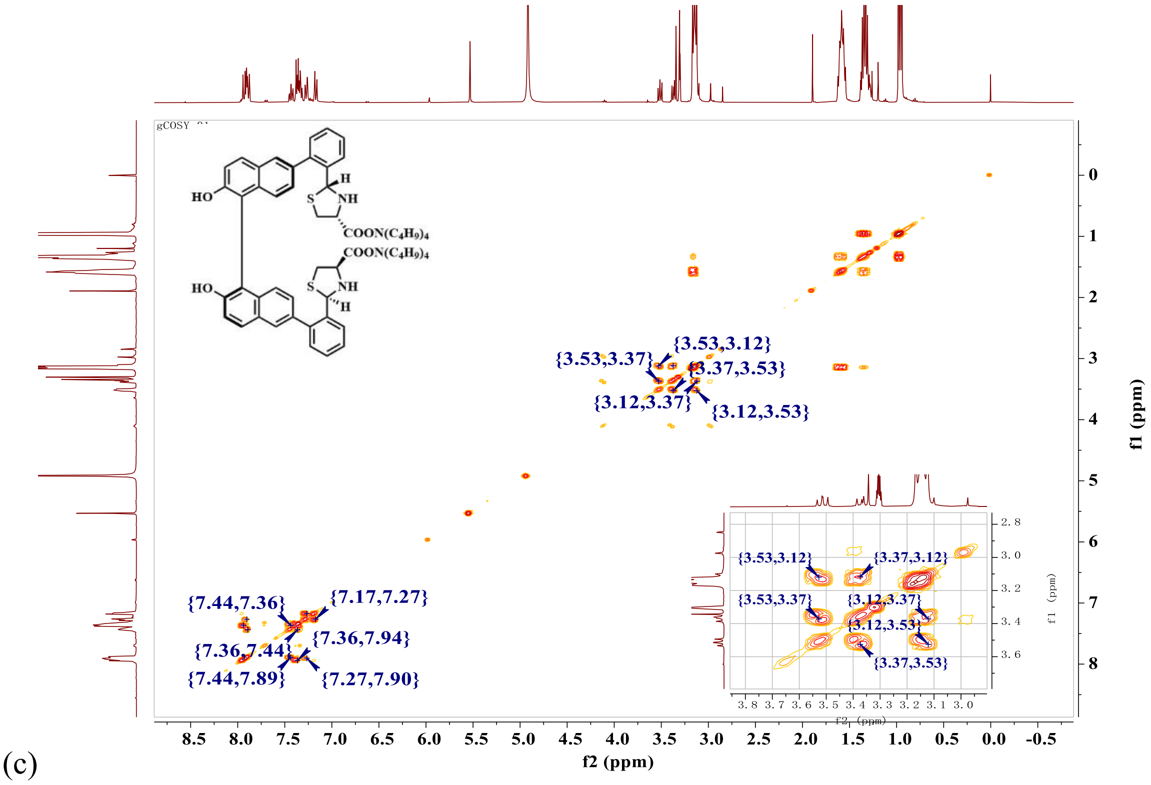
gCOSY
{3.53,3.12}
{3.53,3.37}
{3.37,3.53}
{3.12,3.37}
{3.12,3.53}
{7.44,7.36}
{7.17,7.27}
{7.36,7.94}
{7.36,7.44}
{7.44,7.89}
{7.27,7.90}
{3.37,3.12}
HO
HO
NH
S
H
COON(C4H9)4
f1 (ppm)
f2 (ppm)

(c)

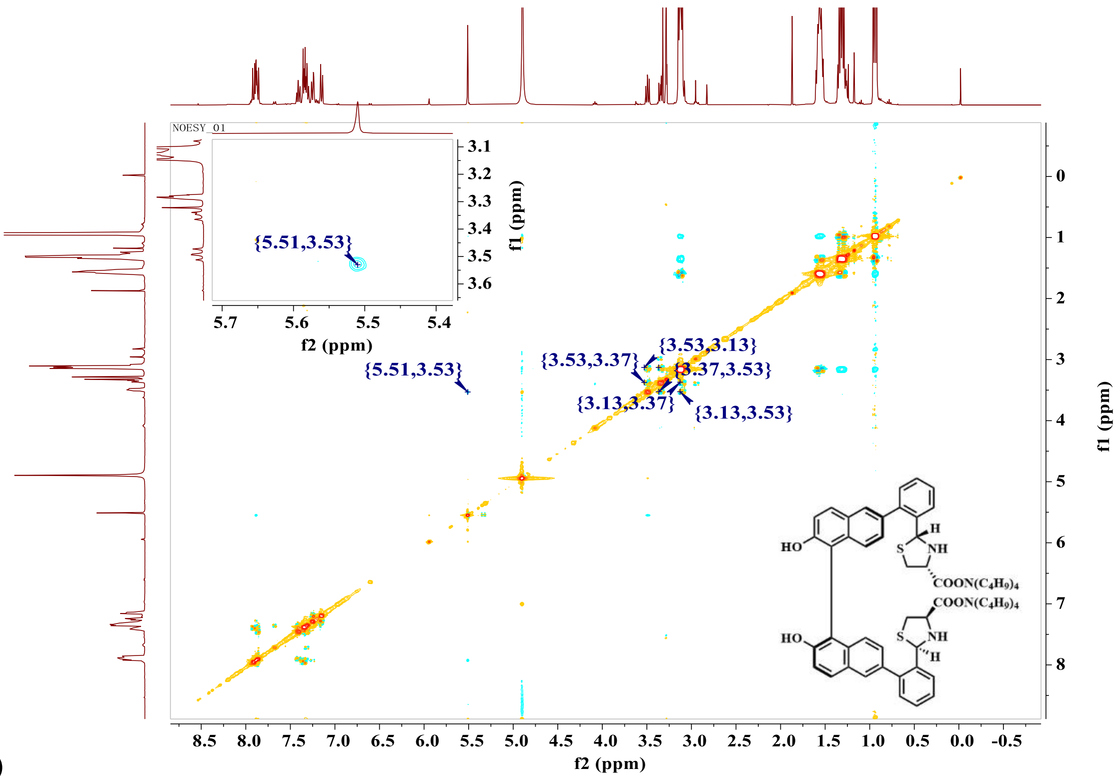

(d)

3.6. NOESY spectrum of compound 14

Figure S34. NOESY spectrum (400 MHz) of compound **14** (3.0×10^{-2} M) in CD_3OD.

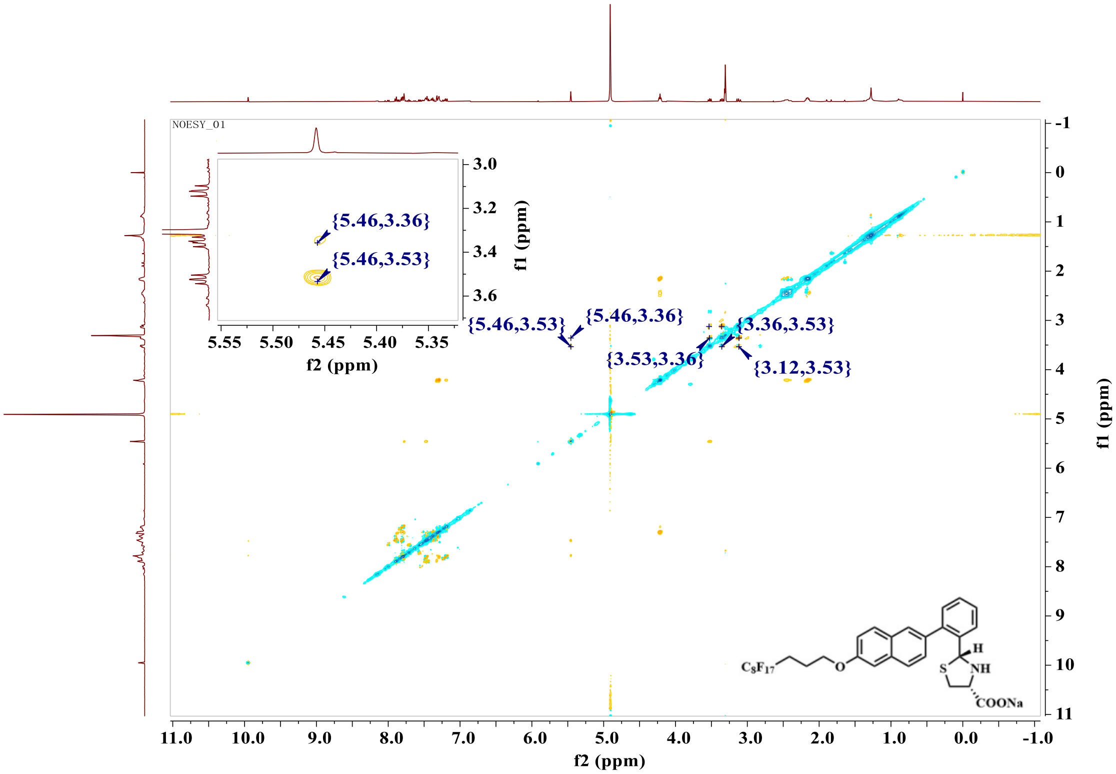

4. Mass spectra.

4.1. Mass spectrum for the reaction of (*R*)-2 with *L*-Cys-TBA (10.0 equiv) in $CDCl_3/CD_3OD$.

Figure S35. Mass spectrum for the reaction of (*R*)-**2** (2.0×10^{-2} M, 0.004 mmol in 200 μL $CDCl_3$) with *L*-Cys-TBA (10.0 equiv. 1.0×10^{-1} M in CD_3OD) ($CDCl_3/CD_3OD$=1/2).

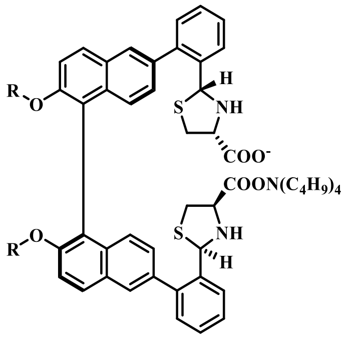

R=$CH_2CH_2CH_2C_8F_{17}$

o-(*R*)-**2**+2eq *L*-Cys-TBA

Chemical formular: $C_{78}H_{76}F_{34}N_3O_6S_2^-$

HRMS(ESI-IT-TOF) m/z: $[M]^-$ Calcd for $C_{78}H_{76}F_{34}N_3O_6S_2^-$: 1860.4638; Found: 1860.4853.

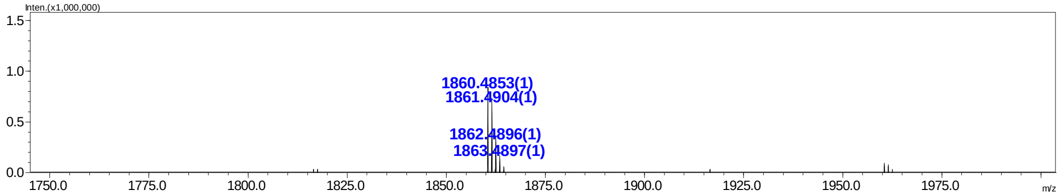

ESI-

4.2. Mass spectrum for the reaction of compound 4' with Mg^{2+} (2.0 equiv) in $MeOH/CH_2Cl_2$.

Figure S36. Mass spectrum for the reaction of the compound **4'** (2.0×10^{-3} in MeOH) with $MgCl_2$ (2.0 equiv. 4.0×10^{-3} M in MeOH), then diluted with CH_2Cl_2 ($MeOH/CH_2Cl_2$=1/1).

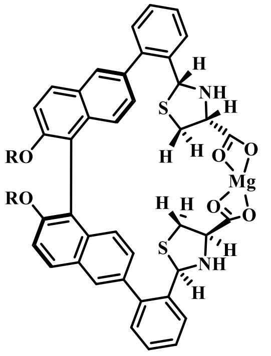

R = $(CH_2)_3C_8F_{17}$

Compound **6**

Chemical formular: $C_{62}H_{40}F_{34}MgN_2O_6S_2$

HRMS(ESI-IT-TOF) m/z: $[M+H]^+$ Calcd for $C_{62}H_{41}F_{34}MgN_2O_6S_2^+$: 1643.1708; Found: 1643.1715.

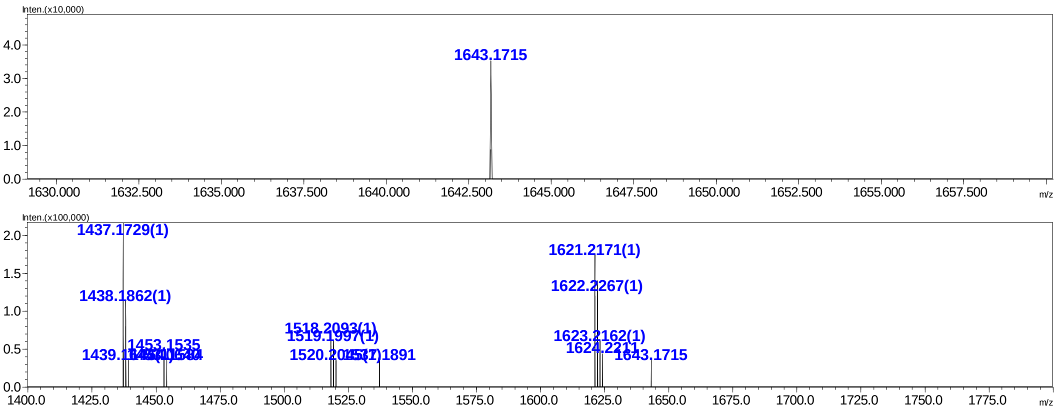

Inten.(x10,000)
1643.1715
m/z
Inten.(x100,000)
1437.1729(1)
1438.1862(1)
1453.1535
1518.2093(1)
1519.1997(1)
1621.2171(1)
1622.2267(1)
1623.2162(1)
1624.2211
1643.1715
m/z

ESI+

5. Fluorescence quantum yield measurements.

5.1. Sample preparation for fluorescence quantum yield measurement.

Stock solutions of compound **1** (5 × 10^{-3} mol/L in CH_2Cl_2)，compound **13** (5 × 10^{-3} mol/L in MeOH)，(*R*)-**2** (5 × 10^{-3} mol/L in CH_2Cl_2)，compound **4'** (5 × 10^{-3} mol/L in MeOH)，(*R*)-**3** (5 × 10^{-3} mol/L in MeOH)，compound **5'** (5 × 10^{-3} mol/L in MeOH)，compound **5** (5 × 10^{-3} mol/L in MeOH) and 5 × 10^{-2} mol/L TBA salts of amino acids were prepared. Amino acids-TBA salts were prepared by mixing amino acids and tetrabutylammonium hydroxide (TBAOH, 1 M in methanol) with the equivalent of the carboxylic groups in methanol in situ. For optical analysis, solutions of compound **1**, compound **13**, (*R*)-**2**, compound **4'**, (*R*)-**3**, compound **5'** and compound **5** (20 μL each) were added to several test tubes respectively which each contains 2.0 mL 2-(perfluorohexyl) ethyl alcohol (PEOH), CH_2Cl_2, or MeOH. Then a solution of a TBA salt of cysteine (16.0 equiv, 32 μL each) was added to test tubes that contain compound **1**, (*R*)-**2** or (*R*)-**3** to mix well. The resulting solution was placed in an incubator at 298 K for 120 min. Fluorescence quantum yield (Φ) measurement was conducted on Horiba JoblnYvon-Edision Fluorolog-3 under excitation wavelength of 300 nm.

Table S3. Fluorescence quantum yields of compound **1** (5 × 10^{-5} M), (*R*)-**2** (5 × 10^{-5} M) and (*R*)-**3** (5 × 10^{-5} M) with and without cysteine-TBA (16.0 equiv).

**Probe**	**Volume of probe added**	**Solvent**	**Volume of solvent**	**The equiv. of cysteine**	**Quantum yield (Φ_F, %)**
compound **1**	20 μL stock solution	CH_2Cl_2	2 mL	None	< 0.01 %
				16 equiv *D*-Cys	
				16 equiv *L*-Cys	
		PEOH		None	
				16 equiv D-Cys	3.92 %
				16 equiv L-Cys	5.83 %
(*R*)-**2**		CH_2Cl_2		None	< 0.01 %
				16 equiv D-Cys	
				16 equiv L-Cys	
		PEOH		None	
				16 equiv D-Cys	17.98 %
				16 equiv L-Cys	21.33 %
(*R*)-**3**		CH_2Cl_2		None	< 0.01 %
				16 equiv D-Cys	
				16 equiv L-Cys	
		MeOH		None	
				16 equiv D-Cys	0.85 %
				16 equiv L-Cys	0.82 %

Table S4. Fluorescence quantum yields of compound **13** (5×10^{-5} M), compound **5'** (5×10^{-5} M), compound **5** (5×10^{-5} M) and compound **4'** (5×10^{-5} M).

**Probe**	**Volume of probe added**	**Solvent**	**Volume of solvent**	**The equiv. of cysteine**	**Quantum yield (Φ_F, %)**
compound **13**	20 µL stock solution	CH_2Cl_2	2 mL	None	4.16 %
		PEOH			5.11 %
compound **4'**		CH_2Cl_2			3.04 %
		PEOH			9.42 %
compound **5'**		CH_2Cl_2			< 0.01 %
		MeOH			
compound **5**		CH_2Cl_2			
		MeOH			1.87 %

6. Molecular modeling study.

6.1. Computational details.

The theoretical calculations were performed via the Gaussian 16 suite of programs [S6]. The structures of the studied complexes were fully optimized at the B3LYP-D3BJ/6-31G(d,p) level of theory. The vibrational frequencies of the optimized structures were carried out at the same level. The structures were characterized as a local energy minimum on the potential energy surface by verifying that all the vibrational frequencies were real. The solvent (CH_3OH) effect was included in the calculations using the solvation model based on the density (SMD) model.

6.2. Molecular modeling study of methyl ether analog of compound 1.

Figure S37. HOMO and LUMO of methyl ether analog of compound **1**.

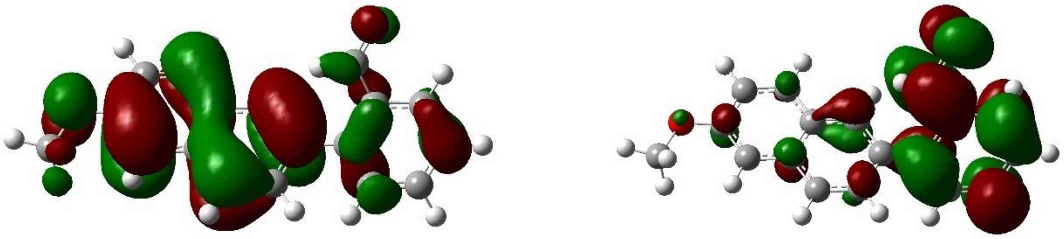

HOMO LUMO

Figure S38. Density functional calculation for the methyl ether analog of compound **1**.

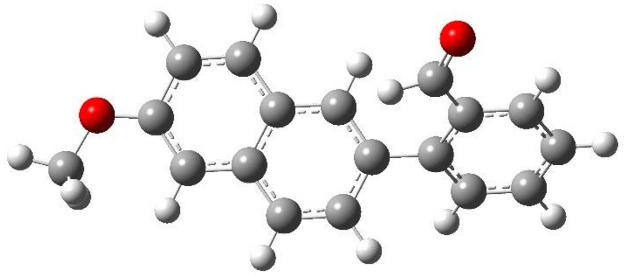

Calculation Type	FREQ
Calculation Method	RB3LYP-D3BJ
Basis Set	6-31G (D, P)
Charge	0
Spin	Singlet
Solvation	scrf=(smd,solvent=methanol)
Number of imaginary frequencies	0
E(RB3LYP)	-844.910513 Hartree

Standard orientation:

Center Number	Atomic Number	Atomic Type	Coordinates (Angstroms) X	Y	Z
1	6	0	0.963003	-0.564652	0.044955
2	6	0	0.191605	0.456341	-0.488768
3	6	0	-1.221026	0.433530	-0.409844
4	6	0	-1.872379	-0.668505	0.229388
5	6	0	-1.065658	-1.711558	0.759064
6	6	0	0.305057	-1.664536	0.665889
7	1	0	-1.536570	2.314301	-1.440737
8	1	0	0.666816	1.284278	-1.007132
9	6	0	-2.024225	1.475941	-0.951585
10	6	0	-3.289342	-0.697503	0.317102
11	1	0	-1.550939	-2.551355	1.248517
12	1	0	0.903859	-2.464223	1.090503
13	6	0	-4.032903	0.336900	-0.216335
14	6	0	-3.390994	1.432845	-0.857536
15	1	0	-4.011579	2.224673	-1.264829
16	8	0	-5.394241	0.414647	-0.188170
17	6	0	2.440218	-0.550492	-0.062042
18	6	0	3.105547	-1.703781	-0.508135
19	6	0	3.216073	0.599167	0.226799
20	6	0	4.484523	-1.712074	-0.696809

21	1	0	2.524668	-2.590499	-0.740916
22	6	0	4.605894	0.582435	0.015220
23	6	0	5.241065	-0.561551	-0.445570
24	1	0	4.971293	-2.615025	-1.053101
25	1	0	5.172687	1.478094	0.247904
26	1	0	6.315287	-0.567632	-0.600476
27	1	0	-3.761485	-1.540655	0.806538
28	6	0	-6.108237	-0.649603	0.444354
29	1	0	-5.834707	-0.735858	1.501971
30	1	0	-7.164731	-0.391609	0.360669
31	1	0	-5.923339	-1.604635	-0.060080
32	6	0	2.628742	1.797106	0.853096
33	8	0	3.212629	2.873300	0.952523
34	1	0	1.619846	1.676516	1.280893

6.3. Molecular modeling study of thiazolidine of the methyl ether analog of compound 1.

Figure S39. HOMO and LUMO of thiazolidine of methyl ether analog of compound **1**.

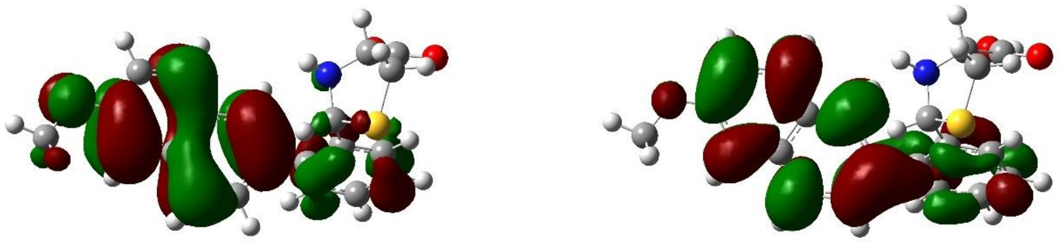

HOMO LUMO

Figure S40. Density functional calculation for the thiazolidine of methyl ether analog of compound **1**.

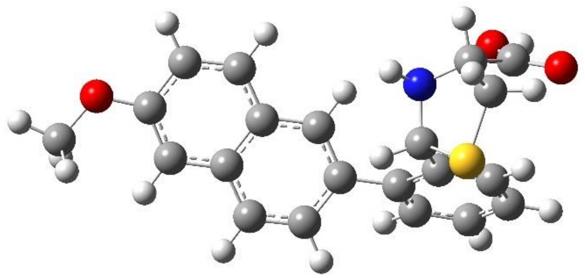

Calculation Type	FREQ
Calculation Method	RB3LYP-D3BJ
Basis Set	6-31G (D, P)
Charge	0
Spin	Singlet
Solvation	scrf=(smd,solvent=methanol)
Number of imaginary frequencies	0
E(RB3LYP)	-1490.471330 Hartree

Standard orientation:

Center Number	Atomic Number	Atomic Type	Coordinates (Angstroms) X	Y	Z
1	6	0	0.536937	1.332642	0.053013
2	6	0	1.026613	0.175662	-0.529917
3	6	0	2.398618	-0.166396	-0.455431
4	6	0	3.306570	0.698764	0.232849
5	6	0	2.788164	1.883738	0.819595
6	6	0	1.450435	2.191440	0.728935
7	1	0	2.229064	-2.013056	-1.577974
8	1	0	0.353434	-0.485209	-1.065078
9	6	0	2.912315	-1.352123	-1.052017

10	6	0	4.684264	0.359693	0.311939
11	1	0	3.466971	2.547200	1.348503
12	1	0	1.072202	3.097329	1.192909
13	6	0	5.141900	-0.800866	-0.279287
14	6	0	4.244626	-1.663921	-0.967982
15	1	0	4.644713	-2.566383	-1.419225
16	8	0	6.439744	-1.224163	-0.267879
17	6	0	-0.893676	1.731196	-0.051218
18	6	0	-1.176126	3.017498	-0.541907
19	6	0	-1.969308	0.895802	0.336684
20	6	0	-2.483480	3.474316	-0.680868
21	1	0	-0.348287	3.656751	-0.833586
22	6	0	-3.276575	1.379743	0.207525
23	6	0	-3.541625	2.646189	-0.308579
24	1	0	-2.672215	4.468915	-1.073916
25	1	0	-4.101496	0.760411	0.538420
26	1	0	-4.568485	2.986138	-0.403823
27	6	0	-1.684183	-0.492295	0.893496
28	1	0	-0.709530	-0.478284	1.377789
29	6	0	-3.560811	-2.413652	1.151041
30	6	0	-2.971920	-2.170021	-0.250560
31	1	0	-1.000382	-2.253724	0.170019
32	1	0	-3.206415	-3.368058	1.544116
33	1	0	-4.650988	-2.409123	1.138077
34	1	0	-2.838671	-3.128075	-0.766722
35	16	0	-2.935166	-1.042884	2.204723
36	7	0	-1.660807	-1.538916	-0.126137
37	6	0	-3.921937	-1.378966	-1.148104
38	8	0	-5.119554	-1.267212	-0.953969
39	8	0	-3.306453	-0.880709	-2.226791
40	1	0	-3.974539	-0.426342	-2.772359
41	1	0	5.353782	1.028012	0.839916
42	6	0	7.398539	-0.406260	0.405002
43	1	0	7.157653	-0.307634	1.469516
44	1	0	8.356632	-0.915842	0.294034
45	1	0	7.458933	0.589115	-0.049379

----

7. References.

S1. An, S.; Tang, G.; Zhong, Y.; Ma, L.; Liu, Q. Novel π-expanded chrysene-based axially chiral molecules: 1,1'-bichrysene-2,2'-diols and thiophene analogs. *J. Chem. Res*. **2020**, *44*, 641-645.

S2. Lv, N.; Xie, M.; Gu, W.; Ruan, H.; Qiu, S.; Zhou, C.; Cui, Z. Synthesis, properties, and structures of functionalized peri-xanthenoxanthene. *Org. Lett.* **2013**, 15, 2382-2385.

S3. Sun, Q.; Dai, Z.; Meng, X.; Xiao, F.-S. Homochiral porous framework as a platform for durability enhancement of molecular catalysts. *Chem. Mater.* **2017**, 29, 5720-5726.

S4. Bisht, R.; Chaturvedi, J.; Pandey, G.; Chattopadhyay, B. Double-fold ortho and remote C-H bond activation/borylation of BINOL: a unified strategy for arylation of BINOL. *Org. Lett.* **2019**, 21, 6476-6480.

S5. Jiang, L.; Tian, J.; Zhao, F.; Yu, S.; Shi, D.; Wang, X.; Yu, X.; Pu, L. Fluorescent Recognition of Functional Secondary Amines in the Fluorous Phase. *Eur. J. Org. Chem*. **2019**, *14*, 2533-2538.

S6. M. J. Frisch,; G. W. Trucks,; H. B. Schlegel, et al. Gaussian 16 Revision. A.03, Gaussian Inc., Wallingford, CT, **2016**.
